# Supplementary material for: Development and characterization of amino donor-acceptor Stenhouse adducts
Source: Nat Commun. 2024 Jul 1;15:5533. doi: 10.1038/s41467-024-49808-7 (PMC11217284; doi:10.1038/s41467-024-49808-7)
Supplement: Supplementary file 1 — Supplementary Information [file 41467_2024_49808_MOESM1_ESM.pdf]

Supplementary Information

Development and characterization of amino donor-acceptor Stenhouse adducts

Cesar A. Reyes[1], Hye Joon Lee[1], Connie Karanovic[1], Elias Picazo[1]*

[1] Department of Chemistry, Loker Hydrocarbon Research Institute, University of Southern California, 837 Bloom Walk, Los Angeles, California 90089-1661, United States.

*Corresponding author: Email: epicazo@usc.edu

This PDF includes:

Supplementary Methods
Supplementary Discussion
Supplementary Fig. 1–75
Supplementary Table 1
Supplementary References

Supplementary Information – Table of Contents

1. Supplementary Methods 2
A. DASA synthesis and aza-Piancatelli rearrangement 4
B. Experimental procedures 5
B.1. Amino DASA synthesis optimization 5
B.2. Synthesis of substrates 6
B.3. Synthesis of DASAs 16
C. UV-Vis absorbance measurements 22
C.1. Molar absorption coefficient and solvatochromic analysis 22
C.2. Solvatochromic slope comparison 28
C.3. Absorbance irradiation experiments 29
C.4. Photoswitching studies 38
C.5. Thermal stability evaluation 42
D. NMR *in situ* irradiation 44
E. Hammett value analysis 46
F. NMR spectra 47
2. Supplementary References 79

1. Supplementary Methods

Unless stated otherwise, reactions were conducted in flame-dried glassware using anhydrous solvents (freshly distilled or passed through activated alumina columns). All commercially obtained reagents were used as received unless otherwise specified. Furfural (98%), indoline (98%), and phenylhydrazine (97%) were obtained from Acros Organics B.V.B.A. Pyrrole-2-carboxaldehyde (99%) and *N*,*N*-dimethylbarbituric acid (99%) were obtained from Beantown Chemical Corporation. Thiophene-2-carboxaldehyde (>98%) and sodium hydride (60% dispersion in oil) were obtained from Tokyo Chemical Industry. Triethylamine (99%) and 4-dimethylaminopyridine (99%) were obtained from Thermo Fisher Scientific. Di-*tert*-butyl decarbonate (99%), pyridine (99%), and ethyl 4,4,4-trifluoroacetoacetate (99%) were obtained from Sigma-Aldrich. Acetic acid (glacial) and acetic anhydride (99%) were obtained from Ward's Science. 4-bromobenzenesulfonyl chloride (98%) was obtained from Apollo Scientific. 1-methyl-2-pyrrolecarboxaldehyde (98%) was obtained from Lancaster Synthesis Inc. Isoindoline hydrochloride (97%) was obtained from Ambeed. Hexafluoro-2-propanol (99%) was obtained from Chem-Impex International. Indium (III) bromide (99%) was obtained from STREM Chemicals. Chloroform-*d*, methylene chloride-d_2 (99.8%), and dimethyl sulfoxide-d_6 (99.9%) were obtained from Cambridge Isotope Laboratories. Furfural, indoline, and hexafluoro-2-propanol were freshly distilled prior to use. Isoindoline was prepared from isoindoline hydrdochloride by extracting isoindoline with an alkaline solution (sodium hydroxide). Reaction temperatures were controlled using IKA Plates (RCT digital) and the built-in temperature modulators. Thin layer chromatography (TLC) was conducted with EMD gel 60 F254 pre-coated plates (0.25 mm) and visualized using a combination of UV light, potassium permanganate, phosphomolybdic acid, and p-anisaldehyde staining. Silicycle Silica flash P60 (particle size 0.040–0.063 mm) was used for flash column chromatography. ^{1}H NMR spectra were recorded on a Mercury (400 MHz), or Varian spectrometers (500, 600 MHz) and are reported relative to deuterated solvent signals. Data for ^{1}H NMR spectra are reported as follows: chemical shift (δ ppm), multiplicity, coupling constant (Hz) and integration. ^{13}C NMR spectra were recorded on Mercury (100 MHz), or Varian spectrometers (125 MHz, 150 MHz) and are reported relative to deuterated solvent signals. IR data were collected on a Mettler Toledo ReactIR 702L equipped with a TE MCT detector, an AgX 6mm x 1.5m Fiber probe interface, and a DiComp diamond probe tip. All IR data are reported in terms of frequency absorption (cm^{-1}). Melting points were

recorded on a VWR melting point apparatus, and high resolution mass (HRMS) spectra were obtained on an Agilent 6545Q-TOF LC/MS. UV-visible spectral data was collected on an Agilent Cary 5000 UV-Vis-NIR Spectrophotometer with a UV quartz 10 mm pathlength cuvette (3.5 mL). Crystallographic data was collected on a Rigaku XtaLAB Synergy-S diffractometer.

Irradiation experiments were performed with a Dolan-Jenner Fiber-Lite Model 190, using an EKZ halogen bulb (10.8V, 30 Watt, 3100K) via a Dolan-Jenner BGT1826 fiber optic gooseneck on high output for broad band visible light. The convection-cooled temperature control was confirmed with a control experiment replicating DASA irradiation. Over the course of 12 hours of irradiation of toluene solution, the temperature changed from 26.2 ºC to 26.5 ºC without fluctuation.

NMR *in situ* irradiation experiment was set up following the protocol from Feldmeier *et al*. (Ref. *13*). The fiber-optic cable (M28L05; Ø400 µm, 0.39 NA, SMA-SMA Fiber Patch Cable, 5 meters), 590 nm Fiber-coupled LED (M590F3; 4.6 mW, FWHM = 18 nm), 490 nm Fiber-coupled LED (M490F4; 2.8 mW, FWHM = 22 nm), LED Driver (1200 mA), and power supply (KPS201) were purchased from Thor Labs. NMR tubes were purchased from Wilmad-LabGlass (SP Scienceware): WGS-5BL, Coaxial Insert for 5 mm NMR Sample Tube and 535-PP-7, 5 mm Thin Wall Precision NMR Sample Tube 7" L, 600MHz. Spectra were measured with a Varian spectrometer (600 MHz).

A. DASA synthesis and aza-Piancatelli rearrangement mechanism

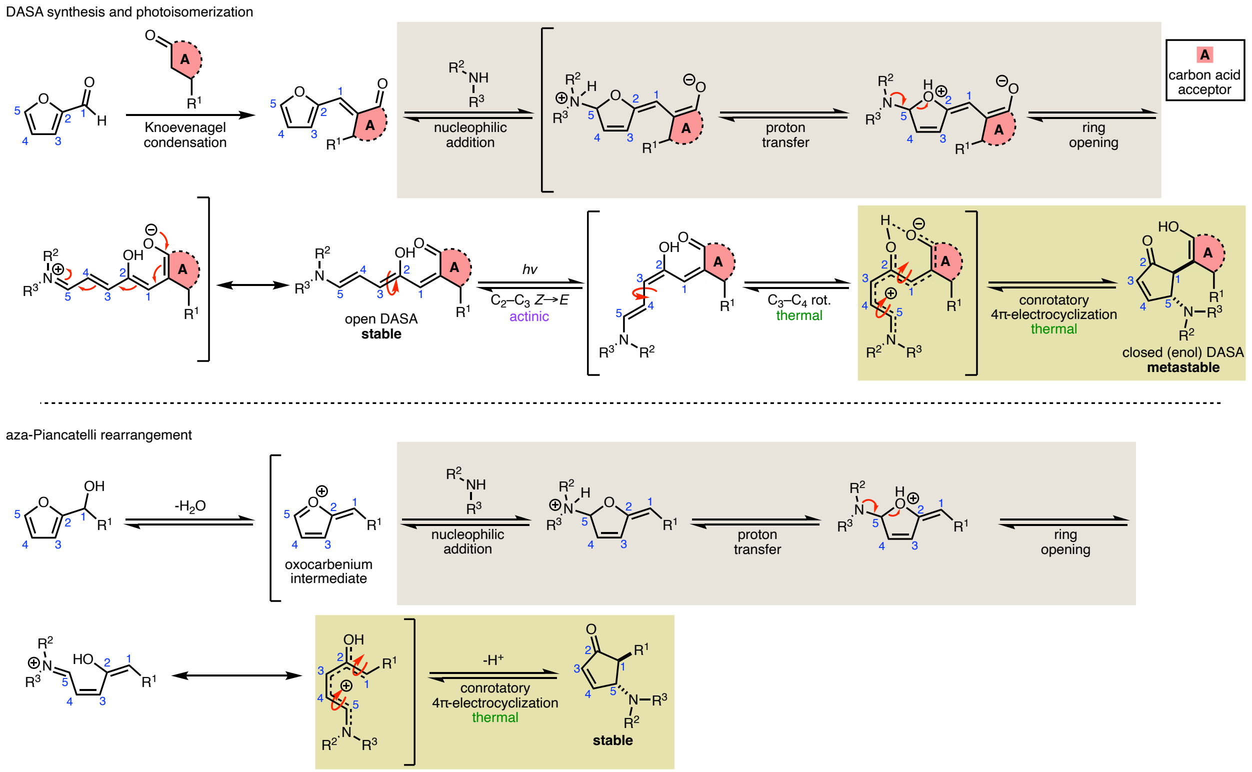

Supplementary Fig. 1. **Mechanistic comparison of DASA synthesis and photoisomerization to the aza-Piancatelli rearrangement.**[1–3]

B. Experimental procedures

B.1. Amino DASA synthesis optimization

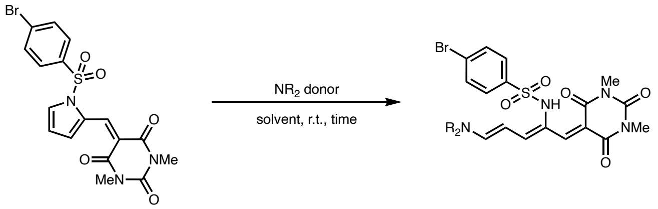

entry[a]	donor (equiv.)	solvent (conc.)	time (h)	observation	crude ^{1}H NMR conversion	purification[b]	yield
1	indoline (1.2)	neat	1.5	yellow to purple	15% DASA; 85% SM	none	n/a
2	indoline (1.2)	4:1 CH_2Cl_2:HFIP (0.6 M)	1.5	yellow to purple	49% DASA; 51% SM	CH_2Cl_2/hexanes recrystallization	39%
3	indoline (2.0)	4:1 CH_2Cl_2:HFIP (0.6 M)	2	yellow to purple	100% DASA; 0% SM	CH_2Cl_2/hexanes recrystallization	53%
4[c]	indoline (2.0)	4:1 CH_2Cl_2:HFIP (0.6 M)	0.5	yellow to purple	100% DASA; 0% SM	CH_2Cl_2/hexanes recrystallization	60%
5	isoindoline (2.0)	4:1 CH_2Cl_2:HFIP (0.6 M)	1	yellow to red	multiple products; no DASA	none	n/a
6	isoindoline (2.0)	CH_2Cl_2 (0.6 M)	0.5	yellow to red to brown	multiple products; no DASA	none	n/a
7	isoindoline (1.05)	CH_2Cl_2 (0.6 M)	0.3	yellow to red to brown	multiple products; no DASA	none	n/a
8	isoindoline (1.05)	CH_2Cl_2 (0.17 M)	4.5	yellow to red	DASA and significant byproducts	CH_2Cl_2/hexanes recrystallization	no improvement in purity
9	isoindoline (1.2)	CH_2Cl_2 (0.17 M)	1	yellow to red to brown	multiple byproducts and trace DASA	none	n/a
10	isoindoline (1.05)	4:1 CH_2Cl_2:HFIP (0.17 M)	3.5	yellow to red	>95% DASA and trace byproducts	CH_2Cl_2/hexanes recrystallization	degradation in purification
11	isoindoline (2.0)	4:1 CH_2Cl_2:HFIP (0.17 M)	1	yellow to red	>95% DASA and trace byproducts	degradation prior to purification[d]	n/a
purification optimization							
12	isoindoline (1.05)	4:1 CH_2Cl_2:HFIP (0.17 M)	2	yellow to red	>95% DASA and trace byproducts	CH_2Cl_2/acetone recrystallization (0 °C)	0 – 10%
13	isoindoline (1.05)	4:1 CH_2Cl_2:HFIP (0.17 M)	2	yellow to red	>95% DASA and trace byproducts	acetone trituration	10% (0 °C); 30% (r.t.)
14	isoindoline (1.05)	4:1 CH_2Cl_2:HFIP (0.17 M)	2	yellow to red	>95% DASA and trace byproducts	THF/Et_2O recrystallization	30%
15	isoindoline (1.05)	4:1 CH_2Cl_2:HFIP (0.17 M)	2	yellow to red	>95% DASA and trace byproducts	THF trituration	23%
16	isoindoline (1.05)	4:1 CH_2Cl_2:HFIP (0.17 M)	2	yellow to red	>95% DASA and trace byproducts	$CHCl_3$/Et_2O recrystallization	36%

Supplementary Table 1. **Amino DASA synthesis optimization.**

[a]All experiments were performed at 15–30 mg scale.

[b]Purifications were performed at room temperature unless stated otherwise.

[c]Activated pyrrole **S3** containing trifluoromethyl pyrazolone acceptor was used instead of activated pyrrole **4e**.

[d]Crude product turned brown before submitting to purification. ^{1}H NMR showed degradation with only trace levels of DASA.

Entry 3 represents the optimal conditions for synthesis of amino DASA **6e**

Entry 4 represents the optimal conditions for synthesis of amino DASA **8**

Entry 16 represents the optimal conditions for synthesis of amino DASA **7**

General considerations and notes

The difference in nucleophilicity of amine donors required tailoring of reaction conditions. A less nucleophilic donor, such as indoline, requires a higher concentration of nucleophile for greater conversion and yield. In contrast, a more nucleophilic donor, such as isoindoline, requires a lower concentration of amine to preserve the desired DASA product. For example, higher concentrations of isoindoline (greater than 0.17 M) led to the formation of **7**, which was then quickly decomposed nonspecifically to multiple byproducts, per TLC analysis. Achieving high conversion and low byproduct formation allowed for successful purification of amino DASA products. It should be noted that each DASA underwent solvent stability and solubility studies. We learned that each compound requires individualized purification optimization. Both recrystallization and trituration were found to be more efficient than column chromatography.

B.2. Synthesis of substrates

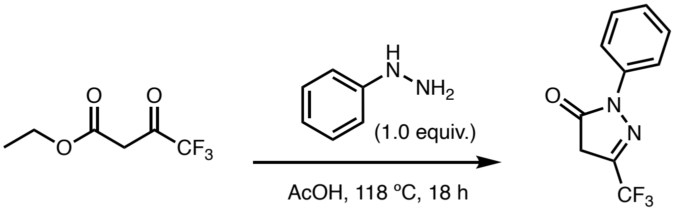

Supplementary Fig. 2. **Synthesis of trifluoromethyl pyrazolone S1.**

2-phenyl-5-(trifluoromethyl)-2,4-dihydro-3*H*-pyrazol-3-one (S1). In a 20 mL scintillation vial charged with a magnetic stir bar, ethyl 4,4,4,-trifluoroacetoacetate (1.46 mL, 10 mmol) was added to acetic acid (6 mL) followed by the addition of phenylhydrazine (984 μL, 10 mmol). The mixture stirred at reflux (118 °C) for 18 hours. After 18 hours, the reaction mixture was allowed to slowly cool to room temperature. Upon cooling, the crystallized product was vacuum filtered, washed with water and hexanes, then dried under high-vacuum overnight to provide trifluoromethyl pyrazolone **S1** (1.437 g, 63% yield) as a white solid. NMR characterization is of the enol form. **^{1}H NMR** (500 MHz, DMSO-d_6): δ = 7.71 – 7.69 (d, J = 7.8 Hz, 2H), 7.53 – 7.49 (t, J = 7.8 Hz, 2H), 7.40 – 7.37 (t, J = 7.7 Hz, 1H), 5.93 (s, 1H). Spectral data matches previously reported values.[4]

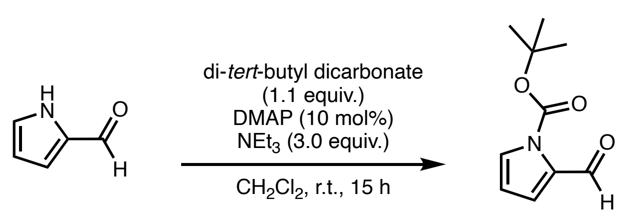

Supplementary Fig. 3. **Synthesis of pyrrole 2c.**

***tert*-butyl 2-formyl-1*H*-pyrrole-1-carboxylate (2c).** In a 20 mL scintillation vial charged with a magnetic stir bar, pyrrole-2-carboxaldehyde (238 mg, 2.5 mmol), di-*tert*-butyl dicarbonate (600 mg, 2.75 mmol), 4-dimethylaminopyridine (30.5 mg, 0.25 mmol), and triethylamine (1.05 mL, 7.5 mmol) were dissolved in CH_2Cl_2 (6 mL) and stirred until consumption of pyrrole-2-carboxaldehyde as monitored by TLC (4:1 hexanes:EtOAc). After 15 hours, the reaction mixture was quenched with water (5 mL). Then saturated sodium bicarbonate (5 mL) was added and the aqueous phase was extracted with CH_2Cl_2 (3 x 5 mL). The combined organic phases were washed with brine (10 mL), dried over anhydrous sodium sulfate, filtered, and concentrated under reduced pressure. The crude oil was purified by flash chromatography (9:1 hexanes:EtOAc) to provide pyrrole **2c** (420 mg, 86% yield) as a yellow oil. **^{1}H NMR** (500 MHz, $CDCl_3$): δ = 10.29 (s, 1H), 7.41 – 7.40 (t, J = 1.5 Hz, 1H), 7.14 (d, J = 1.8 Hz, 1H), 6.24 (t, J = 3.0 Hz, 1H), 1.61 (s, 9H). Spectral data matches previously reported values.[5]

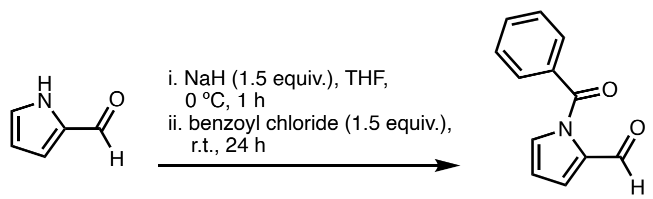

Supplementary Fig. 4. **Synthesis of pyrrole 2d.**

1-benzoyl-1*H*-pyrrole-2-carbaldehyde (2d). To a flame dried 100 mL round-bottom flask, a magnetic stir bar and NaH (300 mg, 7.50 mmol; 60% wt. in paraffin oil) were added. The flask was flushed with N_2, and after adding freshly distilled THF (50 mL, 0.1 M) and stirring, the mixture was cooled to 0 °C. Pyrrole-2-carboxaldehyde (476 mg, 5.0 mmol) was then dissolved in a minimal amount of THF (2 mL) and added dropwise. The mixture was then allowed to stir at 0

°C. After stirring for 1 hour, benzoyl chloride (1.05 g, 7.5 mmol) was added, and the mixture stirred at room temperature for 24 hours. Then saturated ammonium chloride (50 mL) was added and the aqueous phase was extracted with Et_2O (3 x 25 mL). The combined organic phase was washed with brine (50 mL), dried over anhydrous magnesium sulfate, filtered, and concentrated under reduced pressure. The crude oil was purified by flash chromatography (9:1 benzene:hexanes) to provide pyrrole **2d** (492 mg, 49% yield) as a white solid. **^{1}H NMR** (400 MHz, $CDCl_3$): δ = 9.94 (s, 1H), 7.71 – 7.69 (d, *J* = 7.7 Hz, 2H), 7.59 – 7.56 (t, *J* = 7.7 Hz, 1H), 7.46 – 7.42 (t, *J* = 7.1 Hz, 2H), 7.20 (t, *J* = 1.8 Hz, 1H), 7.13 (t, *J* = 1.4 Hz, 1H), 6.29 (s, 1H). Spectral data matches previously reported literature values.[6]

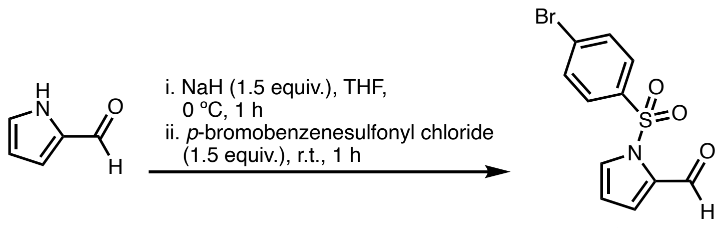

Supplementary Fig. 5. **Synthesis of pyrrole 2e.**

1-((4-bromophenyl)sulfonyl)-1*H*-pyrrole-2-carbaldehyde (2e). To a flame dried 100 mL round-bottom flask, a magnetic stir bar and NaH (316 mg, 7.89 mmol; 60% wt. in paraffin oil) were added. The flask was flushed with N_2, and after adding freshly distilled THF (50 mL 0.1M) and stirring, it was cooled to 0 °C. Pyrrole-2-carboxaldehyde (500 mg, 5.26 mmol) was then dissolved in a minimal amount of THF (3 mL) and added dropwise. The mixture was then allowed to stir at 0 °C. After stirring for 1 hour, 4-bromobenzenesulfonyl chloride (2.00 g, 7.89 mmol) was added, and the mixture stirred at room temperature for 1 hour. Then saturated ammonium chloride (50 mL) was added and the aqueous phase was extracted with Et_2O (3 x 25 mL). The combined organic phase was washed with brine (50 mL), dried over anhydrous magnesium sulfate, filtered, and concentrated under reduced pressure. The crude oil was purified by flash chromatography (9.6:0.3:0.1 → 9.4:0.5:0.1 → 8.9:1.0:0.1 hexanes:EtOAc:NEt_3) to provide pyrrole **2e** (1.51 g, 91% yield) as a white powder. **R_f**: 0.70 (59:40:1 hexanes:EtOAc:NEt_3); **mp**: 101–103 ºC; **^{1}H NMR** (500 MHz, $CDCl_3$): δ = 9.84 (s, 1H), 7.82 – 7.80 (m, *J* = 8.8 Hz, 2H), 7.68 – 7.66 (m, *J* = 8.7 Hz, 2H), 7.65 – 7.64 (dd, *J* = 3.3, 1.8 Hz, 1H), 7.17 – 7.16 (dd, *J* = 3.8, 1.8 Hz, 1H), 6.44 – 6.43 (t, *J*

= 3.4 Hz, 1H); **^{13}C NMR** (125 MHz, $CDCl_3$): δ = 178.4, 137.1, 133.6, 132.9, 130.1, 129.9, 129.3, 126.3, 112.8; **IR** (acetone, cm^{-1}): 1684, 1572, 1388, 1184, 1156, 1088, 1060, 1008, 828, 760, 740; **HRMS-QToF-ESI** (*m/z*): $[M + H]^+$ calcd. for $C_{11}H_9BrNO_3S^+$, 313.9481; found, 313.9492.

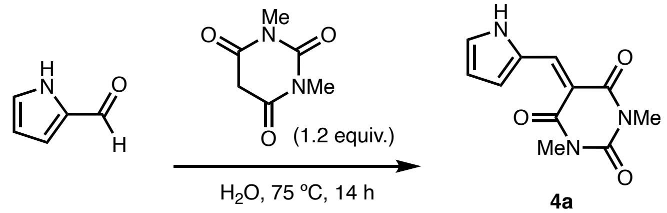

4a

Supplementary Fig. 6. **Synthesis of activated pyrrole 4a.**

5-((1*H*-pyrrol-2-yl)methylene)-1,3-dimethylpyrimidine-2,4,6(1*H*,3*H*,5*H*)-trione (4a). In a 50 mL round-bottom flask charged with a magnetic stir bar, pyrrole-2-carboxaldehyde (238 mg, 2.5 mmol) and *N,N*-dimethylbarbituric acid (468 mg, 3.0 mmol) were dissolved in H_2O (25 mL). The mixture stirred at 75 °C for 14 hours. After cooling to room temperature, the yellow precipitant was filtered under vacuum, washed with hexanes, and allowed to dry under high vacuum to provide activated pyrrole **4a** (519 mg, 89% yield) as a yellow solid. **^{1}H NMR** (600 MHz, $CDCl_3$): δ = 13.28 (br, 1H), 8.34 (s, 1H), 7.41 (s, 1H), 7.14 (t, J = 1.8 Hz, 1H), 6.54 (m, 1H), 3.42 (s, 3H), 3.40 (s, 3H); **^{13}C NMR** (150 MHz, $CDCl_3$): δ = 163.9, 163.5, 151.7, 143.2, 131.4, 130.2, 129.7, 114.7, 105.8, 29.0, 28.5. Spectral data matches previously reported values.[7]

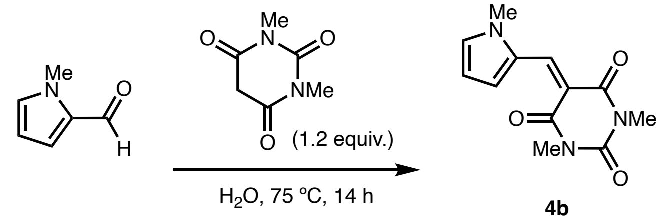

4b

Supplementary Fig. 7. **Synthesis of activated pyrrole 4b.**

1,3-dimethyl-5-((1-methyl-1*H*-pyrrol-2-yl)methylene)pyrimidine-2,4,6(1*H*,3*H*,5*H*)-trione (4b). In a 25 mL round-bottom flask charged with a magnetic stir bar, 1-methyl-2-pyrrolecarboxaldehyde (108 µL, 1.0 mmol) and *N,N*-dimethylbarbituric acid (187 mg, 1.2 mmol) were dissolved in H_2O (10 mL). The mixture stirred at 75 °C for 14 hours. After cooling to room temperature, the yellow precipitant was filtered under vacuum, washed with hexanes, and allowed

to dry under high vacuum to provide activated pyrrole **4b** (226 mg, 91% yield) as a yellow solid. **R*f***: 0.16 (4:1 hexanes:EtOAc); **mp**: 205–209 °C; **^{1}H NMR** (600 MHz, $CDCl_3$): δ = 8.60 – 8.59 (d, *J* = 4.3 Hz, 1H), 8.44 (s, 1H), 7.20 (s, 1H), 6.43 – 6.42 (dd, *J* = 4.3, 2.2 Hz, 1H), 3.89 (s, 3H), 3.40 (s, 3H), 3.39 (s, 3H); **^{13}C NMR** (150 MHz, $CDCl_3$): δ = 164.1, 161.3, 151.9, 139.9, 135.8, 130.0, 128.6, 112.6, 107.0, 35.1, 28.9, 28.2; **IR** (acetone, cm^{-1}): 1664, 1564, 1492, 1392, 1348, 1304, 1180, 1064, 788, 756, 704; **HRMS-QToF-ESI** (*m/z*): $[M + H]^+$ calcd. for $C_{12}H_{14}N_3O_3^+$, 248.1030; found, 248.1036.

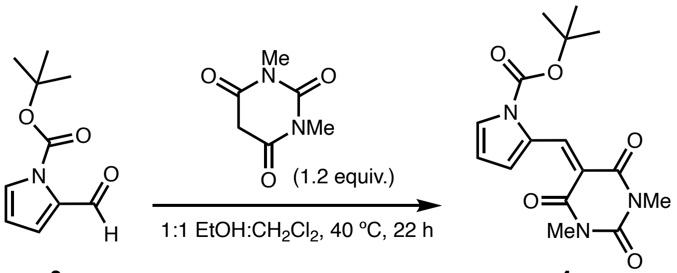

Supplementary Fig. 8. **Synthesis of activated pyrrole 4c.**

tert-butyl 2-((1,3-dimethyl-2,4,6-trioxotetrahydropyrimidin-5(2*H*)-ylidene)methyl)-1*H*-pyrrole-1-carboxylate (4c). In a 20 mL scintillation vial charged with a magnetic stir bar, pyrrole **2c** (137 mg, 0.7 mmol) and *N*,*N*-dimethylbarbituric acid (131 mg, 0.84 mmol) were added to 1:1 EtOH:CH_2Cl_2 (7 mL) and stirred at 40 °C. After 22 hours, the reaction mixture was allowed to cool to room temperature and quenched with water (10 mL). The aqueous phase was extracted with CH_2Cl_2 (3 x 10 mL). The combined organic phases were washed with brine (10 mL), dried over anhydrous magnesium sulfate, filtered, and concentrated under reduced pressure. The crude solid was purified by flash chromatography (8:2 hexanes:EtOAc) to provide activated pyrrole **4c** (216 mg, 93% yield) as a yellow solid. **R*f***: 0.41 (4:1 hexanes:EtOAc); **mp**: 141–143 °C; **^{1}H NMR** (500 MHz, $CDCl_3$): δ = 9.28 (s, 1H), 8.39 – 8.38 (d, *J* = 2.5 Hz, 1H), 7.72 (m, 1H), 6.44 – 6.43 (t, *J* = 3.4 Hz, 1H), 3.40 (s, 3H), 3.38 (s, 3H), 1.64 (s, 9H); **^{13}C NMR** (125 MHz, $CDCl_3$): δ = 163.3, 161.1, 151.7, 148.7, 144.9, 131.3, 130.3, 129.9, 113.0, 111.7, 86.4, 29.1, 28.4, 28.1; **IR** (acetone, cm^{-1}): 1748. 1672, 1572, 1464, 1428, 1428, 1380, 1316, 1260, 1148, 1128, 1076, 844, 792, 760, 724, 700, 668, 660; **HRMS-QToF-ESI** (*m/z*): $[M – C_5H_8O_2 + H]^+$ calcd. for $C_{11}H_{12}N_3O_3^+$, 234.0873; found, 234.0891.

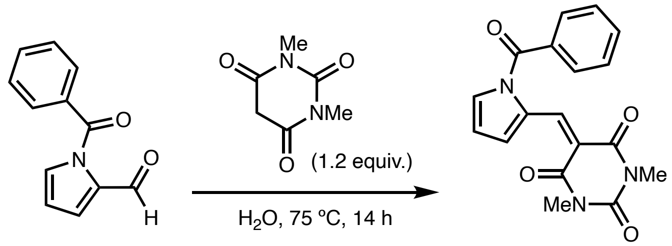

Supplementary Fig. 9. **Synthesis of activated pyrrole 4d.**

5-((1-benzoyl-1*H*-pyrrol-2-yl)methylene)-1,3-dimethylpyrimidine-2,4,6(1*H*,3*H*,5*H*)-trione (4d). In a 25 mL round-bottom flask charged with a magnetic stir bar, pyrrole **2d** (199 mg, 1.0 mmol) and *N,N*-dimethylbarbituric acid (187 mg, 1.2 mmol) were dissolved in H_2O (10 mL). The mixture stirred at 75 °C for 14 hours. After cooling to room temperature, the yellow precipitant was filtered under vacuum, washed with hexanes, and allowed to dry under high vacuum to provide activated pyrrole **4d** (320 mg, 95% yield) as a yellow solid. **R*f***: 0.17 (4:1 hexanes:EtOAc); **mp**: 164–168 °C; **^{1}H NMR** (500 MHz, $CDCl_3$): δ = 8.79 (s, 1H), 8.36 (d, *J* = 3.7 Hz, 1H), 7.83 – 7.81 (d, *J* = 7.9 Hz, 2H), 7.69 – 7.66 (t, *J* = 7.2 Hz, 1H), 7.56 – 7.52 (t, *J* = 7.8 Hz, 2H), 7.35 (m, 1H), 6.49 – 6.48 (t, *J* = 3.5 Hz, 1H), 3.38 (s, 3H), 3.36 (s, 3H); **^{13}C NMR** (125 MHz, $CDCl_3$): δ = 168.4, 163.0, 161.1, 151.7, 144.2, 133.9, 132.9, 132.1, 130.7, 130.7, 130.5, 128.9, 113.3, 112.1, 29.1, 28.4; **IR** (acetone, cm^{-1}): 1672, 1572, 1464, 1424, 1376, 1344, 1312, 1280, 1252, 1164, 1136, 1092, 1032, 872, 792, 756, 724, 696, 676; **HRMS-QToF-ESI** (*m/z*): $[M + Na]^+$ calcd. for $C_{18}H_{15}N_3O_4Na^+$, 360.0955; found, 360.0976.

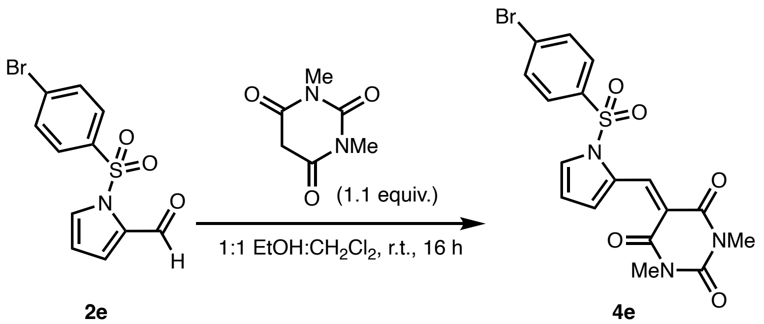

Supplementary Fig. 10. **Synthesis of activated pyrrole 4e.**

5-((1-((4-bromophenyl)sulfonyl)-1*H*-pyrrol-2-yl)methylene)-1,3-dimethylpyrimidine-2,4,6(1*H*,3*H*,5*H*)-trione (4e). In a 10 mL round-bottom flask charged with a magnetic stir bar, pyrrole **2e** (390 mg, 1.241 mmol) and *N,N*-dimethylbarbituric acid (213 mg, 1.365 mmol) were dissolved in 1:1 EtOH:CH_2Cl_2 (5 mL) and stirred at room temperature. After 16 hours, the yellow precipitant was filtered under vacuum, washed with hexanes, and allowed to dry under high vacuum to provide activated pyrrole **4e** (560 mg, 99% yield) as a yellow solid. **R_f**: 0.60 (59:40:1 hexanes:EtOAc:NEt_3); **mp**: 221–223 °C; **^{1}H NMR** (500 MHz, $CDCl_3$): δ = 9.07 (s, 1H), 8.61 – 8.60 (dd, *J* = 4.1, 1.6 Hz, 1H), 7.88 – 7.88 (dd, *J* = 3.2, 1.6 Hz, 1H), 7.85 – 7.82 (d, *J* = 8.5 Hz, 2H), 7.69 – 7.66 (d, *J* = 8.5 Hz, 2H), 6.57 – 6.55 (t, *J* = 3.7 Hz, 1H) 3.42 (s, 3H), 3.34 (s, 3H); **^{13}C NMR** (125 MHz, $CDCl_3$): δ = 162.9, 160.7, 151.4, 140.4, 137.2, 133.3, 132.3, 131.6, 130.5, 129.6, 129.1, 114.3, 112.4, 29.2, 28.4; **IR** (acetone, cm^{-1}): 1672, 1576, 1468, 1376, 1348, 1180, 1148, 1132, 1064 , 1012, 792, 748, 668; **HRMS-QToF-ESI** (*m/z*): $[M + H]^+$ calcd. for $C_{17}H_{15}BrN_3O_5S^+$, 451.9910; found, 451.9919.

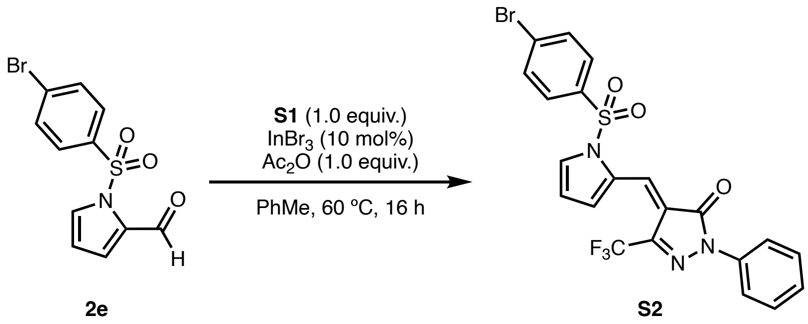

Supplementary Fig. 11. **Synthesis of activated pyrrole S2.**

(*E*)-4-((1-((4-bromophenyl)sulfonyl)-1*H*-pyrrol-2-yl)methylene)-2-phenyl-5-(trifluoromethyl)-2,4-dihydro-3*H*-pyrazol-3-one (S2. Following a modified procedure from Ref. 8: In a 4 mL dram vial charged with a magnetic stir bar, pyrrole **2e** (223 mg, 0.71 mmol), trifluoromethyl pyrazolone **S1** (178 mg, 0.781 mmol), and $InBr_3$ (25 mg, 0.071 mmol) were dissolved in toluene (710 µL). Acetic anhydride (67 µL, 0.71 mmol) was subsequently added and the mixture was stirred at 60 °C for 16 hours. After cooling to room temperature, the mixture was diluted with EtOAc (5 mL), quenched with saturated sodium bicarbonate (5 mL), and the aqueous phase was extracted with EtOAc (3 x 5 mL). Then the combined organic phases were washed with brine (5 mL), dried over anhydrous sodium sulfate, filtered, and concentrated under reduced pressure. The crude solid was purified by flash chromatography (79.5:20.0:0.5 hexanes:EtOAc:NEt_3) to provide activated pyrrole **S2** (270 mg, 73%) as an orange solid. $\mathbf{R}_f$: 0.50 (79.5:20.0:0.5 hexanes:acetone:NEt_3); **mp**: 160–166 °C; **^{1}H NMR** (600 MHz, CD_2Cl_2): δ = 8.99 – 8.98 (dd, J = 4.1, 1.6 Hz, 1H), 8.36 (s, 1H), 7.91 – 7.90 (dd, J = 3.1, 1.5 Hz, 1H), 7.86 – 7.84 (d, J = 8.7 Hz, 2H), 7.69 (s, 4H), 7.41 – 7.44 (t, J = 8.6 Hz, 2H), 7.27 – 7.24 (t, J = 7.4 Hz, 1H), 6.66 – 6.65 (t, J = 4.0 Hz, 1H); **^{13}C NMR** (150 MHz, CD_2Cl_2): δ = 161.1, 137.8, 136.9, 133.4, 133.1, 131.8, 131.2, 130.6, 129.8, 128.9, 128.9, 128.5, 126.2, 121.1, 119.9, 119.4, 116.7, 115.2, 29.8; **IR** (acetone, cm^{-1}): 1704, 1644, 1596, 1576, 1508, 1476, 1384, 1264, 1184, 1144, 1068, 1008, 964, 876, 816, 748, 716, 696, 668; **HRMS-QToF-ESI** (*m/z*): $[M + H]^+$ calcd. for $C_{21}H_{14}BrF_3N_3O_3S^+$, 523.9886; found, 523.9916.

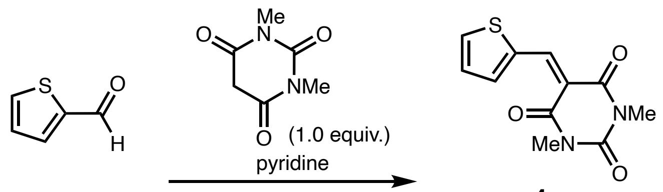

$\mathbf{4_{thio}}$

Supplementary Fig. 12. **Synthesis of activated thiophene $\mathbf{4_{thio}}$.**

1,3-dimethyl-5-(thiophen-2-ylmethylene)pyrimidine-2,4,6(1*H*,3*H*,5*H*)-trione ($\mathbf{4_{thio}}$). In a 20 mL scintillation vial charged with a magnetic sir bar, thiophene-2-carboxaldehyde (935 µL, 10.0 mmol), *N,N*-dimethylbarbituric acid (1.561 g, 10.0 mmol), and 1 drop of pyridine were added to EtOH (10 mL). The mixture stirred at 80 °C for 18 h. After cooling to room temperature, the precipitant was filtered under vacuum, washed with hexanes, and allowed to dry under high vacuum to provide activated thiophene $\mathbf{4_{thio}}$ (1.984 g, 79%) as a yellow solid. **^{1}H NMR** (600 MHz, $CDCl_3$): δ = 8.75 (s, 1H), 8.01 – 8.00 (d, *J* = 5.0 Hz, 1H), 7.90 (d, *J* = 3.8 Hz, 1H), 7.29 – 7.28 (t, *J* = 4.5 Hz, 1H), 3.42 (d, 6H); **^{13}C NMR** (150 MHz, $CDCl_3$): δ = 162.8, 161.9, 151.5, 149.2, 145.6, 142.0, 137.1, 128.4, 110.7, 29.1, 28.3. Spectral data matches previously reported values.[9]

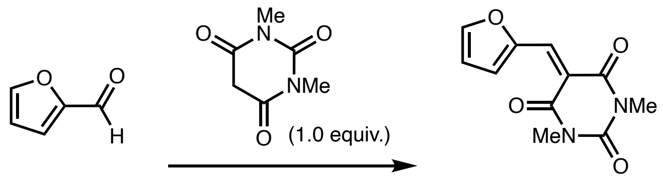

$\mathbf{4_{fur}}$

Supplementary Fig. 13. **Synthesis of activated furan $\mathbf{4_{fur}}$.**

5-(furan-2-ylmethylene)-1,3-dimethylpyrimidine-2,4,6(1*H*,3*H*,5*H*)-trione ($\mathbf{4_{fur}}$). In a 100 mL round-bottom flask charged with a magnetic stir bar, furfural (828 µL, 10.0 mmol) and *N,N*-dimethylbarbituric acid (1.561 g, 10.0 mmol) were dissolved in H_2O (40 mL) and stirred at room temperature. After 22 hours, the yellow precipitant was filtered under vacuum, washed with hexanes, and allowed to dry under high vacuum to provide activated furan $\mathbf{4_{fur}}$ (2.243 g, 96%) as a yellow solid. **^{1}H NMR** (600 MHz, $CDCl_3$): δ = 8.64 – 8.63 (d, *J* = 3.8 Hz, 1H), 8.43 (s, 1H), 7.85 (dd, *J* = 1.7, 0.7 Hz, 1H), 6.74 – 6.73 (ddd, *J* = 3.9, 1.7, 0.8 Hz, 1H), 3.41 (s, 3H), 3.40 (s, 3H); **^{13}C NMR** (150 MHz, $CDCl_3$): δ = 162.6, 160.9, 151.5, 151.3, 150.5, 141.1, 128.2, 115.3, 111.6, 29.1, 28.4. Spectral data matches previously reported values.[4]

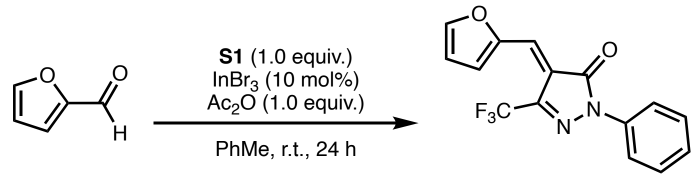

Supplementary Fig. 14. **Synthesis of activated furan S3.**

(*E*)-4-(furan-2-ylmethylene)-2-phenyl-5-(trifluoromethyl)-2,4-dihydro-3*H*-pyrazol-3-one (S3. Following a modified procedure from Ref. 8: In a 4 mL dram vial charged with a magnetic stir bar, furfural (83 µL, 1.0 mmol), trifluoromethyl pyrazolone **S1** (228 mg, 1.0 mmol), acetic anhydride (95 µL, 1.0 mmol), and $InBr_3$ (36 mg 0.1 mmol) were dissolved in toluene (1 mL) and stirred at room temperature. After 24 hours, the mixture was concentrated under reduced pressure. The crude solid was purified by flash chromatography (1:1 hexanes:CH_2Cl_2) to provide activated furan **S3** (84 mg, 27%) as an orange solid. **^{1}H NMR** (600 MHz, $CDCl_3$): δ = 8.92 (d, *J* = 3.8 Hz, 1H), 7.94 – 7.92 (dd, *J* = 8.7, 1.1 Hz, 2H), 7.87 (dd, *J* = 1.7 Hz, 0.6 Hz, 1H), 7.69 (s, 1H), 7.47 – 7.44 (t, *J* = 7.4 Hz, 2H), 7.29 – 7.26 (t, *J* = 7.6 Hz, 1H), 6.80 – 6.79 (ddd, *J* = 3.9, 1.7, 0.8 Hz, 1H). Spectral data matches previously reported values.[4]

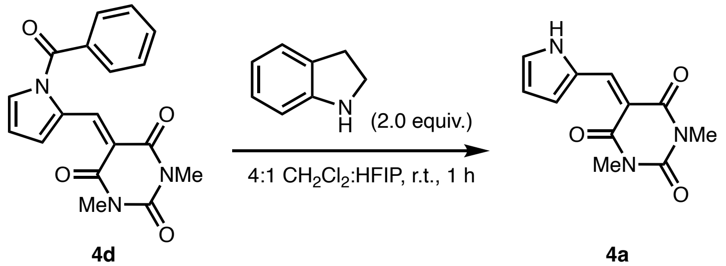

Supplementary Fig. 15. **Formation of pyrrole 4a from 4d.**

5-((1*H*-pyrrol-2-yl)methylene)-1,3-dimethylpyrimidine-2,4,6(1*H*,3*H*,5*H*)-trione (4a). In a 4 mL dram vial charged with a magnetic stir bar, activated pyrrole **4d** (30 mg, 0.089 mmol) was dissolved in 4:1 CH_2Cl_2:HFIP (0.22 mL). Indoline (20 µL, 0.178 mmol) was subsequently added and the mixture was stirred at room temperature. After 1 hour the mixture was concentrated under reduced pressure to obtain the crude residue. 99% yield of **4a** was observed in ^{1}H NMR of the crude residue using 1,3,5-trimethoxybenzene as the internal standard.

B.3. Synthesis of DASAs

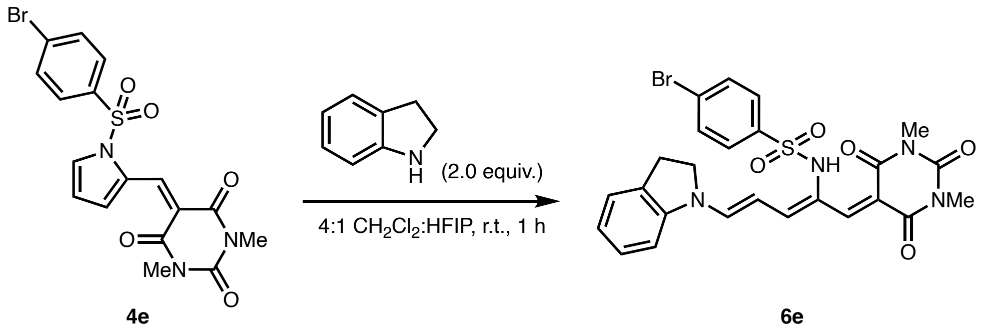

Supplementary Fig. 16. **Synthesis of amino DASA 6e.**

4-bromo-*N*-((2*Z*,4*E*)-1-(1,3-dimethyl-2,4,6-trioxotetrahydropyrimidin-5(2*H*)-ylidene)-5-(indolin-1-yl)penta-2,4-dien-2-yl)benzenesulfonamide (6e). In a 4 mL dram vial charged with a magnetic stir bar, activated pyrrole **4e** (300 mg, 0.663 mmol) was dissolved in 4:1 CH_2Cl_2:HFIP (1.1 mL). Indoline (150 µL, 1.326 mmol) was subsequently added and the mixture was stirred at room temperature. An immediate color change to purple was observed. After 1 hour the mixture was concentrated under reduced pressure, and the crude residue was recrystallized using CH_2Cl_2 as solvent and hexanes as antisolvent to provide amino DASA **6e** (280 mg, 74% yield) as a purple solid. Crystals of **6e** suitable for X-ray diffraction analysis were obtained by recrystallization via vapor diffusion of Et_2O into a solution of **6e** in $CHCl_3$. **R*f***: 0.40 (97:2:1 CH_2Cl_2:MeOH:NEt_3); **^{1}H NMR** (600 MHz, $CDCl_3$): δ = 10.06 (s, 1H), 7.89 – 7.87 (d, *J* = 12.3 Hz, 1H), 7.65 (s, 1H), 7.51 – 7.50 (d, *J* = 8.4 Hz, 2H), 7.44 – 7.41 (m, 3H), 7.33 – 7.32 (t, *J* = 8.4 Hz, 2H), 7.20 – 7.15 (m, 2H), 6.65 – 6.61 (t, *J* = 12.4 Hz, 1H), 4.27 –4.24 (t, *J* = 8.0 Hz, 2H), 3.38 – 3.35 (t, *J* = 8.0 Hz, 2H), 3.29 (s, 3H), 3.29 (s, 3H); **^{13}C NMR** (150 MHz, $CDCl_3$): δ = 162.9, 160.4, 152.5, 151.4, 146.2, 141.7, 138.1, 133.2, 131.6, 129.1, 128.7, 127.8, 126.6, 126.5, 124.8, 110.4, 107.5, 101.9, 49.8, 29.9, 28.7, 28.4, 27.6; **IR** (HFIP, cm^{-1}): 1684, 1600, 1580, 1528, 1444, 1392, 1288, 1240, 1224, 1188, 1176, 1128, 1008, 980, 864, 780, 760, 704, 684, 672, 664; **UV-Vis** (CH_2Cl_2): λ_{max} = 578 nm; **HRMS-QToF-ESI** (*m/z*): $[M + Na]^+$ calcd. for $C_{25}H_{23}BrN_4O_5SNa^+$, 593.0465; found, 593.0483.

Crystal structure analysis

Diffraction intensities were collected at 100 K on a Rigaku XtaLAB Synergy-S diffractometer equipped with an HyPix-600HE detector and an Oxford Cryostream 800 low temperature unit, using Cu K_{α} PhotonJet-S X-ray source, 1.54164 Å. The frames were integrated using the SAINT algorithm to give the hkl files. Data were corrected for absorption effects using the multi-scan method (SADABS) with Rigaku CrysalisPro. The structures were solved by intrinsic phasing and refined with the SHELXTL Software Package. Deposition Number 2312996 contains the supplementary crystallographic data for this paper. These data are provided free of charge by the joint Cambridge Crystallographic Data Centre and Fachinformationszentrum Karlsruhe Access Structures service www.ccdc.cam.ac.uk/structures.

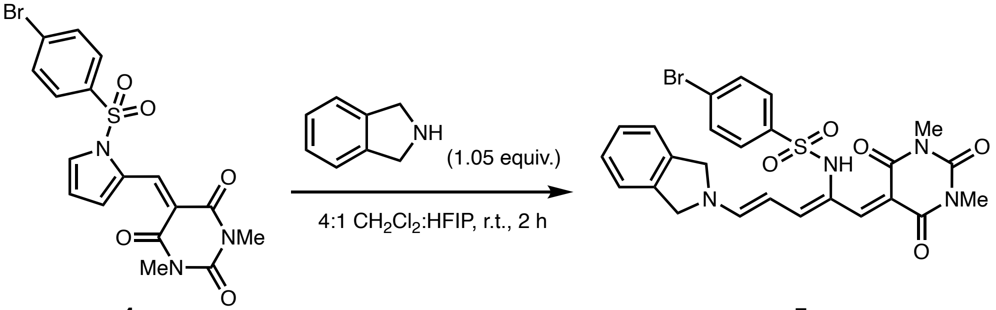

4e 7

Supplementary Fig. 17. **Synthesis of amino DASA 7.**

4-bromo-*N*-((2*Z*,4*E*)-1-(1,3-dimethyl-2,4,6-trioxotetrahydropyrimidin-5(2*H*)-ylidene)-5-(isoindolin-2-yl)penta-2,4-dien-2-yl)benzenesulfonamide (7). In a 4 mL dram vial charged with a magnetic stir bar, activated pyrrole **4e** (100 mg, 0.221 mmol) was dissolved in 4:1 CH_2Cl_2:HFIP (1.3 mL). Isoindoline (26 µL, 0.232 mmol) was subsequently added and the mixture was stirred at room temperature. An immediate color change to orange was observed and the solution then turned dark red over time. After 2 hours the mixture was concentrated under reduced pressure, and the crude residue was recrystallized using $CHCl_3$ as solvent and Et_2O as antisolvent to provide amino DASA **7** (55 mg, 43% yield) as a red solid. $\mathbf{R}_f$: 0.40 (97:2:1 CH_2Cl_2:MeOH:NEt_3); **^{1}H NMR** (400 MHz, $CDCl_3$): δ = 10.09 (s, 1H), 7.70 – 7.67 (d, J = 12.2 Hz, 1H), 7.59 (s, 1H), 7.51 – 7.48 (d, J = 8.6 Hz, 2H), 7.43 – 7.30 (m, 7H), 6.56 – 6.50 (t, J = 12.4 Hz, 1H), 5.11 (s, 2H), 4.99 (s, 2H), 3.29 (s, 3H), 3.28 (s, 3H); **^{13}C NMR** (125 MHz, $CDCl_3$): δ = 163.1, 162.9, 161.8, 156.6, 152.1, 151.6, 138.1, 134.4, 133.9, 131.5, 129.0, 128.8, 127.8, 123.5, 123.2, 122.9, 122.7, 106.9, 100.3,

58.8, 55.3, 28.7, 28.3; **IR** (CH_2Cl_2, cm^{-1}): 1788, 1644, 1620, 1608, 1476, 1448, 1368, 1308, 1276, 1240, 1200, 1148, 1084, 1012, 976, 940, 904, 856, 780, 748, 684, 672; **UV-Vis** (CH_2Cl_2): λ_{max} = 531 nm; **HRMS-QToF-ESI** (*m/z*): $[M + H]^+$ calcd. for $C_{25}H_{24}BrN_4O_5S^+$, 571.0645; found, 571.0674.

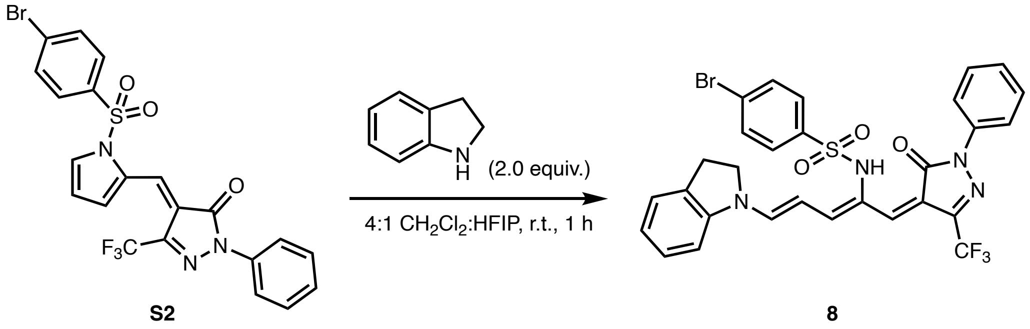

Supplementary Fig. 18. **Synthesis of amino DASA 8.**

4-bromo-*N*-((1*Z*,2*Z*,4*E*)-5-(indolin-1-yl)-1-(5-oxo-1-phenyl-3-(trifluoromethyl)-1,5-dihydro-4*H*-pyrazol-4-ylidene)penta-2,4-dien-2-yl)benzenesulfonamide (8). In a 4 mL dram vial charged with a magnetic stir bar, activated pyrrole **S2** (100 mg, 0.191 mmol) was dissolved in 4:1 CH_2Cl_2:HFIP (320 mL). Indoline (43 µL, 0.382 mmol) was subsequently added and the mixture was stirred at room temperature. An immediate color change to purple was observed. After 1 hour the mixture was concentrated under reduced pressure, and the crude residue was recrystallized using CH_2Cl_2 as solvent and hexanes as antisolvent to provide amino DASA **8** (107 mg, 87% yield) as a blue solid. **R_f**: 0.60 (97:2:1 CH_2Cl_2:MeOH:NEt_3); **^{11}H NMR** (500 MHz, $CDCl_3$): δ = 11.00 (br, 1H), 7.97 – 7.95 (d, *J* = 12.1 Hz, 1H), 7.92 – 7.90 (d, *J* = 7.4 Hz, 2H), 7.53 – 7.52 (d, *J* = 8.6 Hz, 2H), 7.44 – 7.41 (t, *J* = 7.7, 2H), 7.38 – 7.19 (m, 7H), 7.15 – 7.14 (d, *J* = 8.0 Hz, 1H), 6.86 (s, 1H), 6.74 – 6.69 (t, *J* = 12.4 Hz, 1H), 4.30 – 4.27 (t, *J* = 7.8 Hz, 2H), 3.38 – 3.35 (t, *J* = 7.8 Hz, 2H); **^{13}C NMR** (125 MHz, $CDCl_3$): δ = 162.7, 159.1, 147.7, 142.7, 141.1, 138.5, 137.6, 133.8, 132.5, 131.5, 128.9, 128.7, 128.2, 128.1, 127.5, 126.7, 126.5, 125.7, 121.7, 120.0, 119.5, 111.0, 109.5, 50.2, 47.4, 29.9, 27.6; **IR** (HFIP, cm^{-1}): 1600, 1572, 1488, 1376, 1284, 1220, 1176, 1168, 1148, 1072, 996, 884, 808, 760, 724, 688; **UV-Vis** (CH_2Cl_2): λ_{max} = 608 nm; **HRMS-QToF-ESI** (*m/z*): $[M + H]^+$ calcd. for $C_{29}H_{23}BrF_3N_4O_3S^+$, 643.0621; found, 643.0655.

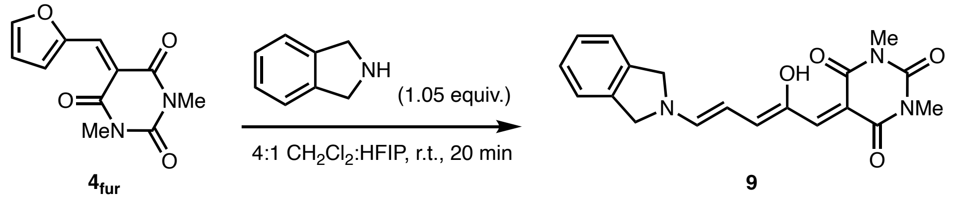

Supplementary Fig. 19. **Synthesis of hydroxy DASA 9.**

5-((2*Z*,4*E*)-2-hydroxy-5-(isoindolin-2-yl)penta-2,4-dien-1-ylidene)-1,3-dimethylpyrimidine-2,4,6(1*H*,3*H*,5*H*)-trione (9). In a 4 mL dram vial charged with a magnetic stir bar, isoindoline (59 µL, 0.525 mmol) was added to 4:1 CH_2Cl_2:HFIP (1 mL). Activated furan **4fur** (117 mg, 0.5 mmol) was subsequently added and the mixture was stirred at room temperature. An immediate color change to purple was observed. After 20 minutes, the mixture was allowed to sit at 0 ºC for 15 minutes. The solid precipitant was vacuum filtered and washed with hexanes. The crude solid was purified by flash chromatography (99.5:0.5 → 98:2 CH_2Cl_2:MeOH) to provide DASA **9** (81 mg, 46% yield) as a purple solid. **R*f***: 0.28 (95:5 CH_2Cl_2:MeOH); **^{1}H NMR** (600 MHz, 3:2 $CDCl_3$:HFIP): δ = 12.92 (s, 1H), 7.92 – 7.90 (d, *J* = 11.5 Hz, 1H), 7.45 – 7.41 (m, 2H), 7.38 – 7.33 (m, 2H), 7.14 – 7.12 (d, *J* = 13.4 Hz, 1H), 6.74 (s, 1H), 6.38 – 6.34 (t, *J* = 12.4 Hz, 1H), 5.21 (s, 2H), 5.04 (s, 2H), 3.30 (s, 6H); **^{13}C NMR** (150 MHz, 3:2 $CDCl_3$:HFIP): δ = 206.2, 165.3, 160.8, 156.5, 153.5, 152.9, 149.9, 145.8, 140.5, 133.4, 132.9, 130.8, 129.6, 129.4, 123.1, 60.0, 55.9, 28.5, 28.0; **IR** (HFIP, cm^{-1}): 2960, 2928, 2876, 2796, 2368, 2332, 2284, 2264, 2184, 2156, 1972, 1848, 1828, 1792, 1772, 1736, 1708, 1684, 1648, 1540, 1520, 1492, 1476, 1456, 1396, 1376, 1204, 1144, 1076, 760, 696; **UV-Vis** (CH_2Cl_2): λ_{max} 573 nm; **HRMS-QToF-ESI** (*m/z*): $[M + Na]^+$ calcd. for $C_{19}H_{19}N_3O_4Na^+$, 376.1268; found, 376.1293.

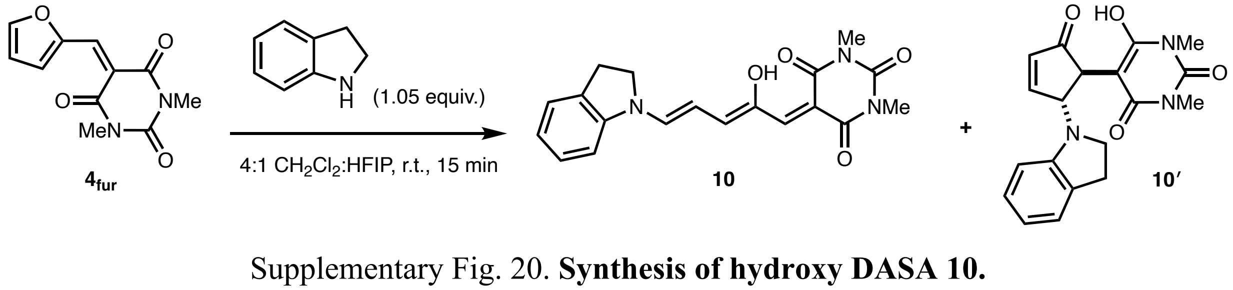

Supplementary Fig. 20. **Synthesis of hydroxy DASA 10.**

5-((2*Z*,4*E*)-2-hydroxy-5-(indolin-1-yl)penta-2,4-dien-1-ylidene)-1,3-dimethylpyrimidine-2,4,6(1*H*,3*H*,5*H*)-trione (10).). In a 4 mL dram vial charged with a magnetic stir bar, indoline

(59 µL, 0.525 mmol) was added to 4:1 CH_2Cl_2:HFIP (1 mL). Activated furan **4_{fur}** (117 mg, 0.5 mmol) was subsequently added and the mixture was stirred at room temperature. An immediate color change to blue was observed. After 15 minutes, the mixture was concentrated under reduced pressure, redissolved in a minimal amount of THF, and added dropwise to vigorously stirring cold Et_2O (50 mL). The solid precipitant was vacuum filtered to provide a 1:2 open(**10**):closed(**10′**) isomeric mixture of DASA **10** (125 mg, 70% yield) as a blue solid. **^{1}H NMR** (500 MHz, $CDCl_3$): Select shifts of closed isomer **10′**, δ = 7.69 – 7.67 (d, *J* = 6.1 Hz, 1H), 7.39 (s, 1H), 7.11 – 7.07 (m, 2H), 7.01 –6.44 – 6.42 (d, *J* = 7.0 Hz, 1H), 5.37 (b, 1H), 5.30 (d, *J* = 1.6 Hz, 2H), 4.13 – 4.09 (t, *J* = 7.9 Hz, 1H), 3.36 – 3.30 (m, 9H), 3.06 – 3.00 (m, 1H). Spectral data matches previously reported values.[10]

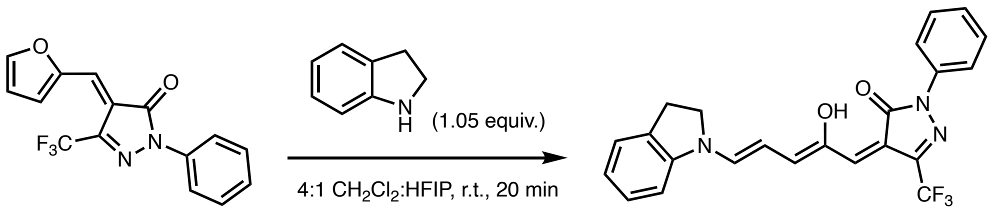

Supplementary Fig. 21. **Synthesis of hydroxy DASA 11.**

(*Z*)-4-((2*Z*,4*E*)-2-hydroxy-5-(indolin-1-yl)penta-2,4-dien-1-ylidene)-2-phenyl-5-(trifluoromethyl)-2,4-dihydro-3*H*-pyrazol-3-one (11). In a 4 mL dram vial charged with a magnetic stir bar, indoline (24 µL, 0.21 mmol) was added to 4:1 CH_2Cl_2:HFIP (0.5 mL). Activated furan **S3** (60 mg, 0.2 mmol) was subsequently added and the mixture was stirred at room temperature. An immediate color change to blue was observed. After 20 minutes, the mixture was concentrated under reduced pressure, redissolved in a minimal amount of THF, and added dropwise to vigorously stirring cold Et_2O (50 mL). The solid precipitant was vacuum filtered and purified by flash chromatography (99.5:0.5 → 98:2 CH_2Cl_2:MeOH) to provide DASA **11** (58 mg, 68% yield) as a green solid. **^{1}H NMR** (500 MHz, $CDCl_3$): δ = 13.02 (s, 1H), 7.93 – 7.91 (d, *J* = 8.0 Hz, 2H), 7.74 – 7.72 (d, *J* = 12.6 Hz, 1H), 7.42 – 7.40 (t, *J* = 7.8 Hz, 2H), 7.30 – 7. 21 (m, 3H), 7.07 – 7.04 (m, 2H), 6.72 – 6.70 (d, *J* = 12.3 Hz, 1H), 6.63 (s, 1H), 6.33 – 6.28 (t, *J* = 12.4 Hz, 1H), 4.18 – 4.16 (t, *J* = 7.8 Hz, 2H), 3.35 – 3.33 (t, *J* = 7.0 Hz, 2H). Spectral data matches previously reported values.[11]

Calculation for relative λ_{max} changes

$$\left(\frac{\Delta(\lambda_{max})_{lg} - \Delta(\lambda_{max})_{sm}}{\Delta(\lambda_{max})_{sm}}\right) \times 100\% = \text{ relative } \lambda_{max} \text{ change}$$

<u>Example</u>

**DASA**	λ_{max} **(nm)**
**7**	531
**6e**	578
**9**	573
**10**	615

$\Delta(\lambda_{max})_{lg} = (\lambda_{max})_{\mathbf{6e}} - (\lambda_{max})_{\mathbf{7}}$ = 578 nm - 531 nm = 47 nm

$\Delta(\lambda_{max})_{sm} = (\lambda_{max})_{\mathbf{10}} - (\lambda_{max})_{\mathbf{9}}$ = 615 nm - 573 nm = 42 nm

$$\left(\frac{47 \text{ nm} - 42 \text{ nm}}{42 \text{ nm}}\right) \times 100\% = \ 12\%$$

C. UV-vis absorbance measurements

C.1. Molar absorption coefficient and solvatochromic analysis

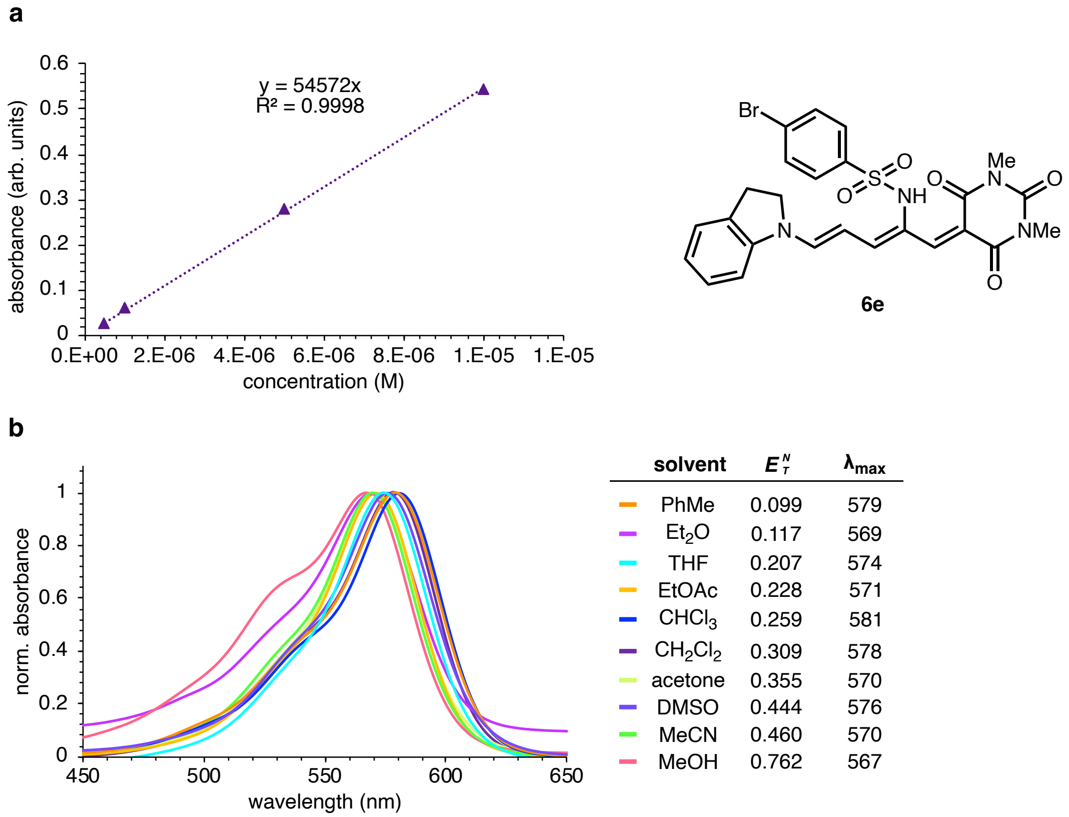

solvent	E_T^N	λ_{max}
PhMe	0.099	579
Et_2O	0.117	569
THF	0.207	574
EtOAc	0.228	571
$CHCl_3$	0.259	581
CH_2Cl_2	0.309	578
acetone	0.355	570
DMSO	0.444	576
MeCN	0.460	570
MeOH	0.762	567

Supplementary Fig. 22. **Amino DASA 6e photophysical characterization. a** UV-visible absorption measurements of **6e** were taken at 10, 5, 1, and 0.5 μM in CH_2Cl_2. Absorbance measurements were normalized and the λ_{max} was plotted against concentration (M). The slope of linear extrapolation, 54572 is the molar absorption coefficient for **6e** (ε = 5.5x10^4 M^{-1}cm^{-1}). **b** Solvatochromic analysis was performed by obtaining the UV-visible absorption measurements of **6e** in PhMe, Et_2O, THF, EtOAc, $CHCl_3$, CH_2Cl_2, acetone, DMSO, MeCN, and MeOH. The λ_{max} of absorbance in each solvent was plotted against the respective polarity value (E_T^N) using the Dimroth-Reichardt parameters (See Supplementary Fig. 8).

a

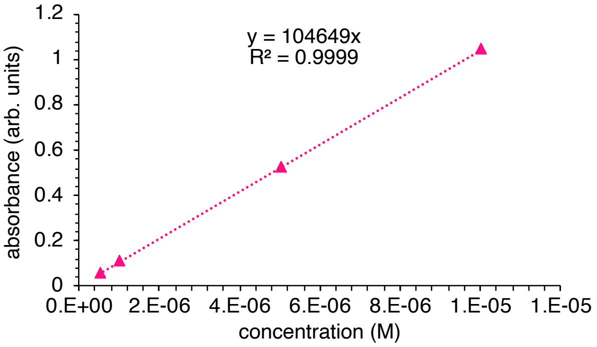

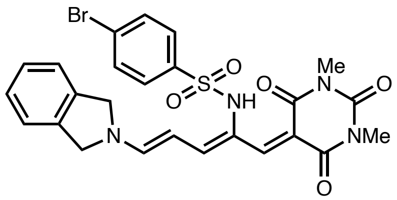

7

b

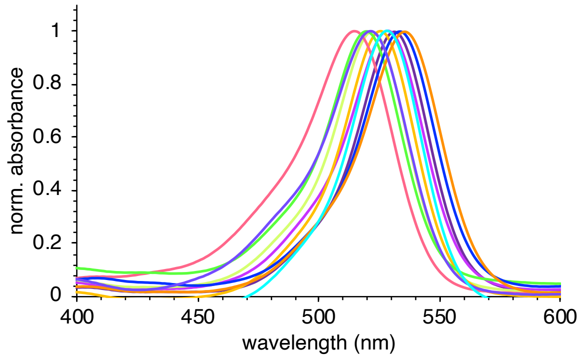

solvent	E_T^N	λ_{max}
PhMe	0.099	535
Et_2O	0.117	528
THF	0.207	528
EtOAc	0.228	525
$CHCl_3$	0.259	533
CH_2Cl_2	0.309	531
acetone	0.355	522
DMSO	0.444	521
MeCN	0.460	519
MeOH	0.762	515

Supplementary Fig. 23. **Amino DASA 7 photophysical characterization. a** UV-visible absorption measurements of **7** were taken at 10, 5, 1, and 0.5 μM in CH_2Cl_2. The λ_{max} was plotted against concentration (M). The slope of linear extrapolation, 104649 is the molar absorption coefficient for **7** ($\varepsilon = 1.0 \times 10^5$ $M^{-1}cm^{-1}$). **b** Solvatochromic analysis was performed by obtaining the UV-visible absorption measurements of **7** in PhMe, Et_2O, THF, EtOAc, $CHCl_3$, CH_2Cl_2, acetone, DMSO, MeCN, and MeOH. The λ_{max} of absorbance in each solvent was plotted against the respective polarity value (E_T^N) using the Dimroth-Reichardt parameters (See Supplementary Fig. 8).

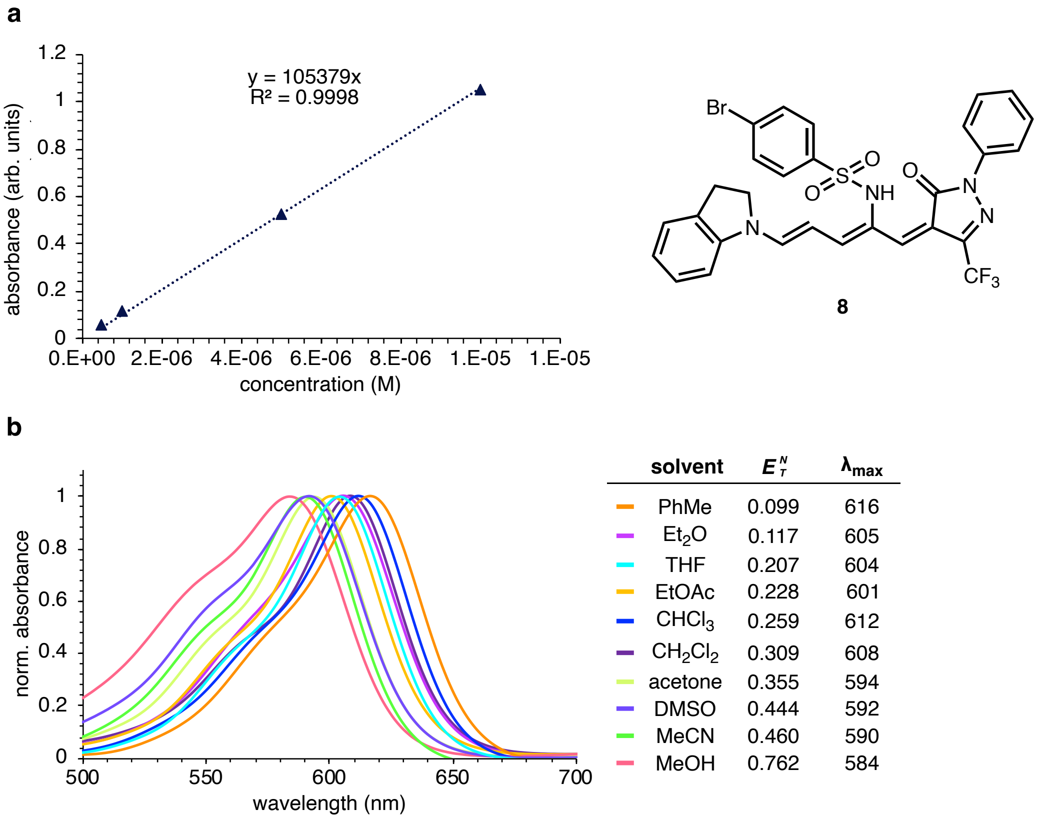

Supplementary Fig. 24. **Amino DASA 8 photophysical characterization. a** UV-visible absorption measurements of **8** were taken at 10, 5, 1, and 0.5 µM in CH_2Cl_2. The λ_{max} was plotted against concentration (M). The slope of linear extrapolation, 105379 is the molar absorption coefficient for **8** ($\varepsilon = 1.1 \times 10^5$ $M^{-1}cm^{-1}$). **b** Solvatochromic analysis was performed by obtaining the UV-visible absorption measurements of **8** in PhMe, Et_2O, THF, EtOAc, $CHCl_3$, CH_2Cl_2, acetone, DMSO, MeCN, and MeOH. The λ_{max} of absorbance in each solvent was plotted against the respective polarity value (E_T^N) using the Dimroth-Reichardt parameters (See Supplementary Fig. 8).

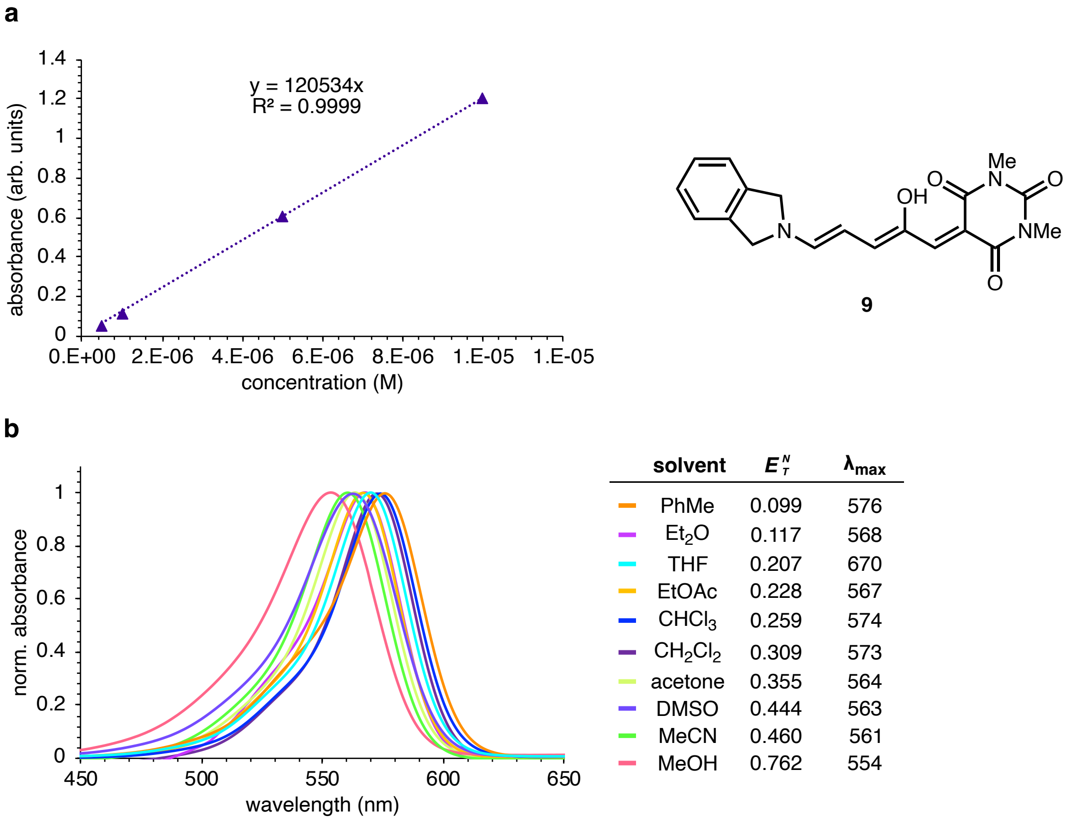

Supplementary Fig. 25. **Hydroxy DASA 9 photophysical characterization. a** UV-visible absorption measurements of **9** were taken at 10, 5, 1, and 0.5 μM in CH_2Cl_2. The λ_{max} was plotted against concentration (M). The slope of linear extrapolation, 120534 is the molar absorption coefficient for **9** ($\varepsilon = 1.2 \times 10^5$ $M^{-1}cm^{-1}$). **b** Solvatochromic analysis was performed by obtaining the UV-visible absorption measurements of **9** in PhMe, Et_2O, THF, EtOAc, $CHCl_3$, CH_2Cl_2, acetone, DMSO, MeCN, and MeOH. The λ_{max} of absorbance in each solvent was plotted against the respective polarity value (E_T^N) using the Dimroth-Reichardt parameters (See Supplementary Fig. 8).

a

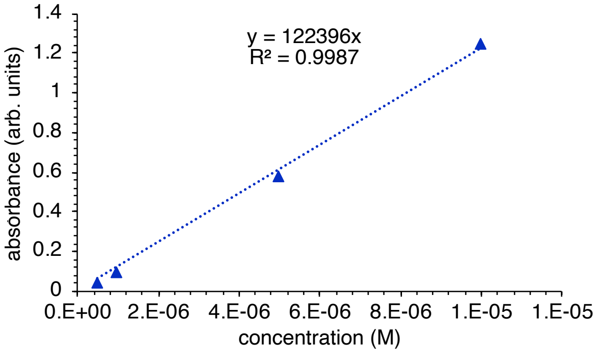

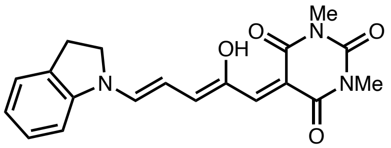

10

b

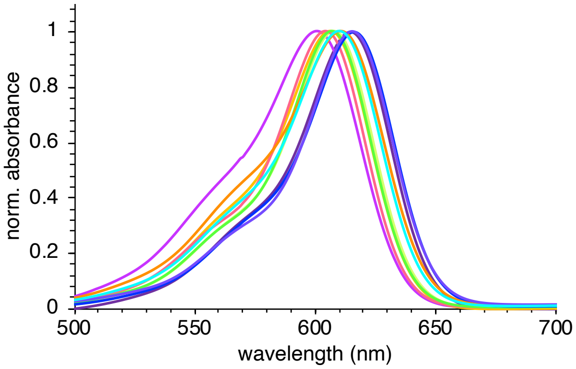

solvent	E_T^N	λ_{max}
PhMe	0.099	611
Et_2O	0.117	601
THF	0.207	610
EtOAc	0.228	606
$CHCl_3$	0.259	616
CH_2Cl_2	0.309	615
acetone	0.355	608
DMSO	0.444	615
MeCN	0.460	607
MeOH	0.762	604

Supplementary Fig. 26. **Hydroxy DASA 10 photophysical characterization. a** UV-visible absorption measurements of **10** were taken at 10, 5, 1, and 0.5 µM in CH_2Cl_2. The λ_{max} was plotted against concentration (M). The slope of linear extrapolation, 122396 is the molar absorption coefficient for **10** ($\varepsilon = 1.2 \times 10^5$ $M^{-1}cm^{-1}$). **b** Solvatochromic analysis was performed by obtaining the UV-visible absorption measurements of **10** in PhMe, Et_2O, THF, EtOAc, $CHCl_3$, CH_2Cl_2, acetone, DMSO, MeCN, and MeOH. The λ_{max} of absorbance in each solvent was plotted against the respective polarity value (E_T^N) using the Dimroth-Reichardt parameters (See Supplementary Fig. 8).

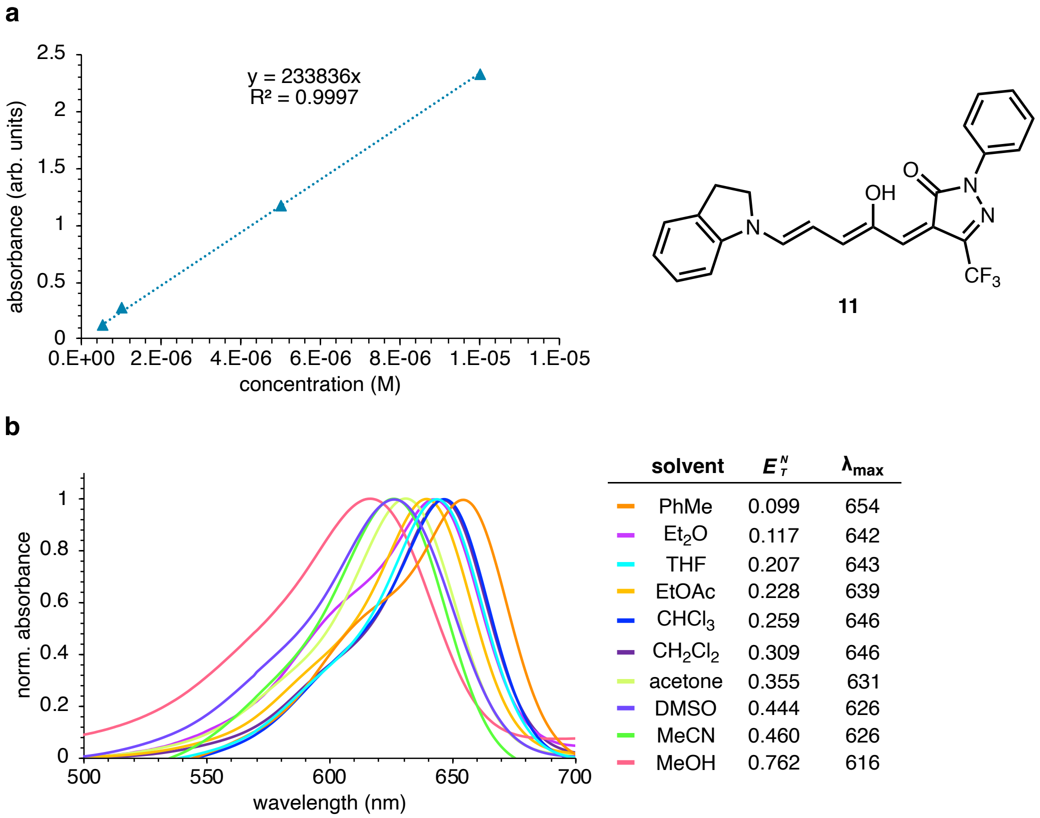

Supplementary Fig. 27. **Hydroxy DASA 11 photophysical characterization. a** UV-visible absorption measurements of **11** were taken at 10, 5, 1, and 0.5 μM in CH_2Cl_2. The λ_{max} was plotted against concentration (M). The slope of linear extrapolation, 233836 is the molar absorption coefficient for **11** ($\varepsilon = 2.3\text{x}10^5$ $M^{-1}cm^{-1}$). **b** Solvatochromic analysis was performed by obtaining the UV-visible absorption measurements of **11** in PhMe, Et_2O, THF, EtOAc, $CHCl_3$, CH_2Cl_2, acetone, DMSO, MeCN, and MeOH. The λ_{max} of absorbance in each solvent was plotted against the respective polarity value (E_T^N) using the Dimroth-Reichardt parameters (See Supplementary Fig. 8).

C.2. Solvatochromic Slope Comparison

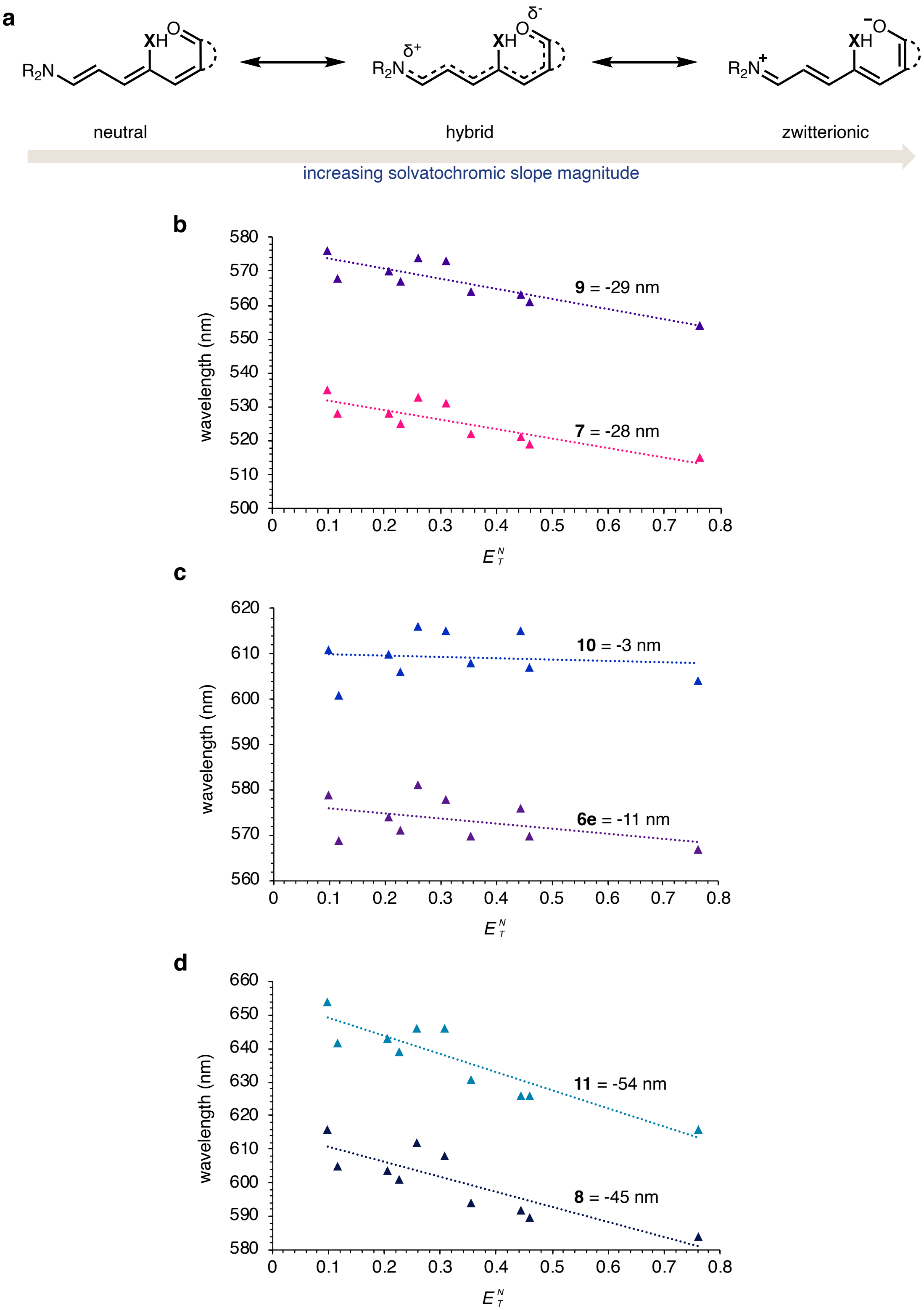

Supplementary Fig. 28. **Solvatochromic slope comparison. a** Charge distribution visual of linear DASA molecule. Greater zwitterionic character is correlated to absorbance sensitivity of solvent

polarity. Using the Dimroth-Reichardt solvent polarity parameters (Supplementary Figs. 2–7), absorbance values were plotted to provide a slope value indicative of charge distribution. Heteroatom comparison across the **b** 1st, **c** 2nd, and **d** 3rd generation DASA molecules were made.

C.3. Absorbance irradiation experiments

UV-visible absorption of 10 μM under irradiation using a broadband visible light source were measured at time points across extended periods. All absorbance values were normalized using λ_{max} of time = 0 min as 1. Following complete consumption of λ_{max}, thermal reversion of closed isomer back to open DASA was measured by respective λ_{max} measurements at 5 second intervals in the absence of irradiation for a period of 1 hour. If λ_{max} consumption was not observed, thermal reversion measurements were not performed. Due to hydroxy DASA **11**'s rapid switching rates, accurate measurements could not be obtained. See Supplementary Figs. 15–17 for *in situ* irradiation switching studies.

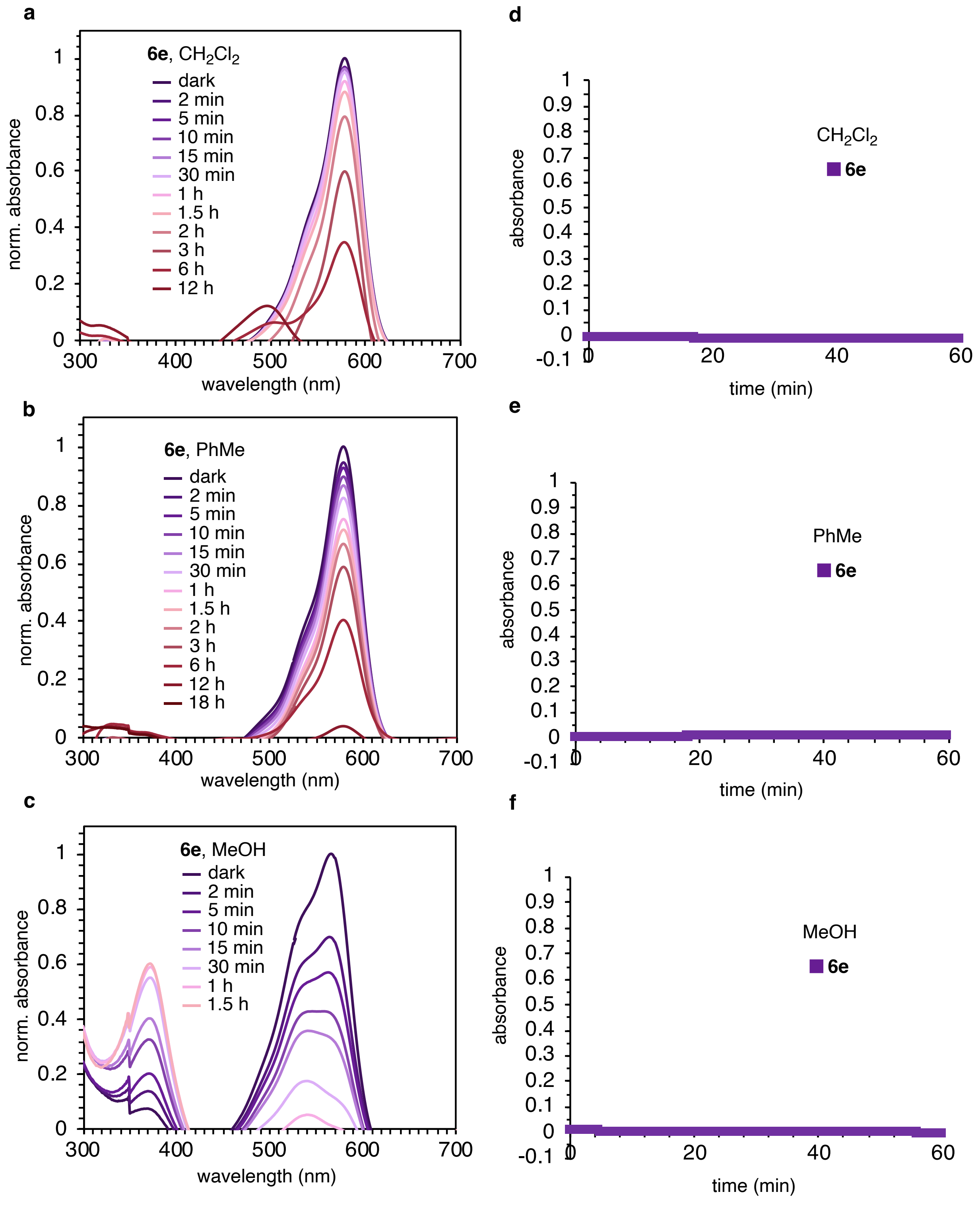

Supplementary Fig. 29. **Absorbance measurements of 6e during and after irradiation.** Irradiation absorbance measurements in **a** CH_2Cl_2, **b** PhMe, and **c** MeOH. Following consumption of **6e** λ_{max} in the respective solvents (See Supplementary Fig. 2), thermal recovery of open DASA was measured in **d** CH_2Cl_2, **e** PhMe, and **f** MeOH.

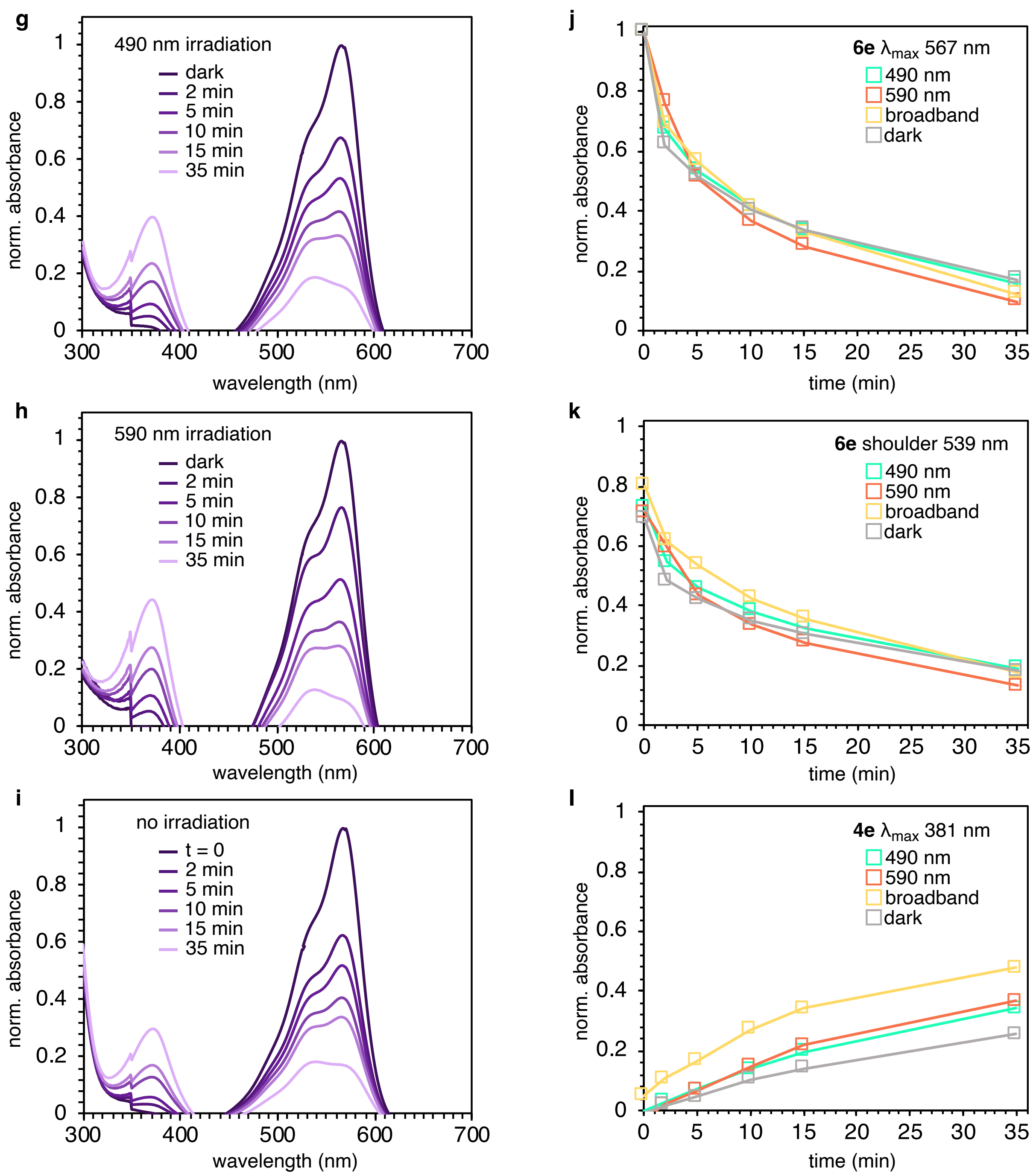

Supplementary Fig. 30. **Irradiation of 6e with various light sources in methanol.** Absorbance decreases measurements using **g** 490 nm, **h** 590 nm, and **i** no light. Absorbance comparison plots of **j 6e** λ_{max} = 567 nm, **k 6e** $\lambda_{shoulder}$ = 539 nm, and **l 4e** λ_{max} = 381 nm upon irradiation with various light sources. All values are normalized to their respective **6e** λ_{max} = 567 nm. For light source information refer to the Materials and Methods Section.

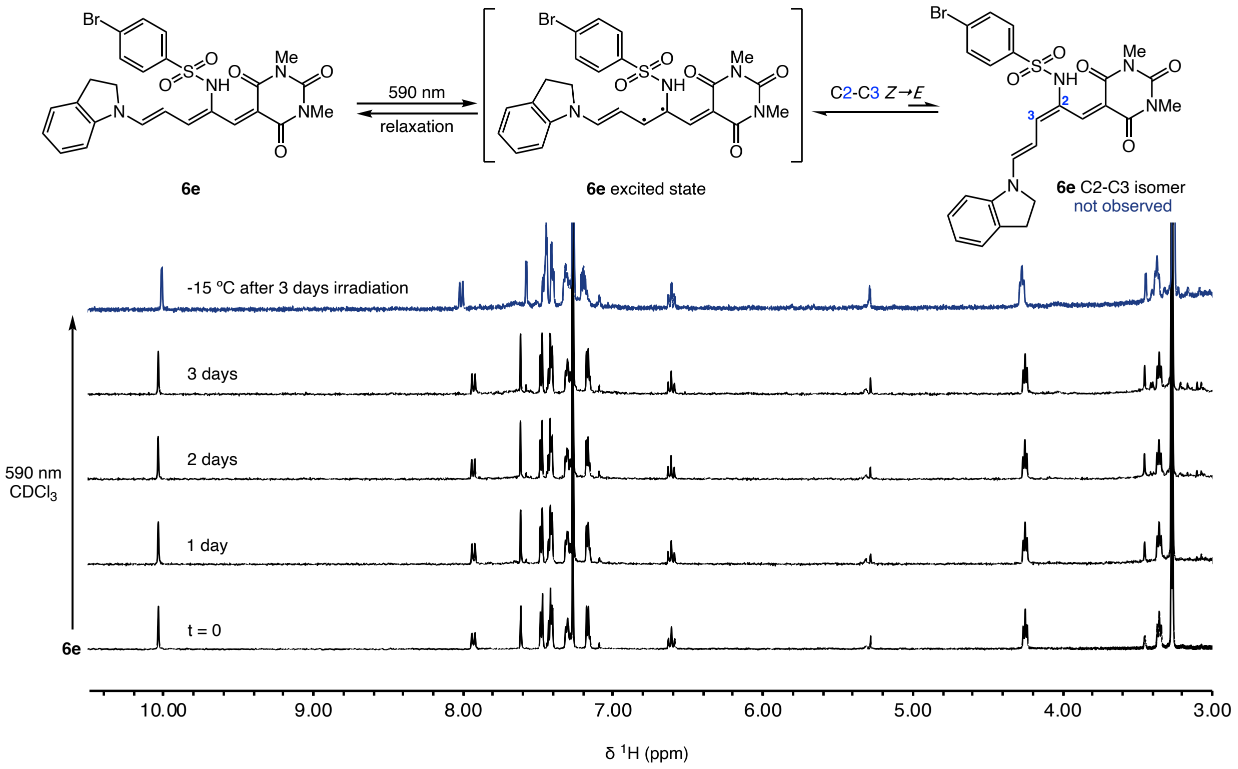

Supplementary Fig. 31. **NMR in situ irradiation of 6e with narrow band 590 nm LED in $CDCl_3$.** No closed isomer was observed upon 72 hours of irradiation. No C3–C4 *cis*→*trans* isomerization was observed at –15 °C. For light source information refer to the Materials and Methods Section.

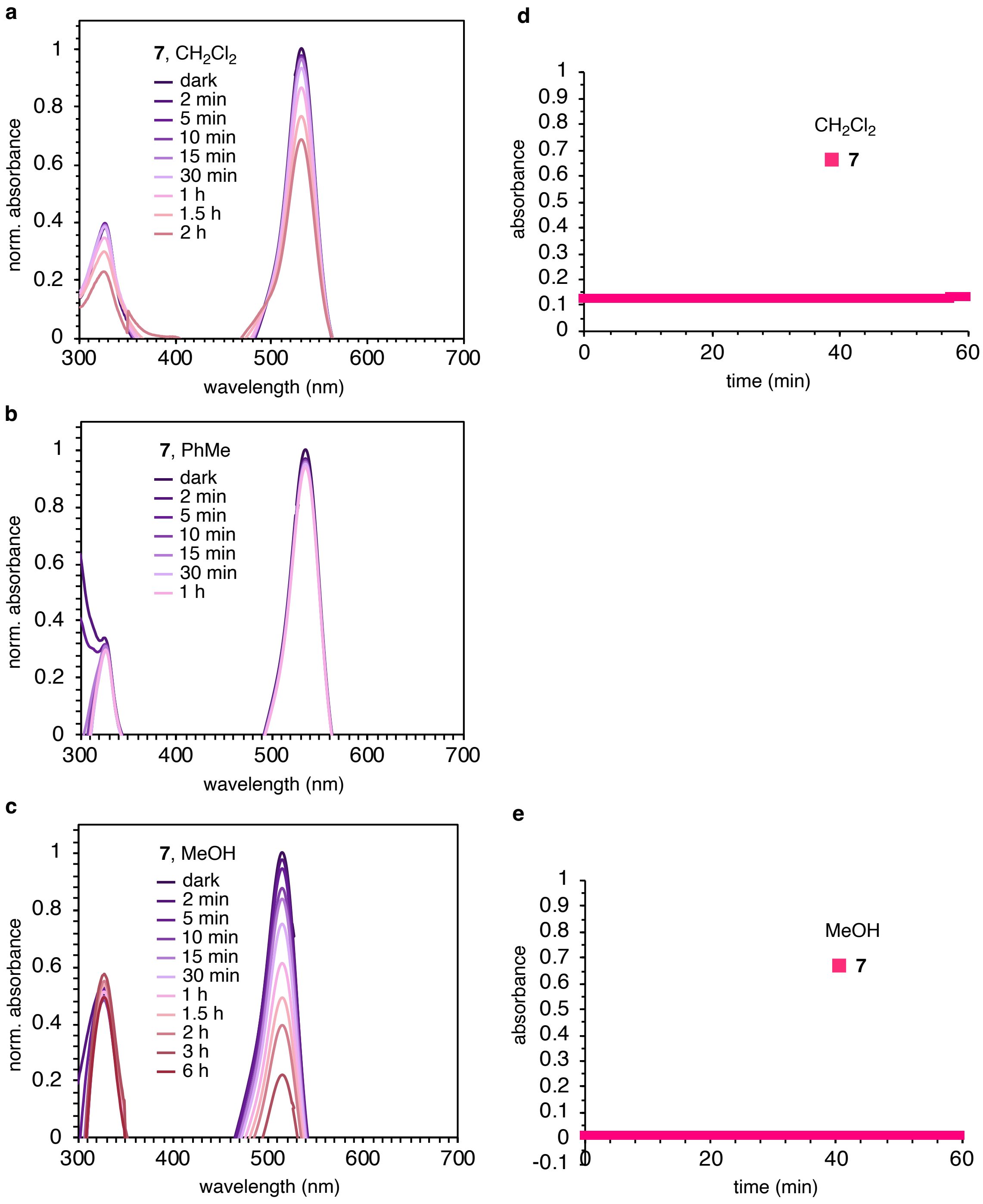

Supplementary Fig. 32. **Absorbance measurements of 7 during and after irradiation.** Irradiation absorbance measurements in **a** CH_2Cl_2, **b** PhMe, and **c** MeOH. Following consumption of **7** λ_{max} in the respective solvents (See Supplementary Fig. 3), thermal recovery of open DASA was measured in **d** CH_2Cl_2 and **e** MeOH.

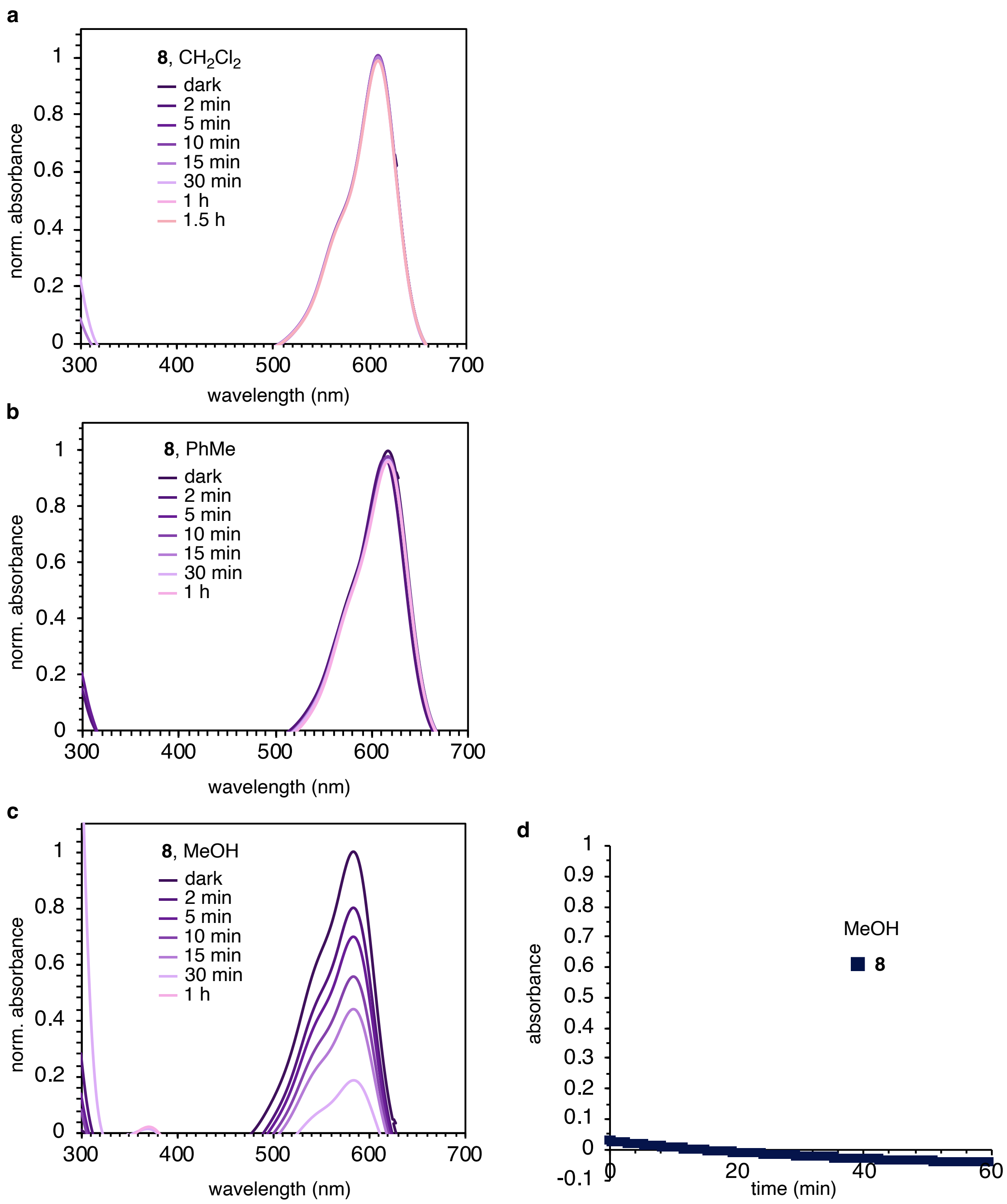

Supplementary Fig. 33. **Absorbance measurements of 8 during and after irradiation.** Irradiation absorbance measurements in **a** CH_2Cl_2, **b** PhMe, and **c** MeOH. Following consumption of **8** λ_{max} in the respective solvents (See Supplementary Fig. 4), thermal recovery of open DASA was measured in **d** MeOH.

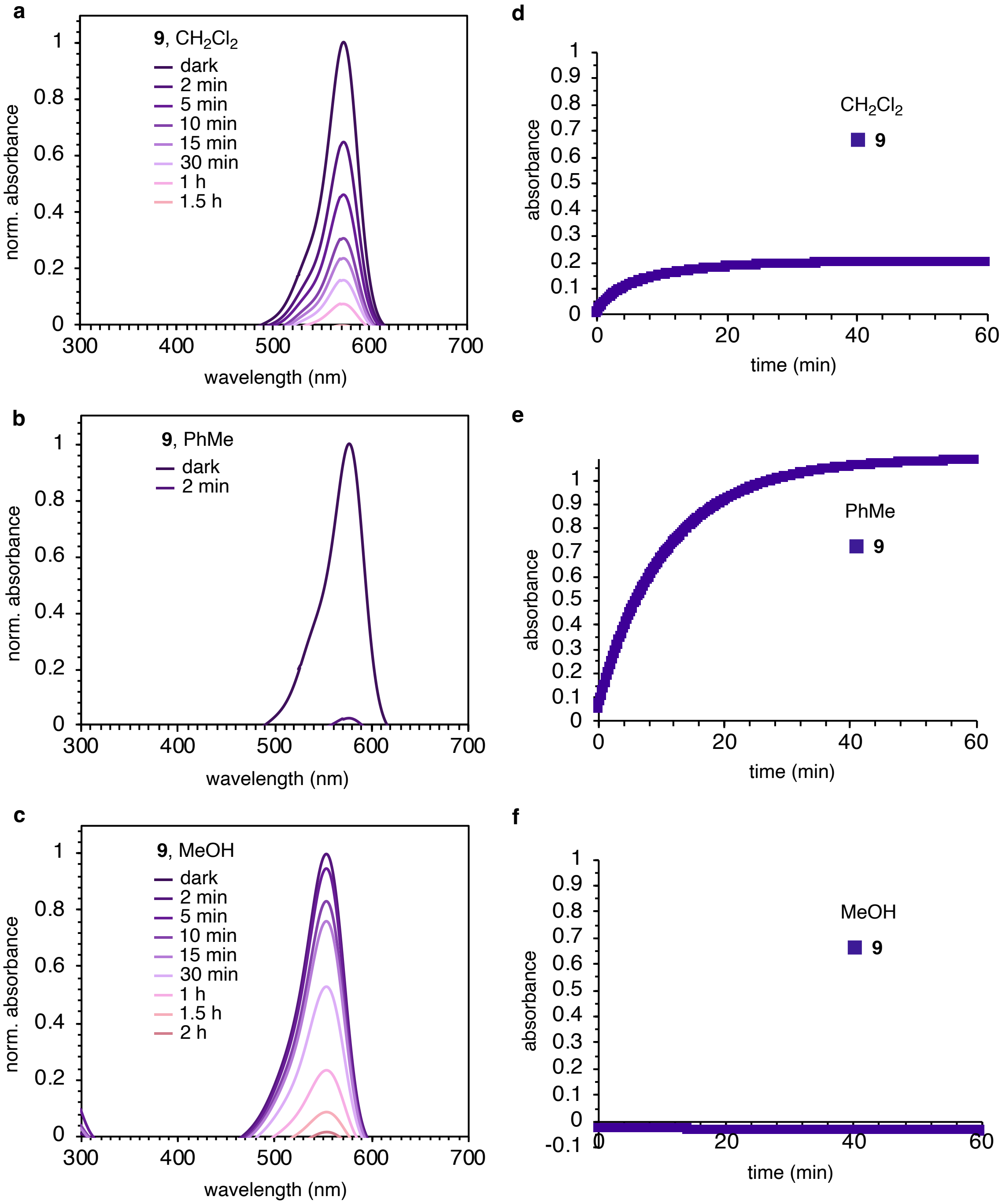

Supplementary Fig. 34. **Absorbance measurements of 9 during and after irradiation.** Irradiation absorbance measurements in **a** CH_2Cl_2, **b** PhMe, and **c** MeOH. Following consumption of **9** λ_{max} in the respective solvents (See Supplementary Fig. 5), thermal recovery of open DASA was measured in **d** CH_2Cl_2, **e** PhMe, and **f** MeOH.

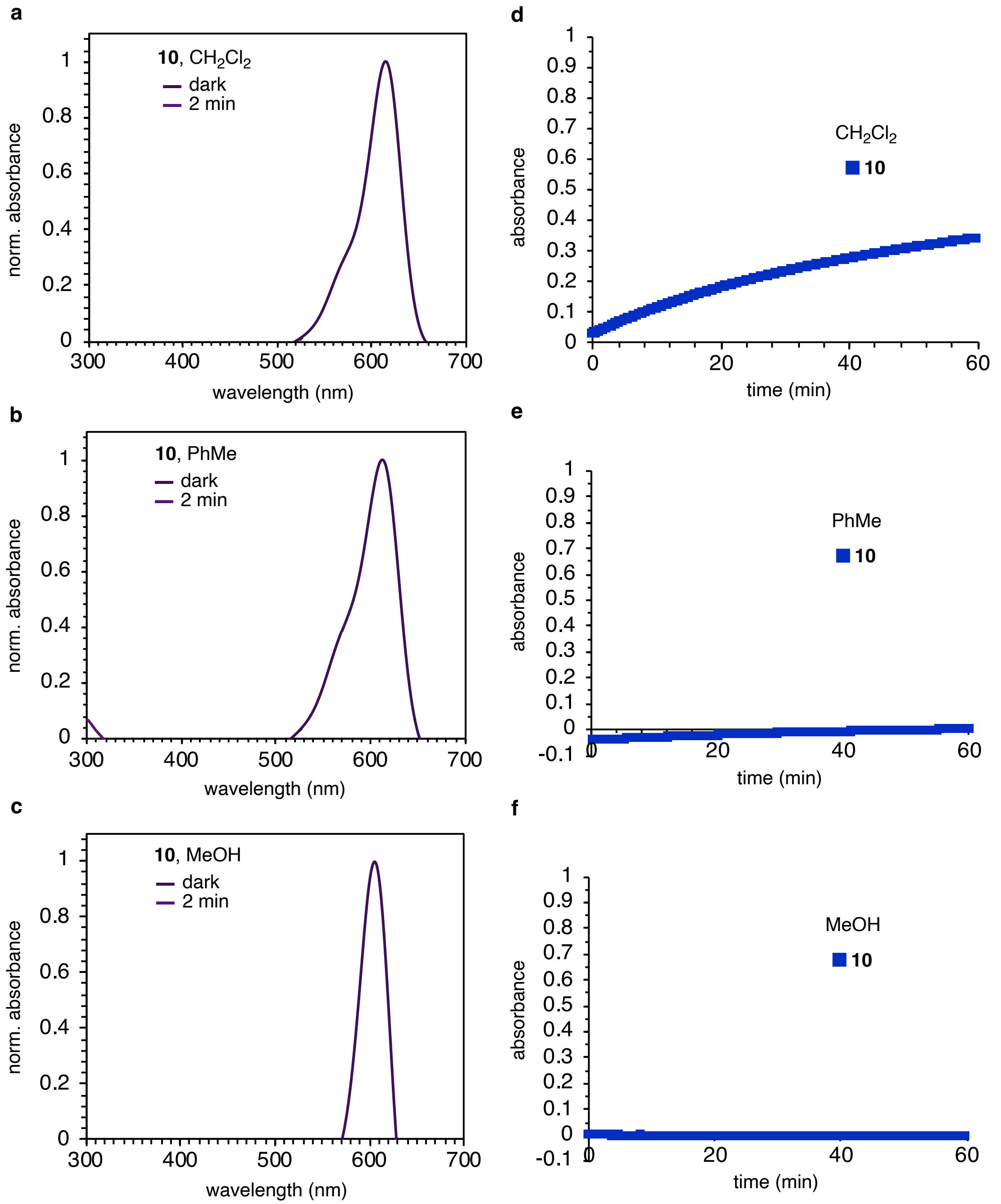

Supplementary Fig. 35. **Absorbance measurements of 10 during and after irradiation.** Irradiation absorbance measurements in **a** CH_2Cl_2, **b** PhMe, and **c** MeOH. Following consumption of **10** λ_{max} in the respective solvents (See Supplementary Fig. 6), thermal recovery of open DASA was measured in **d** CH_2Cl_2, **e** PhMe, and **f** MeOH.

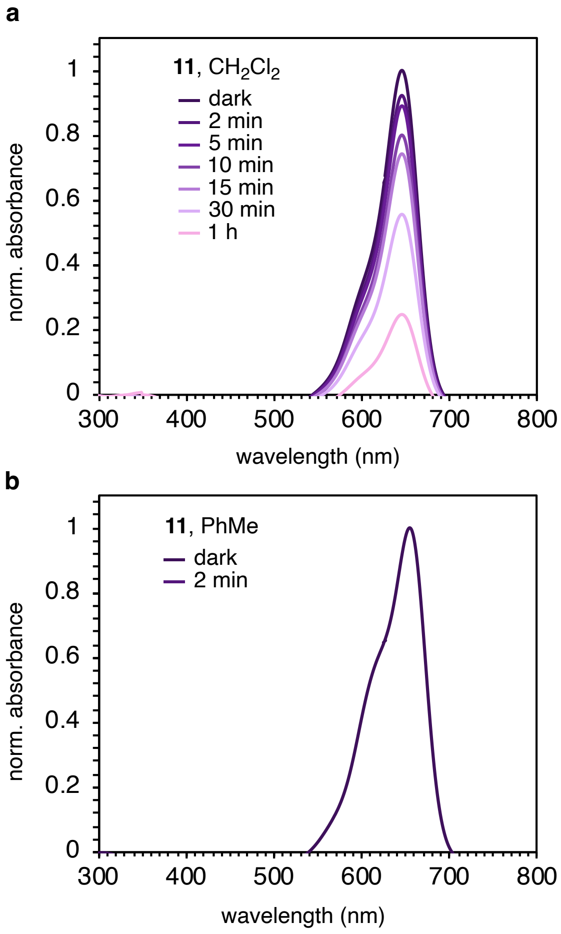

Supplementary Fig. 36. **Absorbance measurements of 11 during irradiation.** Irradiation absorbance measurements in **a** CH_2Cl_2 and **b** PhMe. Due to rapid photoswitching, accurate thermal reversion measurements could not be performed. See Supplementary Figs. 15–17 for *in situ* irradiation switching.

C.4. Photoswitching studies

Photoswitching studies with *in situ* absorbance measurements obtained at 5 sec intervals over 40 min unless otherwise noted.* Measurements were taken throughout a 2 min dark period, a 10 min irradiation period with a broadband visible light source, and a subsequent 28 min dark period. All measurements were taken at the DASA's respective λ_{max} in the given solvent. All concentrations were 10 µM.†

Notes

*Measurement intervals at 0.1 sec were taken for **10** in PhMe, CH_2Cl_2, and MeOH, and for **6e** in MeOH

†During irradiation a large amount of absorbance measurement noise appeared. Change of irradiation set-up and measurement parameters did not fix noise issue, but only dampened intensity. However, general absorbance changes and thermal reversion observations are consistent with other experiments (See Supplementary Figs. 9–14)

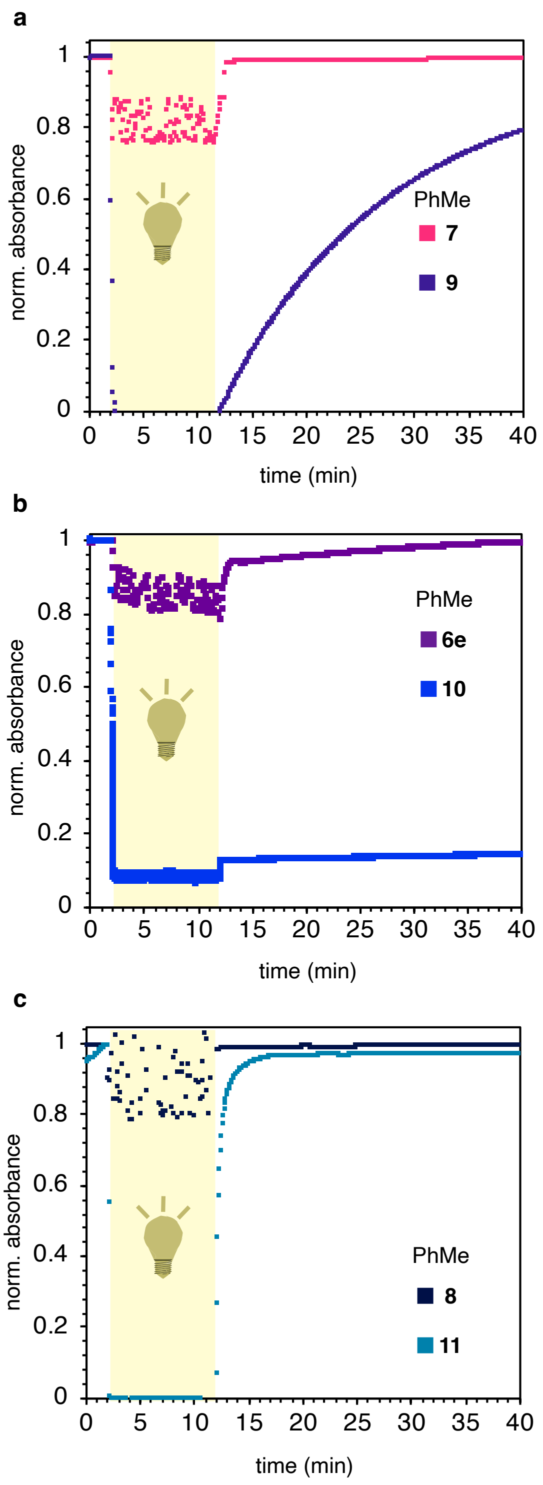

Supplementary Fig. 37. ***In situ* absorbance measurements in PhMe across a 10 min irradiation period.** Switching comparison of **a** 1st, **b** 2nd, and **c** 3rd generation DASAs in PhMe.

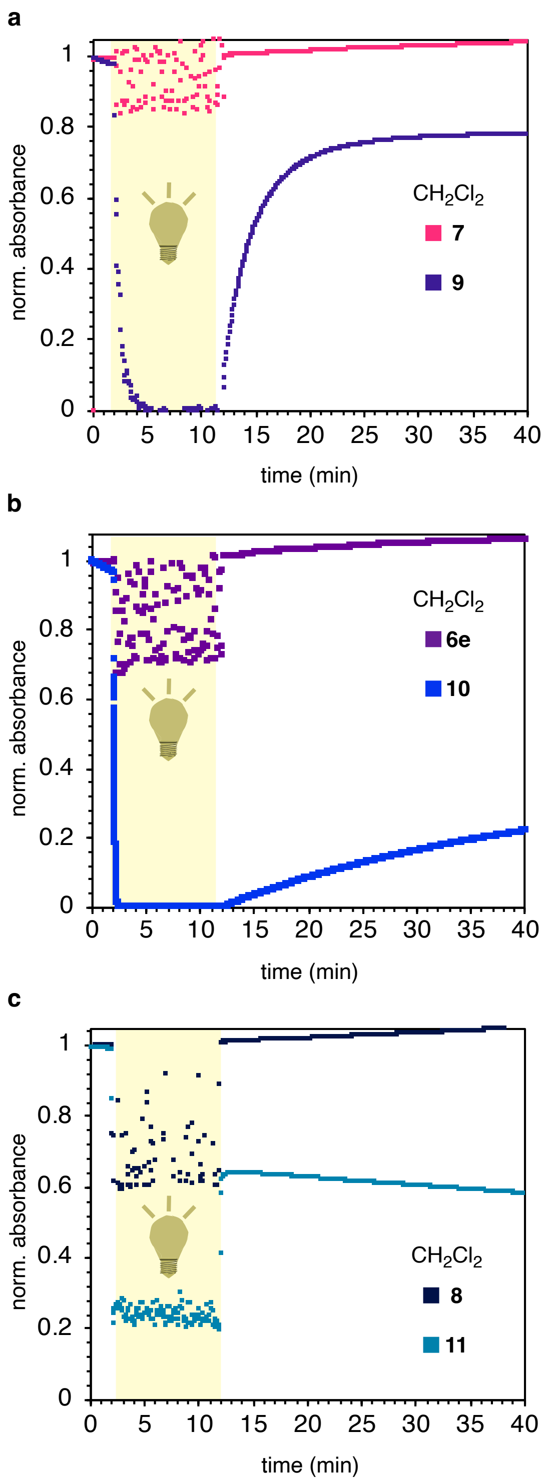

Supplementary Fig. 38. ***In situ* absorbance measurements in CH_2Cl_2 across a 10 min irradiation period.** Switching comparison of **a** 1st, **b** 2nd, and **c** 3rd generation DASAs in CH_2Cl_2.

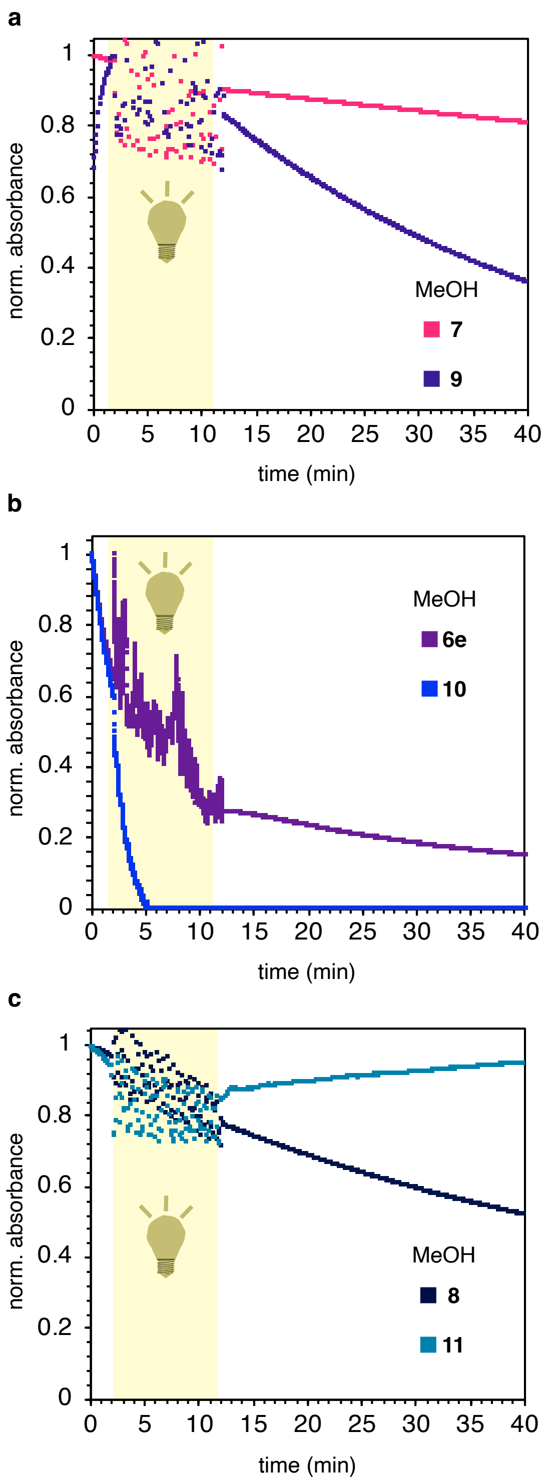

Supplementary Fig. 39. ***In situ* absorbance measurements in MeOH across a 10 min irradiation period.** Switching comparison of **a** 1st, **b** 2nd, and **c** 3rd generation DASAs in MeOH.

C.5. Thermal stability evaluation

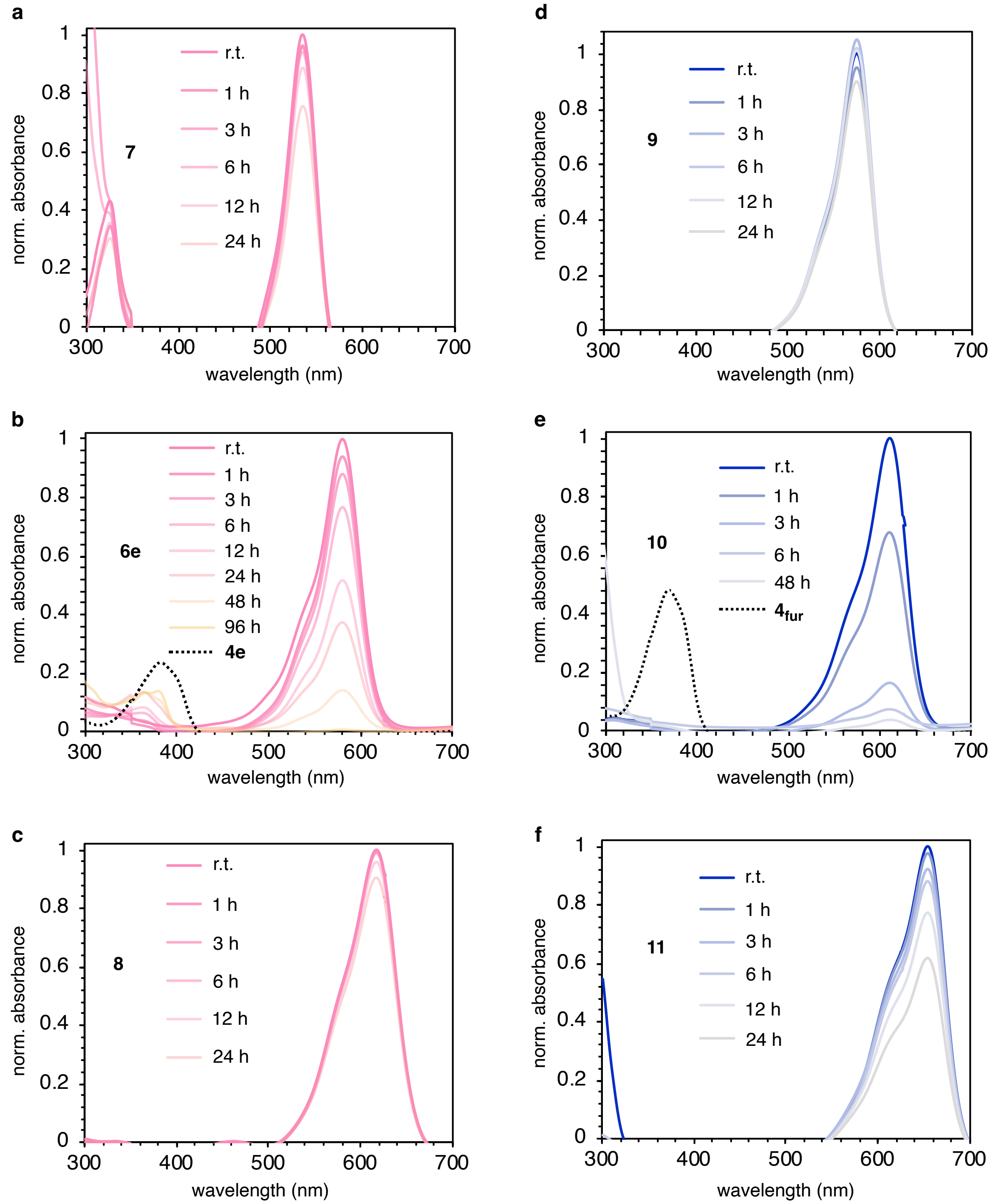

Supplementary Fig. 40. **Thermal stability evaluation of DASAs.** At 10 μM concentration in PhMe, the stability of DASAs **a 7**, **b 6e**, **c 8**, **d 9**, **e 10**, and **f 11** were evaluated by absorbance decrease during 40 ºC incubation periods in the absence of light. Measurements were taken at time

points up until complete consumption of respective λ_{max}. Absorbance measurement of 10 µM of activated pyrrole **4e** (**b**) and activated furan **4**$_{\mathbf{fur}}$ (**e**) in PhMe were plotted for comparison.

Calculation notes

In main text Figure 4f, the λ_{max} of **6e** (578 nm) and **4e** (381 nm) were plotted across the 96-hour time period as measured in Supplementary Fig. 18b. Normalization of the absorbance values were obtained by dividing by absorbance measurements by the t = 0 (initial measurement) of **6e** λ_{max} for **6e** plot and by dividing by absorbance measurements by the **4e** λ_{max} at 10 µM for **4e** plot.

<u>Example</u>

**compound**	10 µM absorbance	$t = 0$ absorbance	96 h absorbance
**6e**	0.859	0.859	0.006
**4e**	0.234	0	0.115

6e at 96 h

$$\left(\frac{0.006}{0.859}\right) \times 100\% = < 1\%$$

4e at 96 h

$$\left(\frac{0.115}{0.234}\right) \times 100\% = \ 49\%$$

D. NMR *in situ* irradiation

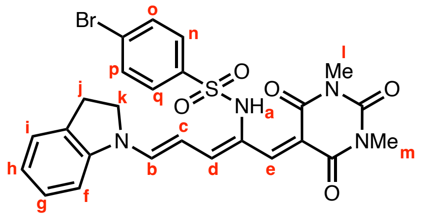

amino DASA **6e**

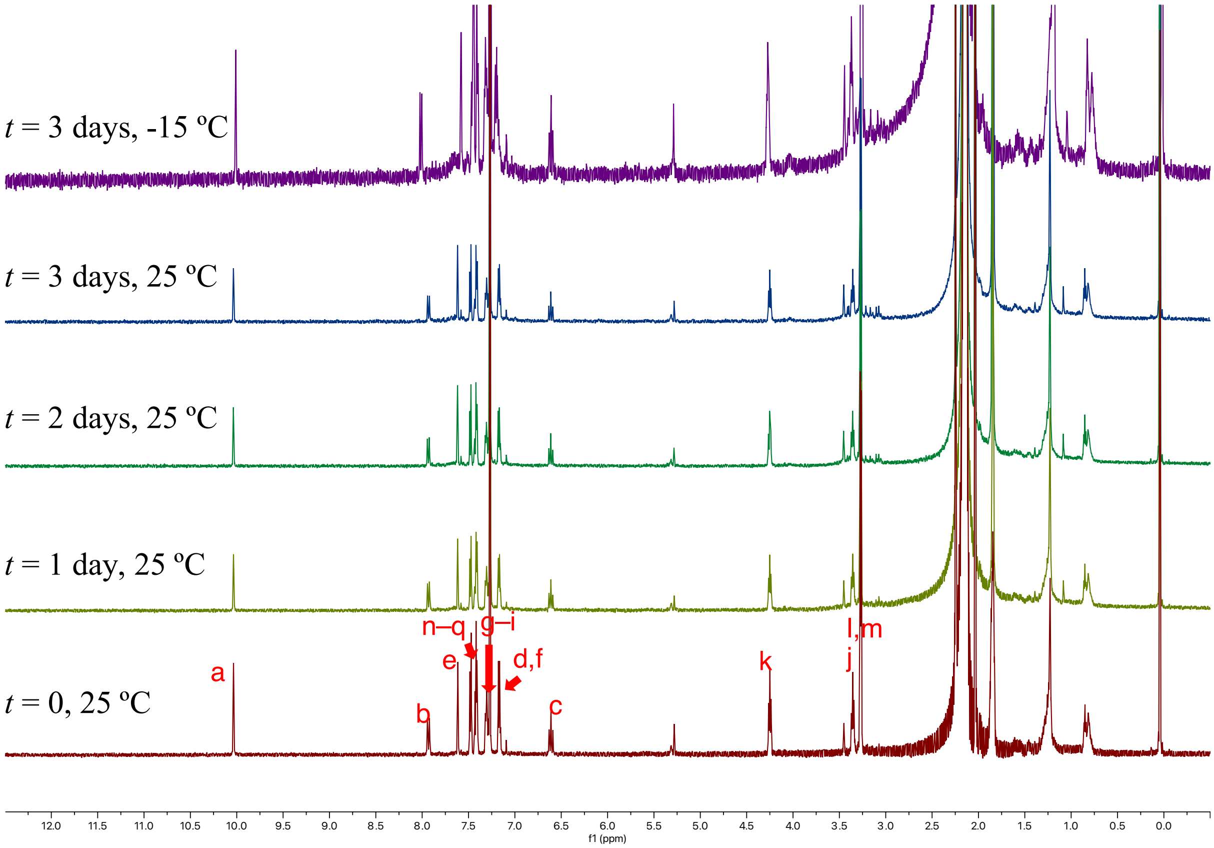

Supplementary Fig. 41. **^{1}H NMR stack of *in situ* irradiation of amino DASA 6e.** Over the period of 3 days, a 1.0 mg/mL solution of **6e** in $CDCl_3$ was irradiated as described in *Materials and Methods*. During that period, ^{1}H NMR (600 MHz) spectra were obtained at 25 ºC. After irradiating for 72 hrs, the sample was cooled to –15 ºC in the NMR and the spectra was obtained at –15 ºC. Notably, no significant spectroscopic changes were observed throughout the experiment or after cooling.

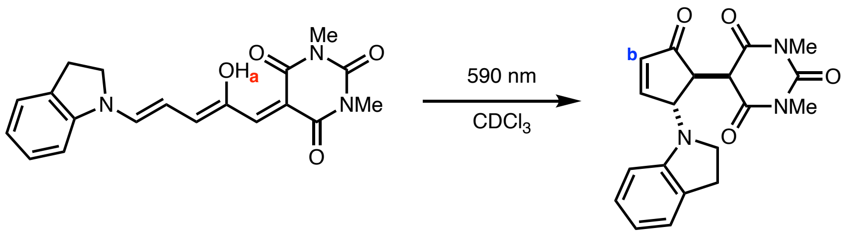

hydroxy DASA **10** and **10'**

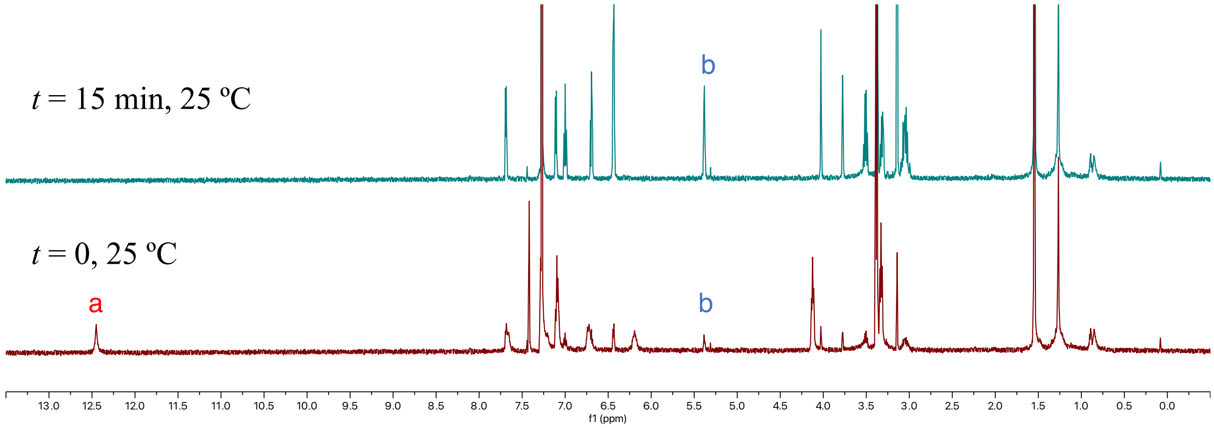

Supplementary Fig. 42. **^{1}H NMR stack of *in situ* irradiation of hydroxy DASA 10.** Over the period of 15 min, a 1.0 mg/mL solution of **10** in $CDCl_3$ was irradiated as described in *Materials and Methods*. During that period, ^{1}H NMR (600 MHz) spectra were obtained at 25 ºC. Notably, complete conversion of **10** to **10'** was observed within 15 min.

E. Hammett value analysis

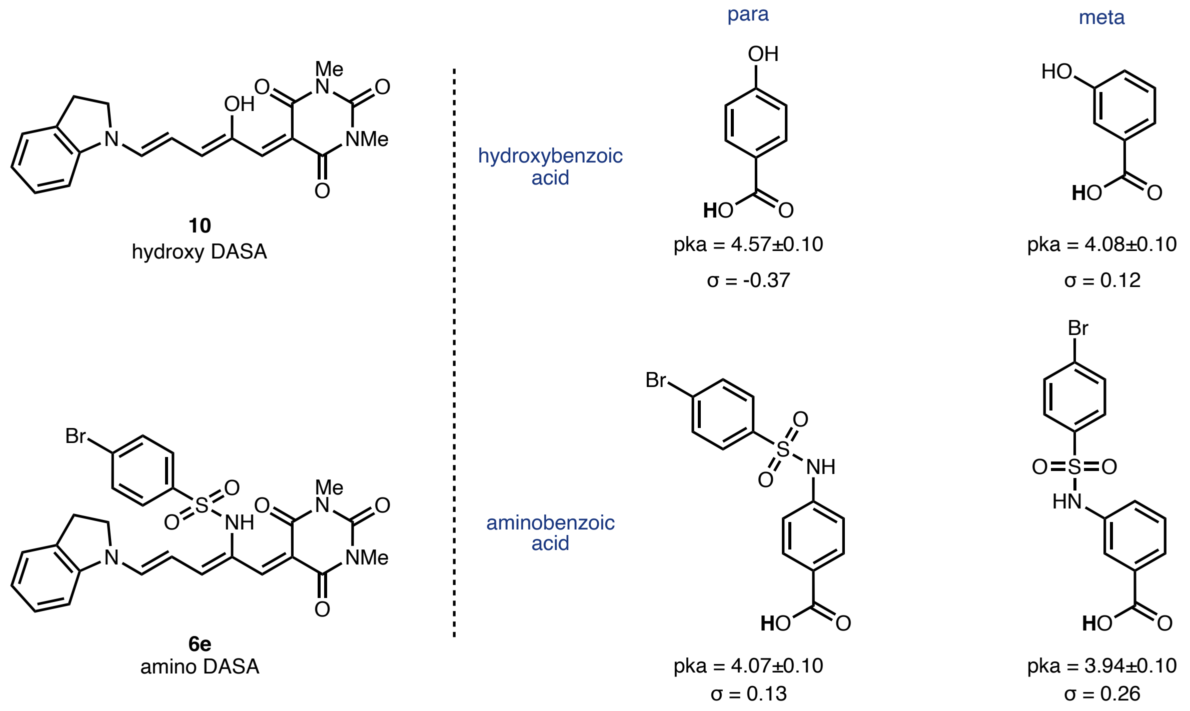

Supplementary Fig. 43. **Hammett value analysis.** pKa values for 4-hydroxybenzoic acid, 3-hydroxybenzoic acid, 4-((4-bromophenyl)sulfonamido)benzoic acid, and 3-((4-bromophenyl)sulfonamido)benzoic acid were calculated using Advanced Chemistry Development (ACD/Labs) Software V11.02. Hammett values were derived by applying calculated pKa values to the Hammett equation.[12]

F. NMR spectra

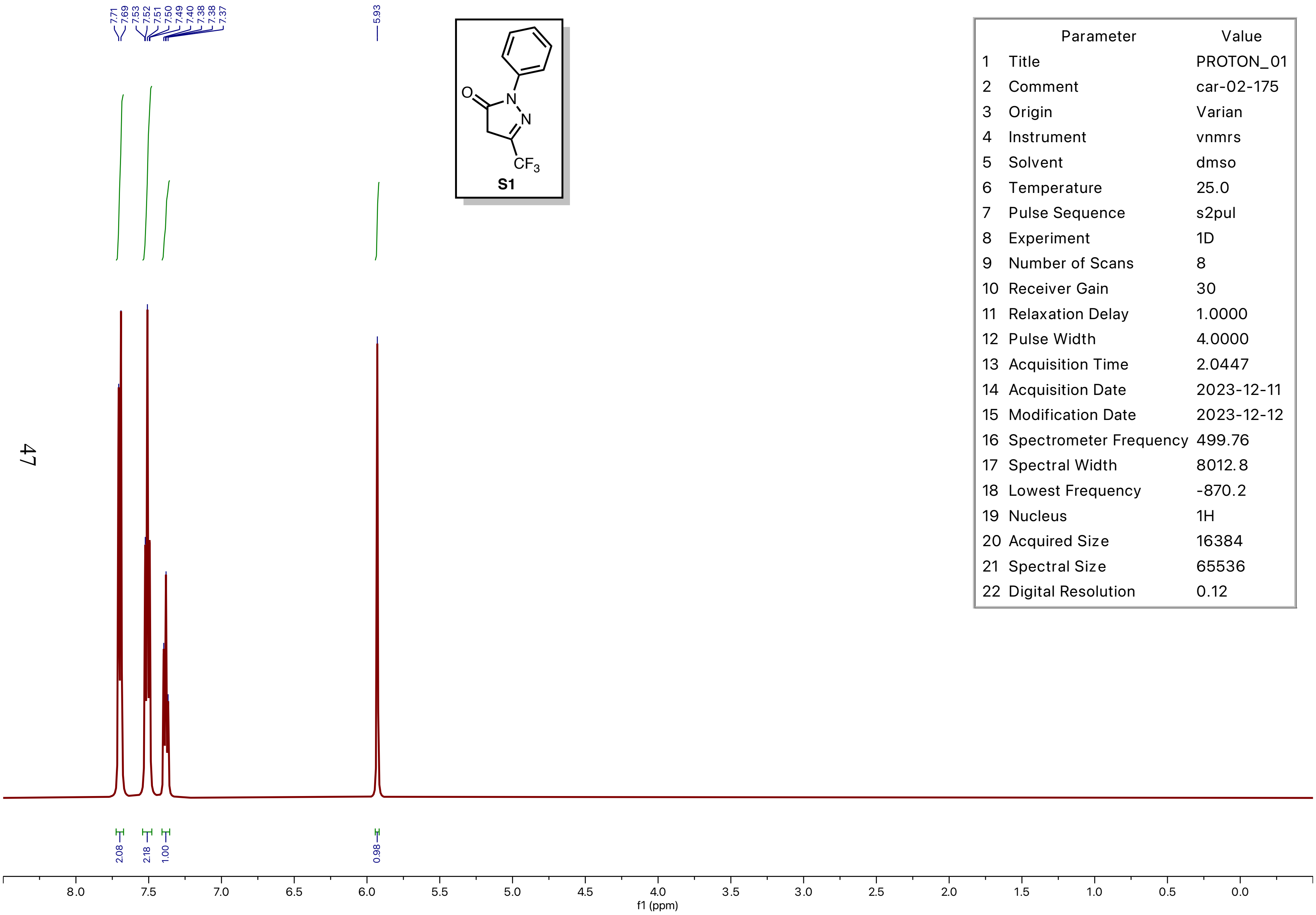

	Parameter	Value
1	Title	PROTON_01
2	Comment	car-02-175
3	Origin	Varian
4	Instrument	vnmrs
5	Solvent	dmso
6	Temperature	25.0
7	Pulse Sequence	s2pul
8	Experiment	1D
9	Number of Scans	8
10	Receiver Gain	30
11	Relaxation Delay	1.0000
12	Pulse Width	4.0000
13	Acquisition Time	2.0447
14	Acquisition Date	2023-12-11
15	Modification Date	2023-12-12
16	Spectrometer Frequency	499.76
17	Spectral Width	8012.8
18	Lowest Frequency	-870.2
19	Nucleus	1H
20	Acquired Size	16384
21	Spectral Size	65536
22	Digital Resolution	0.12

Supplementary Fig. 44. **^{1}H NMR (500 MHz, DMSO-d_6) of trifluoromethyl pyrazolone S1.**

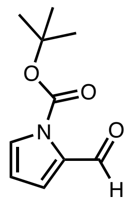

2c

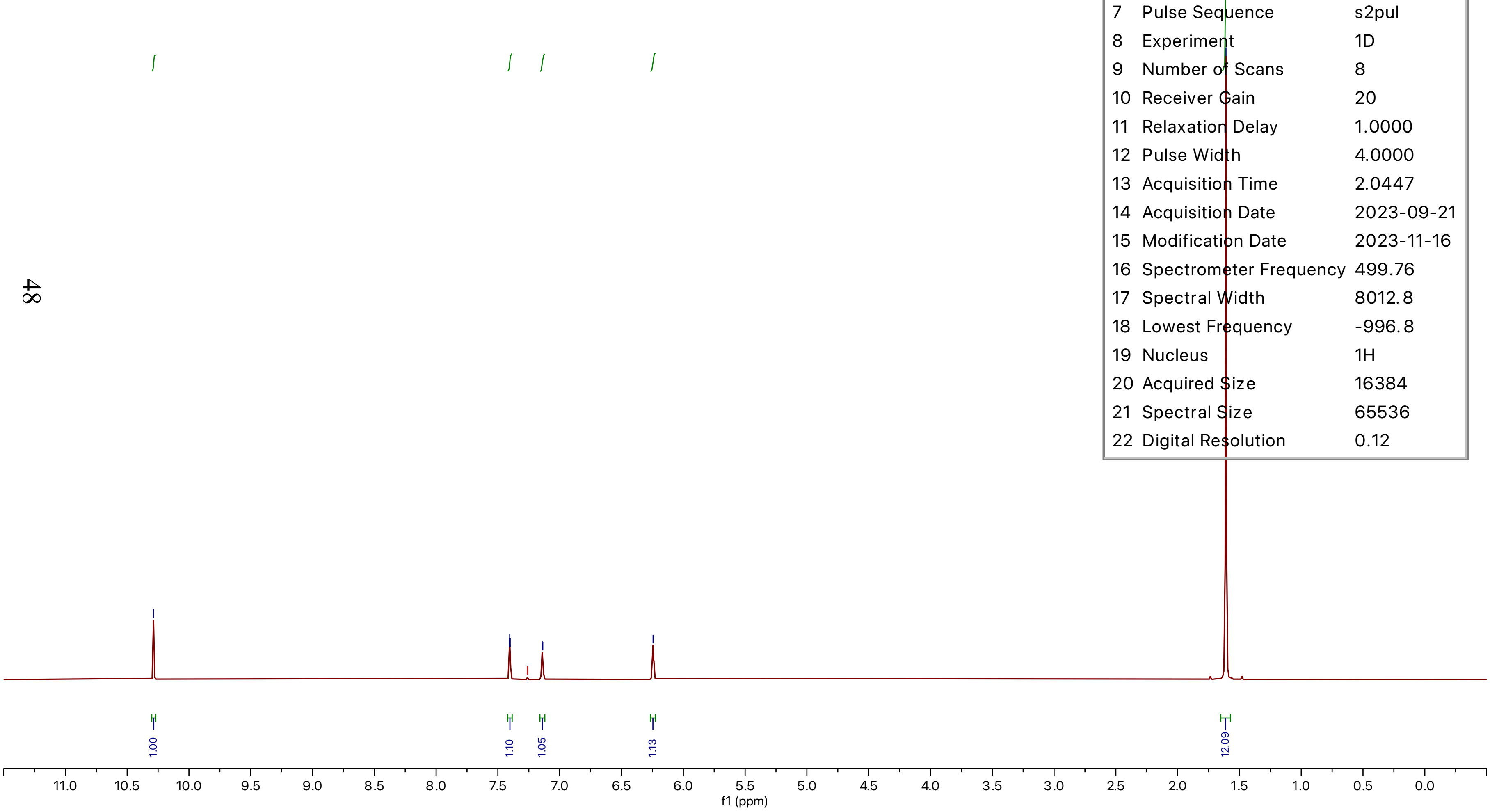

	Parameter	Value
1	Title	PROTON_01
2	Comment	car-02-128
3	Origin	Varian
4	Instrument	vnmrs
5	Solvent	cdcl3
6	Temperature	25.0
7	Pulse Sequence	s2pul
8	Experiment	1D
9	Number of Scans	8
10	Receiver Gain	20
11	Relaxation Delay	1.0000
12	Pulse Width	4.0000
13	Acquisition Time	2.0447
14	Acquisition Date	2023-09-21
15	Modification Date	2023-11-16
16	Spectrometer Frequency	499.76
17	Spectral Width	8012.8
18	Lowest Frequency	-996.8
19	Nucleus	1H
20	Acquired Size	16384
21	Spectral Size	65536
22	Digital Resolution	0.12

Supplementary Fig. 45. ^{1}**H NMR (500 MHz, $CDCl_3$) of pyrrole 2c.**

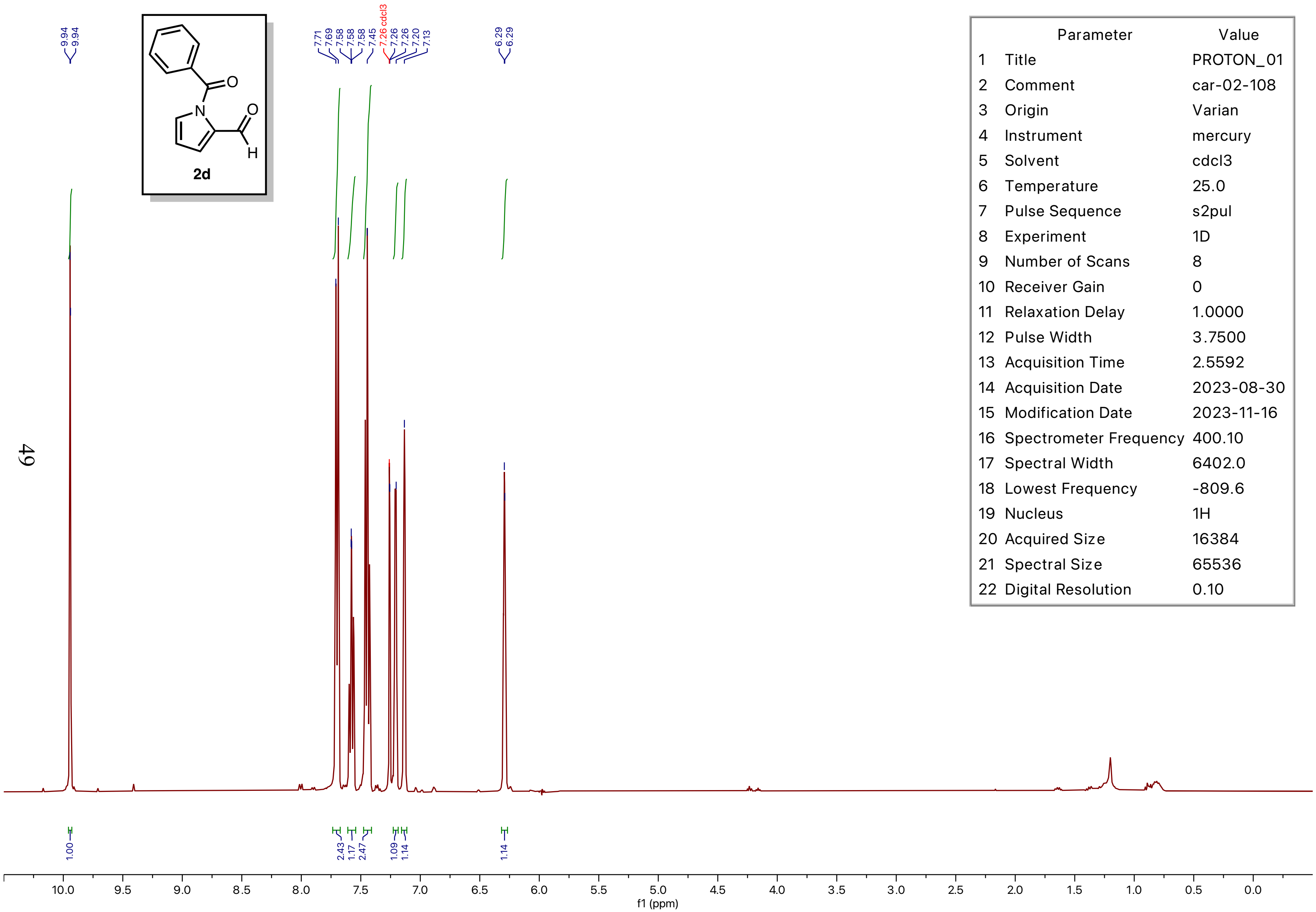

	Parameter	Value
1	Title	PROTON_01
2	Comment	car-02-108
3	Origin	Varian
4	Instrument	mercury
5	Solvent	cdcl3
6	Temperature	25.0
7	Pulse Sequence	s2pul
8	Experiment	1D
9	Number of Scans	8
10	Receiver Gain	0
11	Relaxation Delay	1.0000
12	Pulse Width	3.7500
13	Acquisition Time	2.5592
14	Acquisition Date	2023-08-30
15	Modification Date	2023-11-16
16	Spectrometer Frequency	400.10
17	Spectral Width	6402.0
18	Lowest Frequency	-809.6
19	Nucleus	1H
20	Acquired Size	16384
21	Spectral Size	65536
22	Digital Resolution	0.10

Supplementary Fig. 46. **^{1}H NMR (400 MHz, $CDCl_3$) of pyrrole 2d.**

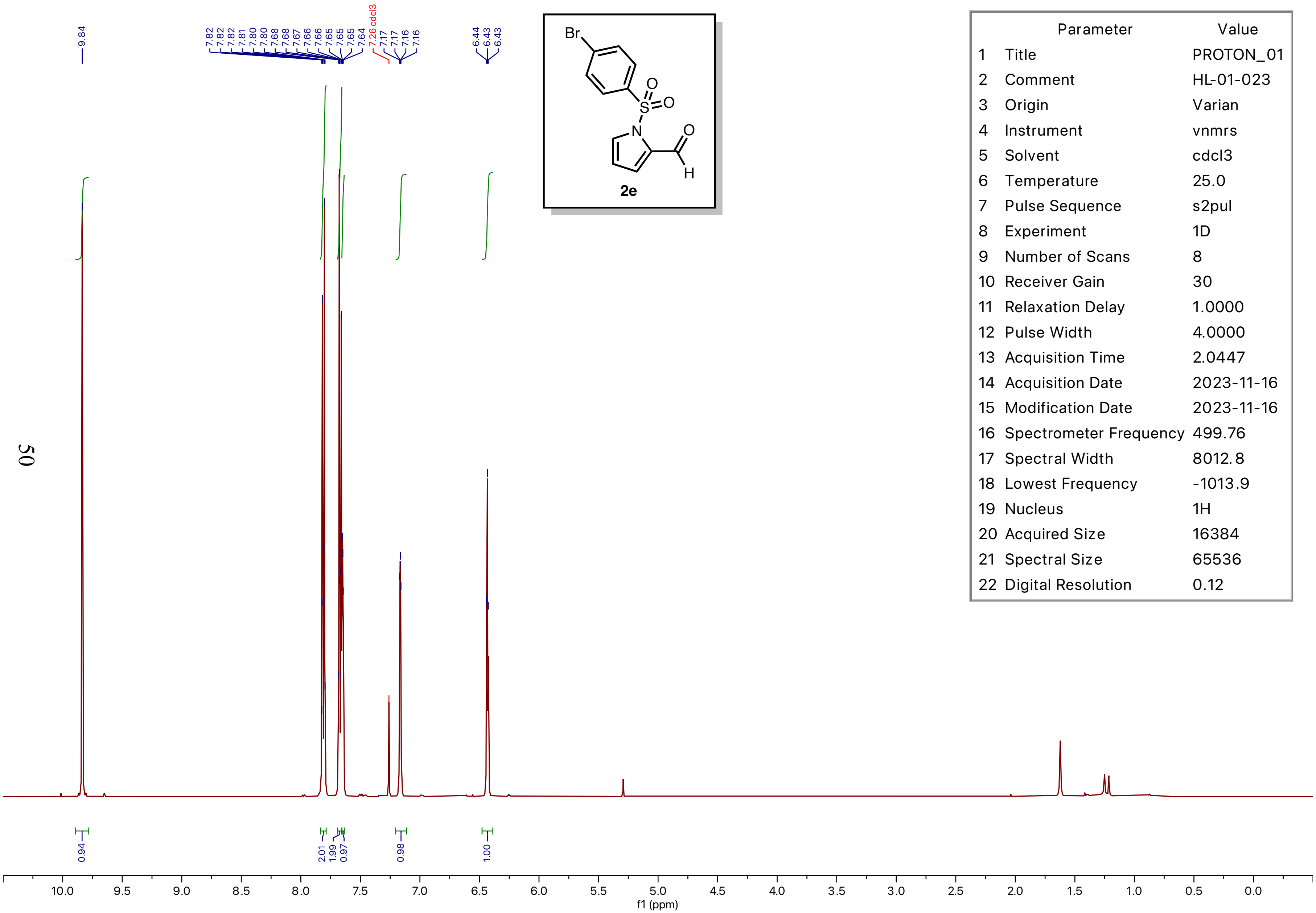

	Parameter	Value
1	Title	PROTON_01
2	Comment	HL-01-023
3	Origin	Varian
4	Instrument	vnmrs
5	Solvent	cdcl3
6	Temperature	25.0
7	Pulse Sequence	s2pul
8	Experiment	1D
9	Number of Scans	8
10	Receiver Gain	30
11	Relaxation Delay	1.0000
12	Pulse Width	4.0000
13	Acquisition Time	2.0447
14	Acquisition Date	2023-11-16
15	Modification Date	2023-11-16
16	Spectrometer Frequency	499.76
17	Spectral Width	8012.8
18	Lowest Frequency	-1013.9
19	Nucleus	1H
20	Acquired Size	16384
21	Spectral Size	65536
22	Digital Resolution	0.12

Supplementary Fig. 47. **^{1}H NMR (500 MHz, $CDCl_3$) of pyrrole 2e.**

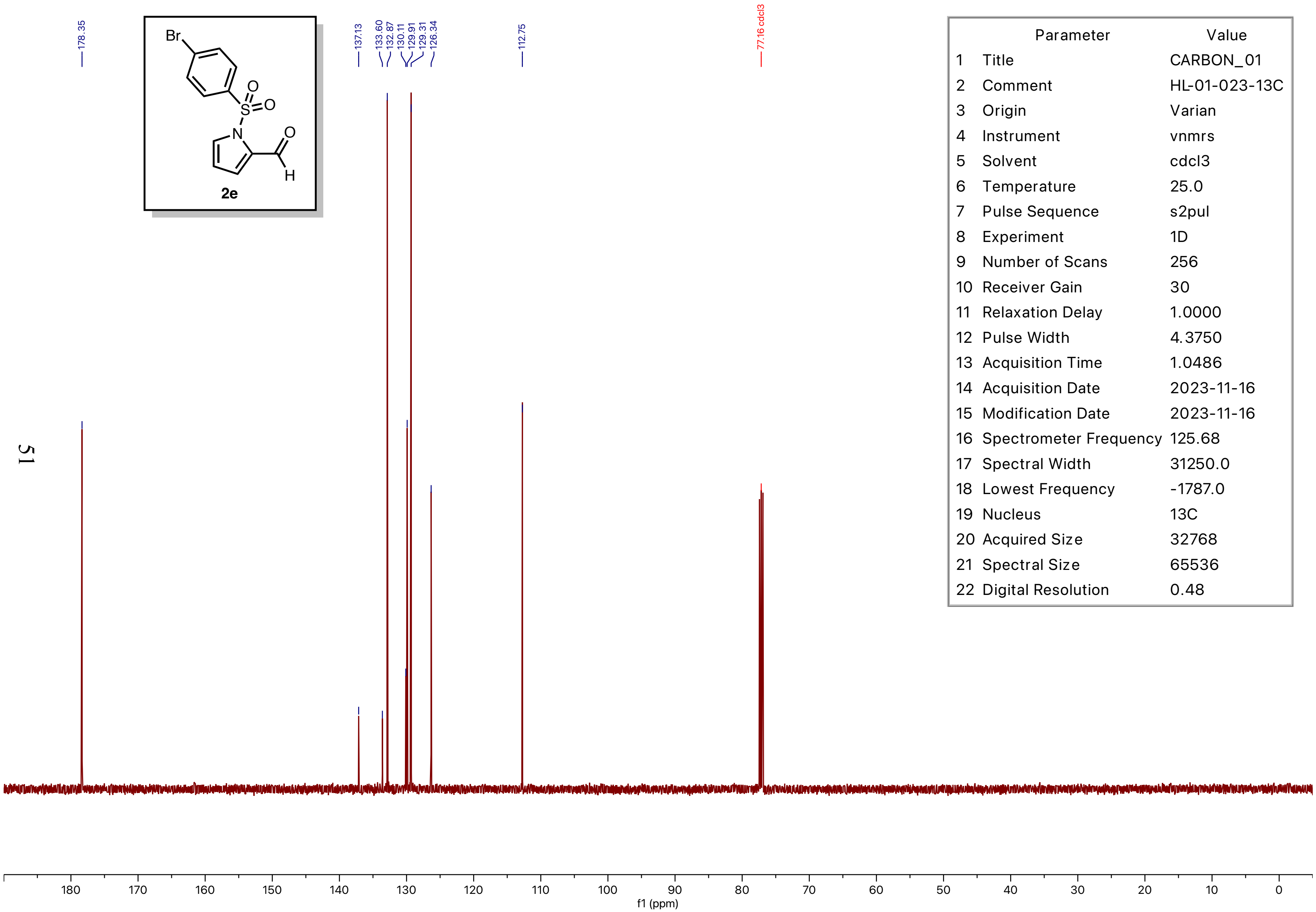

	Parameter	Value
1	Title	CARBON_01
2	Comment	HL-01-023-13C
3	Origin	Varian
4	Instrument	vnmrs
5	Solvent	cdcl3
6	Temperature	25.0
7	Pulse Sequence	s2pul
8	Experiment	1D
9	Number of Scans	256
10	Receiver Gain	30
11	Relaxation Delay	1.0000
12	Pulse Width	4.3750
13	Acquisition Time	1.0486
14	Acquisition Date	2023-11-16
15	Modification Date	2023-11-16
16	Spectrometer Frequency	125.68
17	Spectral Width	31250.0
18	Lowest Frequency	-1787.0
19	Nucleus	13C
20	Acquired Size	32768
21	Spectral Size	65536
22	Digital Resolution	0.48

Supplementary Fig. 48. **^{13}C NMR (125 MHz, $CDCl_3$) of pyrrole 2e.**

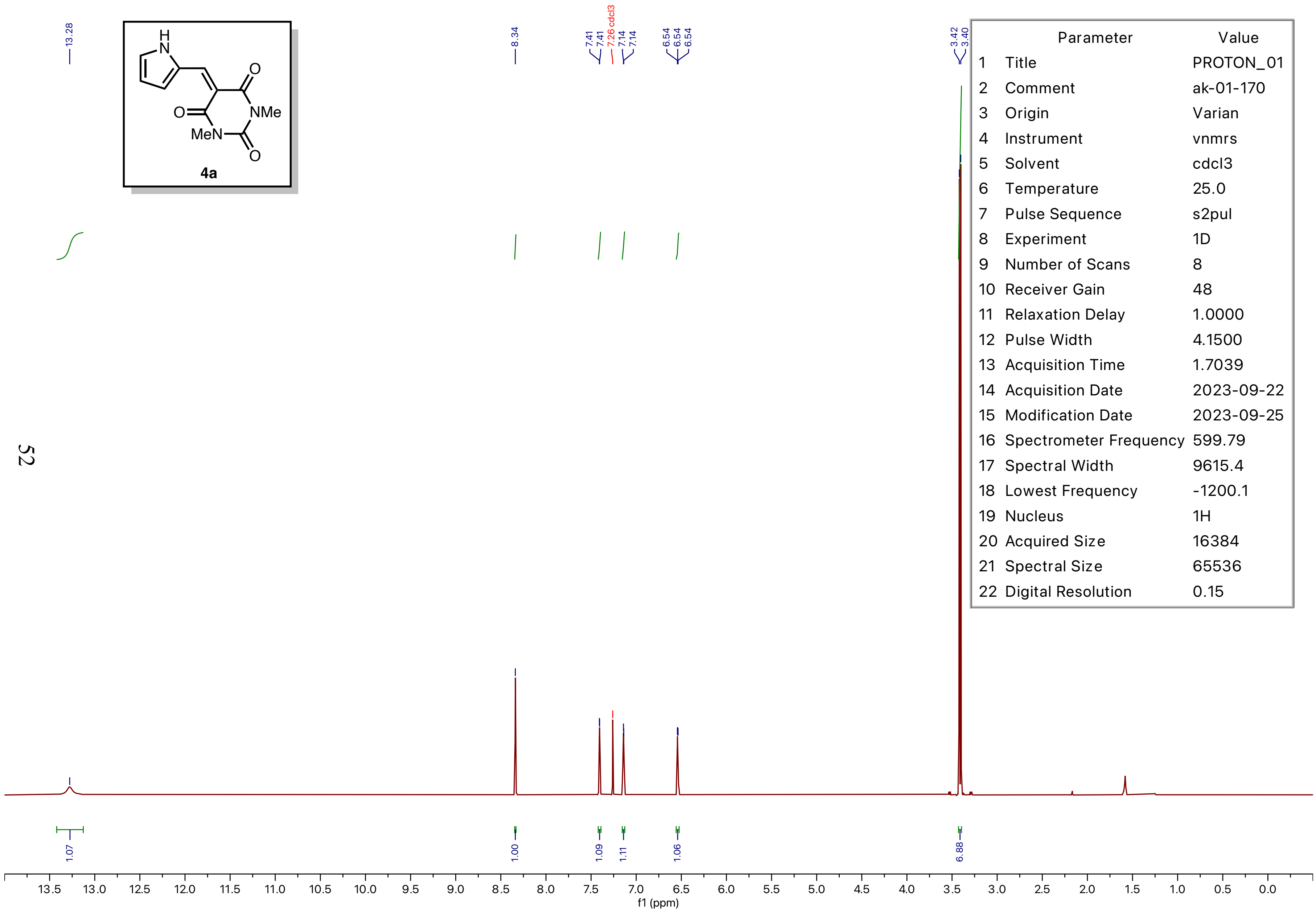

	Parameter	Value
1	Title	PROTON_01
2	Comment	ak-01-170
3	Origin	Varian
4	Instrument	vnmrs
5	Solvent	cdcl3
6	Temperature	25.0
7	Pulse Sequence	s2pul
8	Experiment	1D
9	Number of Scans	8
10	Receiver Gain	48
11	Relaxation Delay	1.0000
12	Pulse Width	4.1500
13	Acquisition Time	1.7039
14	Acquisition Date	2023-09-22
15	Modification Date	2023-09-25
16	Spectrometer Frequency	599.79
17	Spectral Width	9615.4
18	Lowest Frequency	-1200.1
19	Nucleus	1H
20	Acquired Size	16384
21	Spectral Size	65536
22	Digital Resolution	0.15

Supplementary Fig. 49. **^{1}H NMR (600 MHz, $CDCl_3$) of activated pyrrole 4a.**

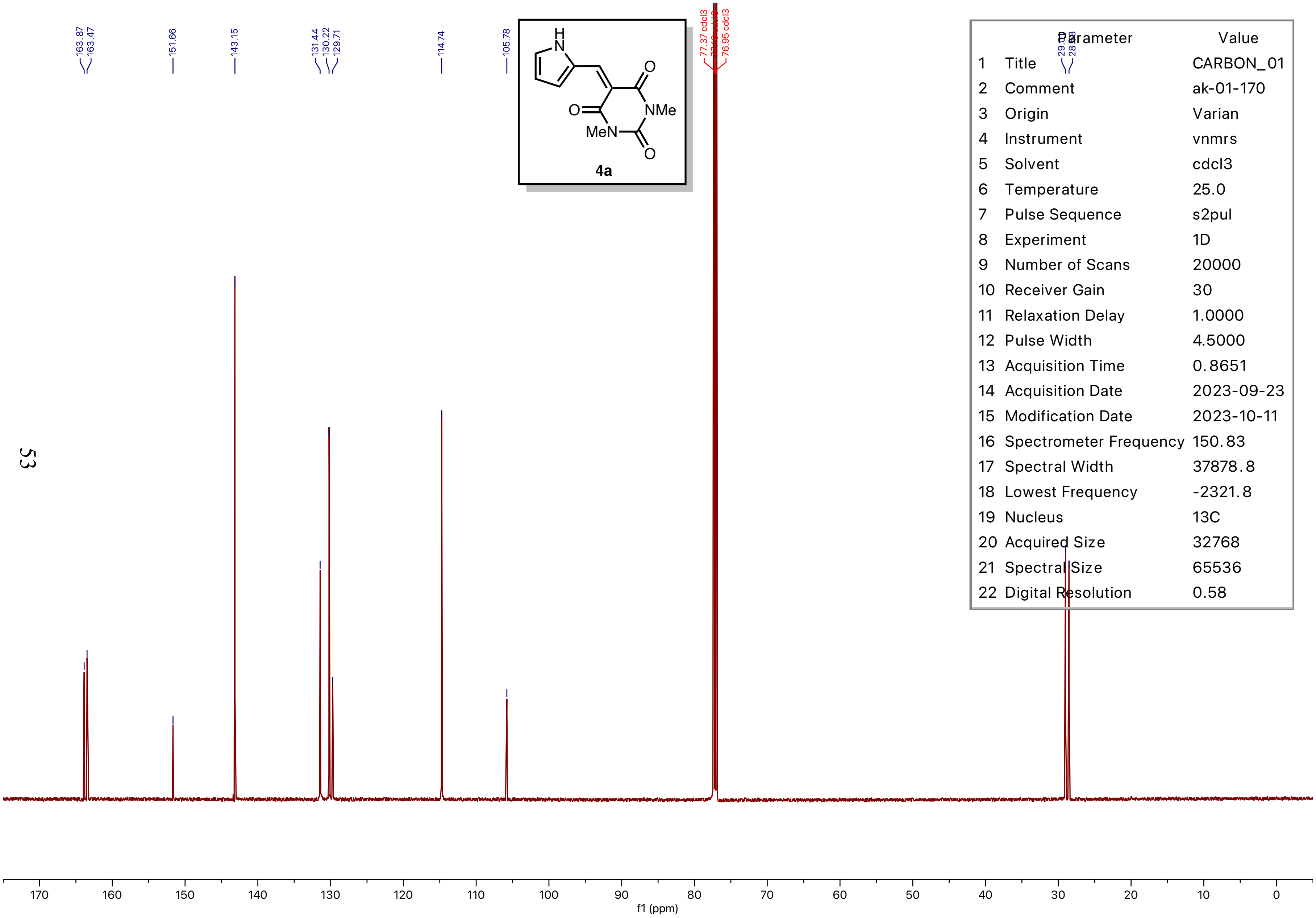

	Parameter	Value
1	Title	CARBON_01
2	Comment	ak-01-170
3	Origin	Varian
4	Instrument	vnmrs
5	Solvent	cdcl3
6	Temperature	25.0
7	Pulse Sequence	s2pul
8	Experiment	1D
9	Number of Scans	20000
10	Receiver Gain	30
11	Relaxation Delay	1.0000
12	Pulse Width	4.5000
13	Acquisition Time	0.8651
14	Acquisition Date	2023-09-23
15	Modification Date	2023-10-11
16	Spectrometer Frequency	150.83
17	Spectral Width	37878.8
18	Lowest Frequency	-2321.8
19	Nucleus	13C
20	Acquired Size	32768
21	Spectral Size	65536
22	Digital Resolution	0.58

Supplementary Fig. 50. **^{13}C NMR (150 MHz, $CDCl_3$) of activated pyrrole 4a.**

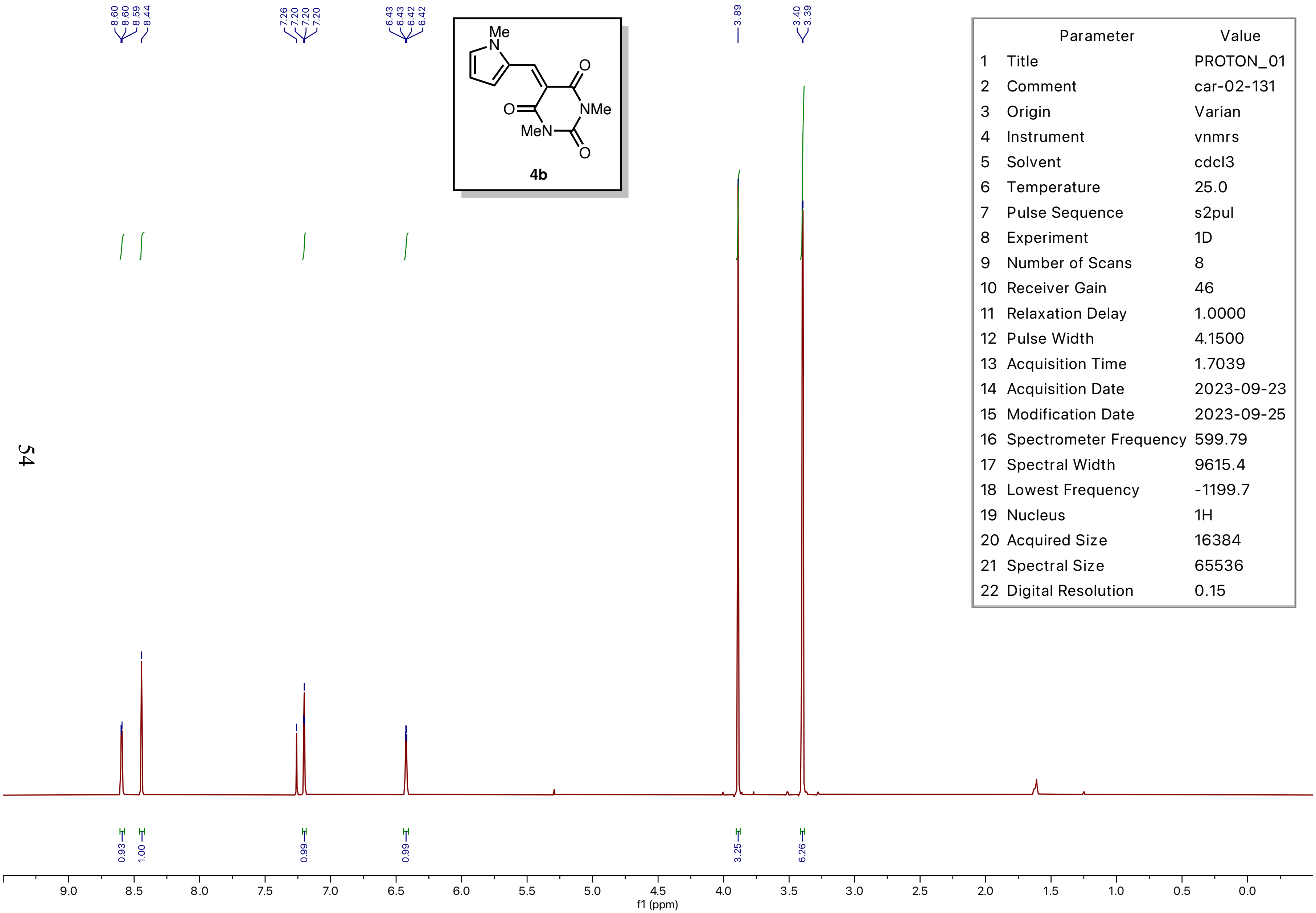

	Parameter	Value
1	Title	PROTON_01
2	Comment	car-02-131
3	Origin	Varian
4	Instrument	vnmrs
5	Solvent	cdcl3
6	Temperature	25.0
7	Pulse Sequence	s2pul
8	Experiment	1D
9	Number of Scans	8
10	Receiver Gain	46
11	Relaxation Delay	1.0000
12	Pulse Width	4.1500
13	Acquisition Time	1.7039
14	Acquisition Date	2023-09-23
15	Modification Date	2023-09-25
16	Spectrometer Frequency	599.79
17	Spectral Width	9615.4
18	Lowest Frequency	-1199.7
19	Nucleus	1H
20	Acquired Size	16384
21	Spectral Size	65536
22	Digital Resolution	0.15

Supplementary Fig. 51. **^{1}H NMR (600 MHz, $CDCl_3$) of activated pyrrole 4b.**

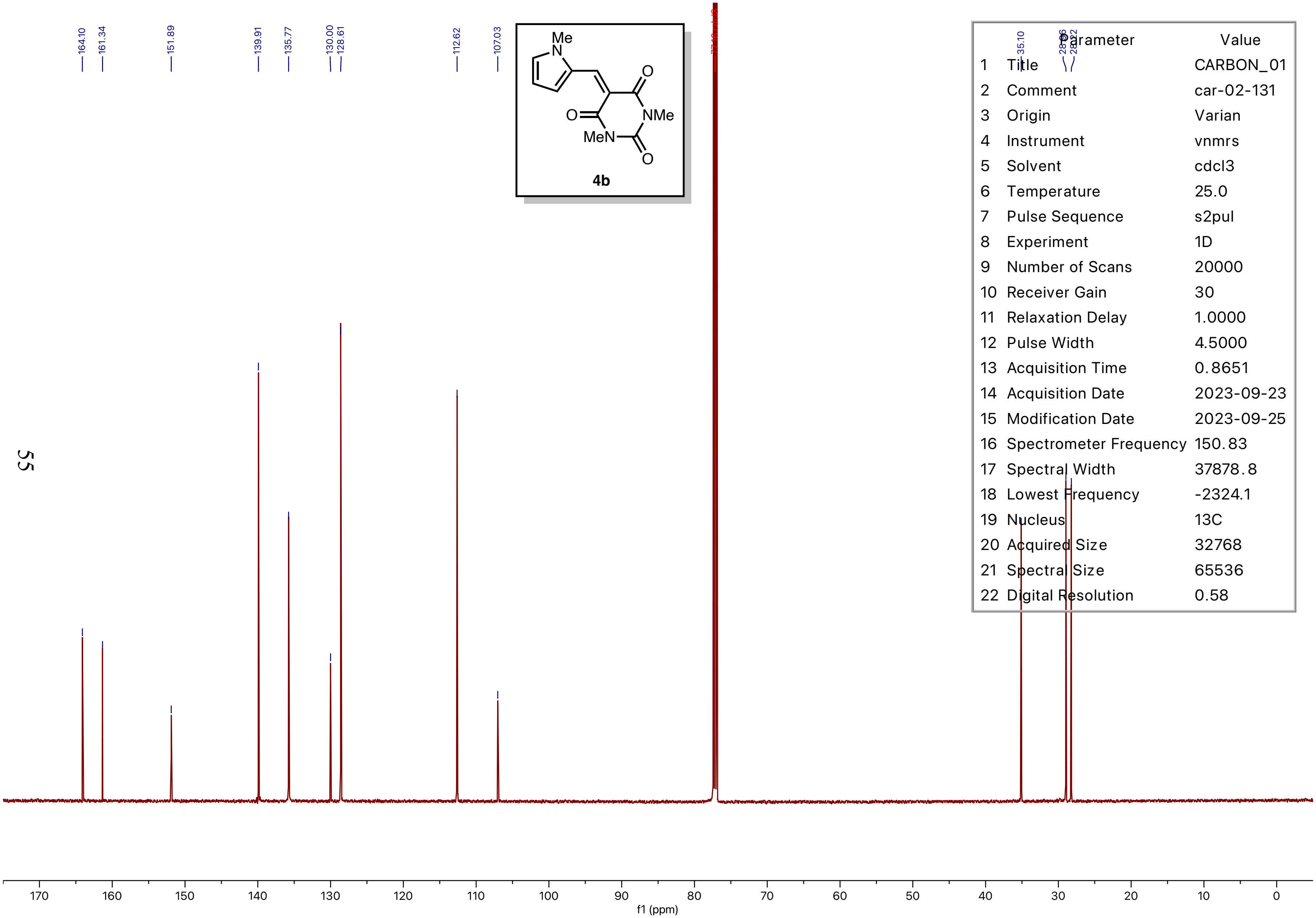

	Parameter	Value
1	Title	CARBON_01
2	Comment	car-02-131
3	Origin	Varian
4	Instrument	vnmrs
5	Solvent	cdcl3
6	Temperature	25.0
7	Pulse Sequence	s2pul
8	Experiment	1D
9	Number of Scans	20000
10	Receiver Gain	30
11	Relaxation Delay	1.0000
12	Pulse Width	4.5000
13	Acquisition Time	0.8651
14	Acquisition Date	2023-09-23
15	Modification Date	2023-09-25
16	Spectrometer Frequency	150.83
17	Spectral Width	37878.8
18	Lowest Frequency	-2324.1
19	Nucleus	13C
20	Acquired Size	32768
21	Spectral Size	65536
22	Digital Resolution	0.58

Supplementary Fig. 52. **^{13}C NMR (150 MHz, $CDCl_3$) of activated pyrrole 4b.**

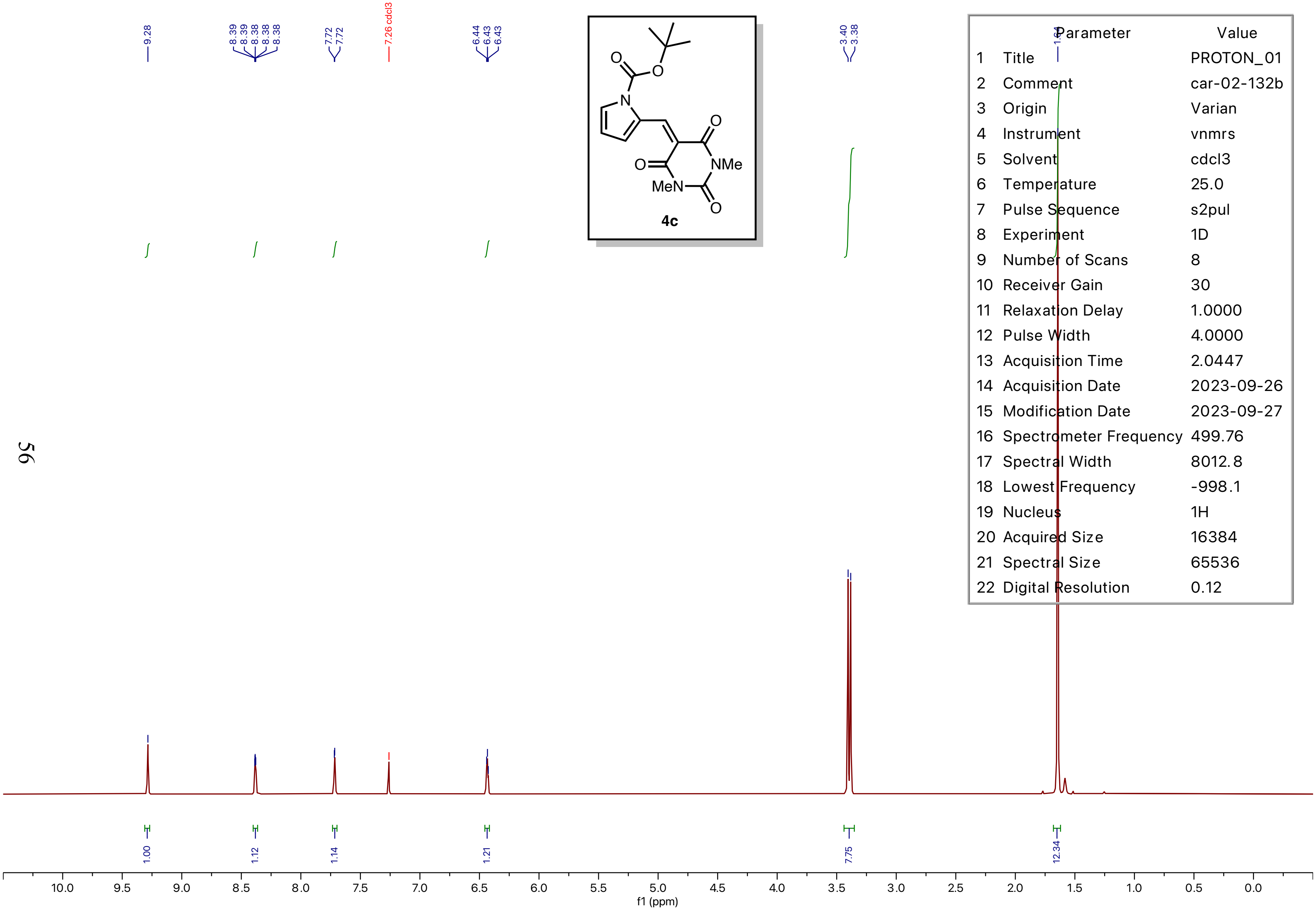

Supplemetary Fig. 53. **^{1}H NMR (500 MHz, $CDCl_3$) of activated pyrrole 4c.**

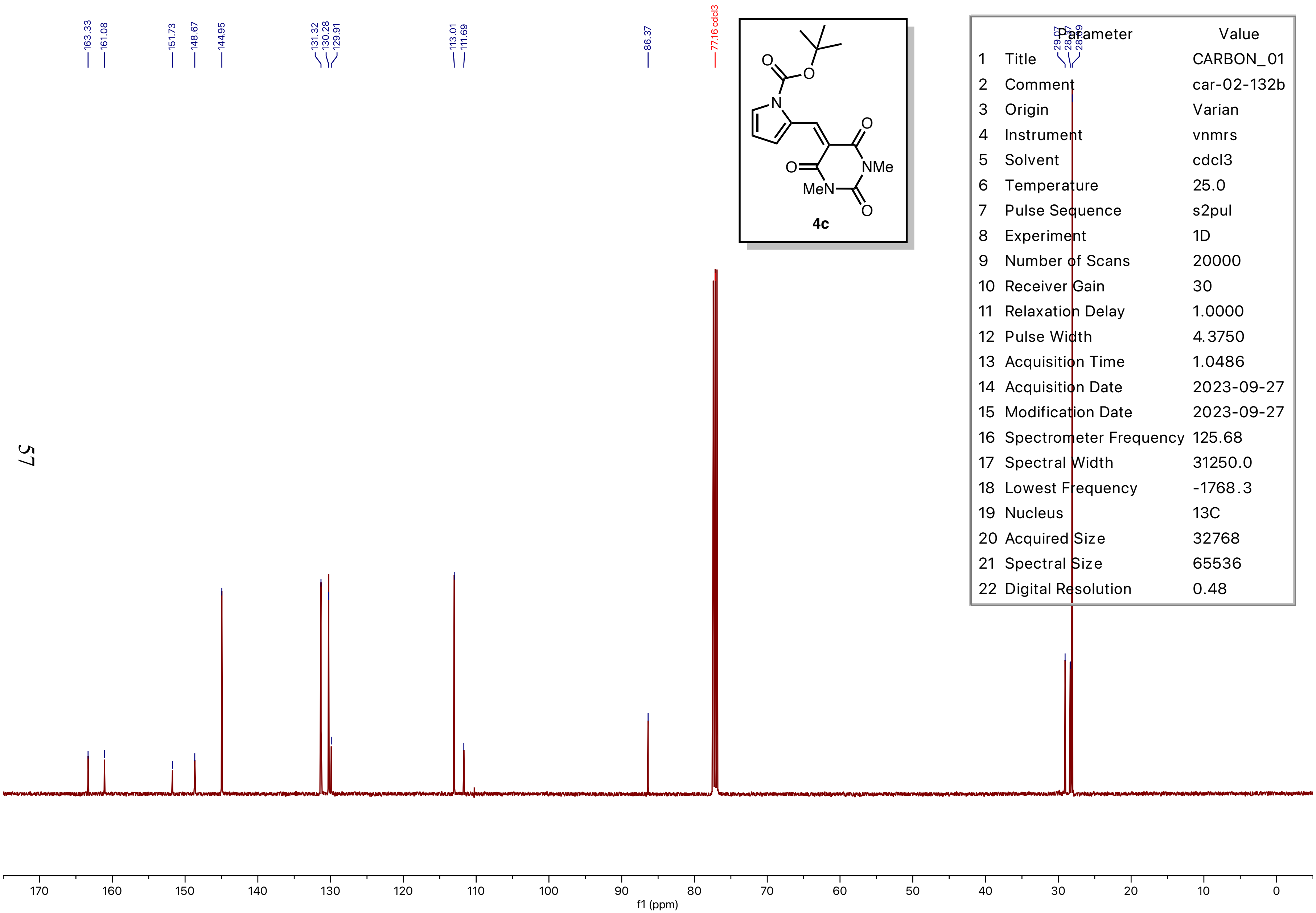

	Parameter	Value
1	Title	CARBON_01
2	Comment	car-02-132b
3	Origin	Varian
4	Instrument	vnmrs
5	Solvent	cdcl3
6	Temperature	25.0
7	Pulse Sequence	s2pul
8	Experiment	1D
9	Number of Scans	20000
10	Receiver Gain	30
11	Relaxation Delay	1.0000
12	Pulse Width	4.3750
13	Acquisition Time	1.0486
14	Acquisition Date	2023-09-27
15	Modification Date	2023-09-27
16	Spectrometer Frequency	125.68
17	Spectral Width	31250.0
18	Lowest Frequency	-1768.3
19	Nucleus	13C
20	Acquired Size	32768
21	Spectral Size	65536
22	Digital Resolution	0.48

Supplementary Fig. 54. **^{13}C NMR (125 MHz, $CDCl_3$) of activated pyrrole 4c.**

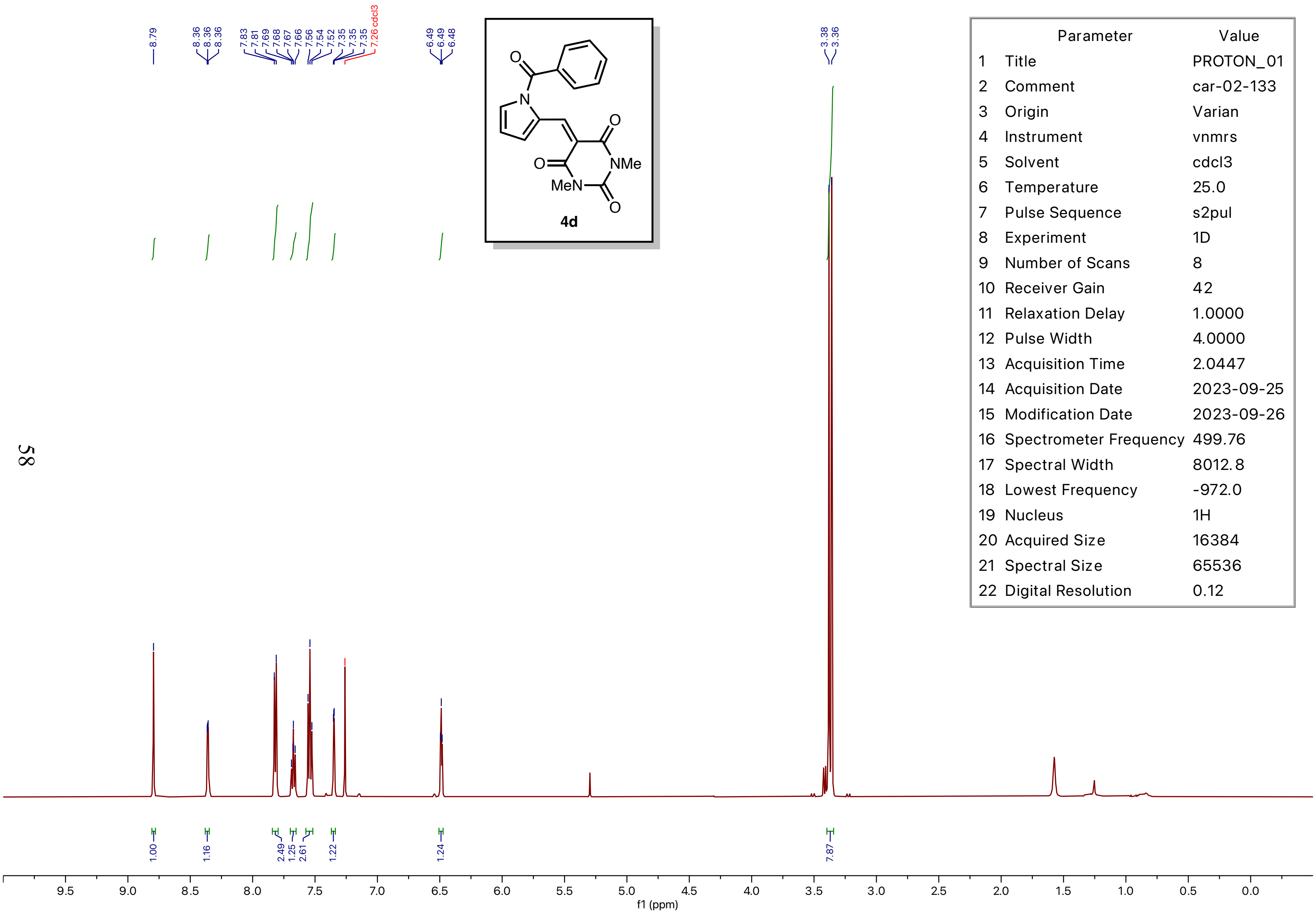

	Parameter	Value
1	Title	PROTON_01
2	Comment	car-02-133
3	Origin	Varian
4	Instrument	vnmrs
5	Solvent	cdcl3
6	Temperature	25.0
7	Pulse Sequence	s2pul
8	Experiment	1D
9	Number of Scans	8
10	Receiver Gain	42
11	Relaxation Delay	1.0000
12	Pulse Width	4.0000
13	Acquisition Time	2.0447
14	Acquisition Date	2023-09-25
15	Modification Date	2023-09-26
16	Spectrometer Frequency	499.76
17	Spectral Width	8012.8
18	Lowest Frequency	-972.0
19	Nucleus	1H
20	Acquired Size	16384
21	Spectral Size	65536
22	Digital Resolution	0.12

Supplementary Fig. 55. **^{1}H NMR (500 MHz, $CDCl_3$) of activated pyrrole 4d.**

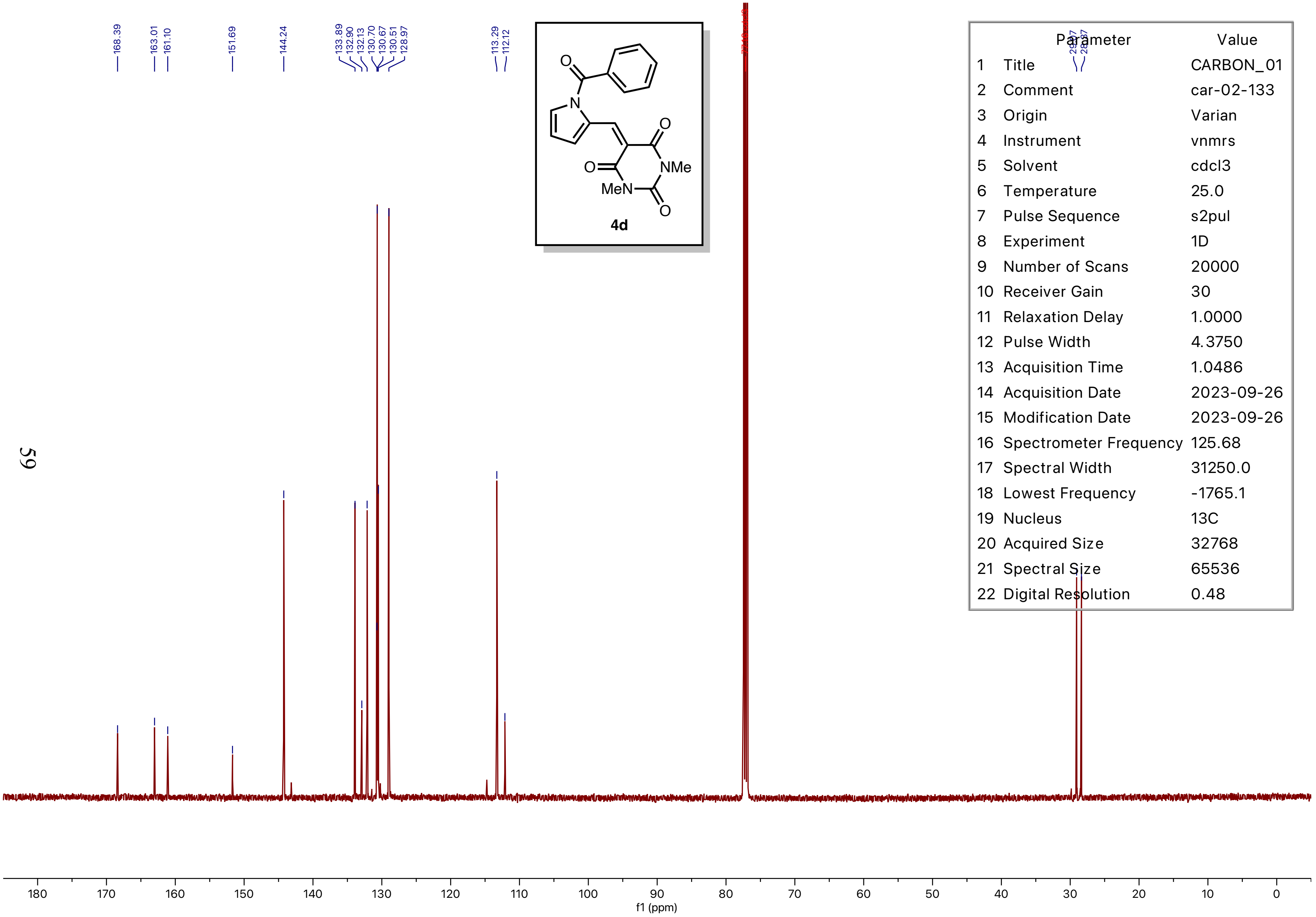

Supplementary Fig. 56. **^{13}C NMR (125 MHz, $CDCl_3$) of activated pyrrole 4d.**

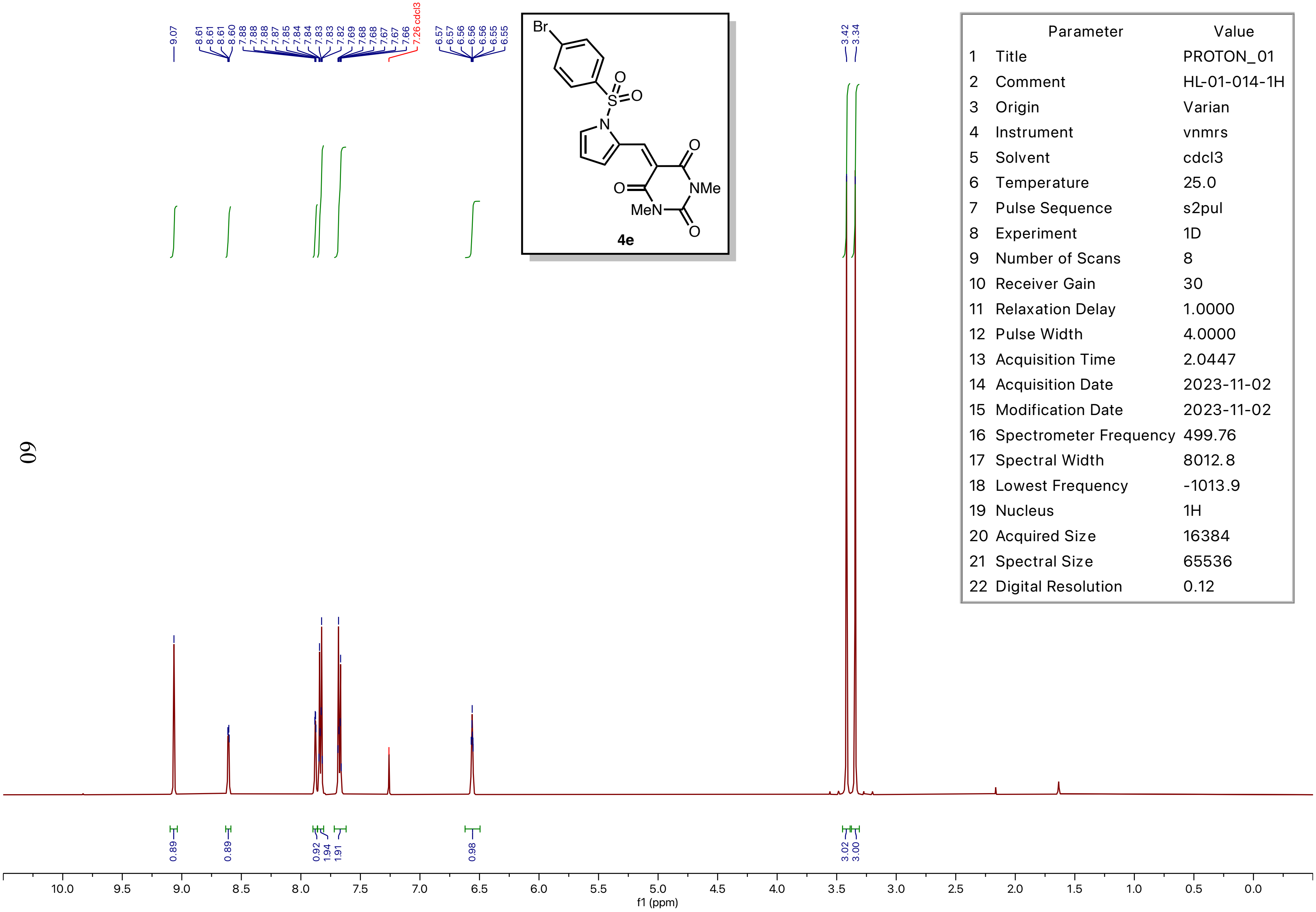

	Parameter	Value
1	Title	PROTON_01
2	Comment	HL-01-014-1H
3	Origin	Varian
4	Instrument	vnmrs
5	Solvent	cdcl3
6	Temperature	25.0
7	Pulse Sequence	s2pul
8	Experiment	1D
9	Number of Scans	8
10	Receiver Gain	30
11	Relaxation Delay	1.0000
12	Pulse Width	4.0000
13	Acquisition Time	2.0447
14	Acquisition Date	2023-11-02
15	Modification Date	2023-11-02
16	Spectrometer Frequency	499.76
17	Spectral Width	8012.8
18	Lowest Frequency	-1013.9
19	Nucleus	1H
20	Acquired Size	16384
21	Spectral Size	65536
22	Digital Resolution	0.12

Supplementary Fig. 57. **^{1}H NMR (500 MHz, $CDCl_3$) of activated pyrrole 4e.**

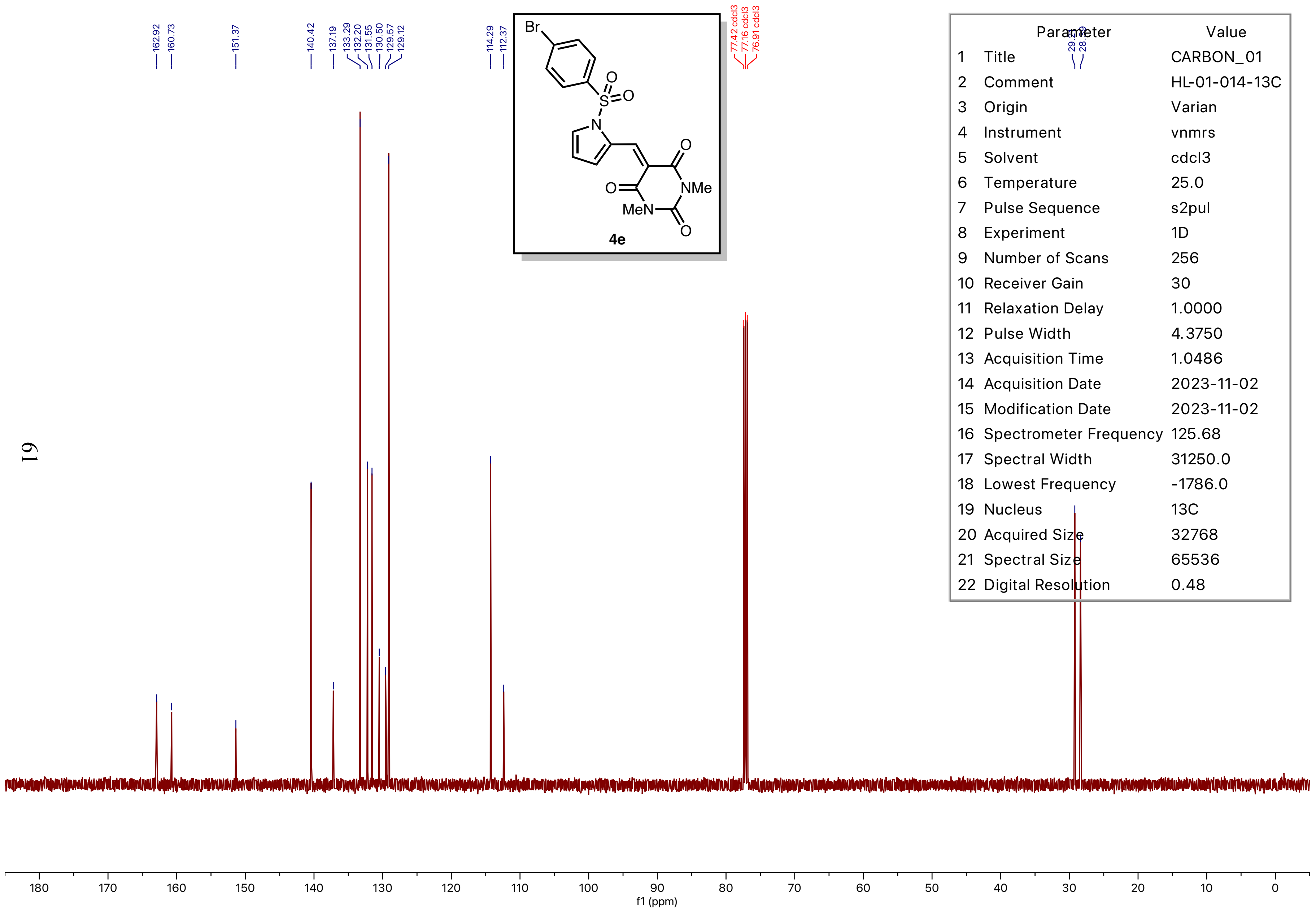

	Parameter	Value
1	Title	CARBON_01
2	Comment	HL-01-014-13C
3	Origin	Varian
4	Instrument	vnmrs
5	Solvent	cdcl3
6	Temperature	25.0
7	Pulse Sequence	s2pul
8	Experiment	1D
9	Number of Scans	256
10	Receiver Gain	30
11	Relaxation Delay	1.0000
12	Pulse Width	4.3750
13	Acquisition Time	1.0486
14	Acquisition Date	2023-11-02
15	Modification Date	2023-11-02
16	Spectrometer Frequency	125.68
17	Spectral Width	31250.0
18	Lowest Frequency	-1786.0
19	Nucleus	13C
20	Acquired Size	32768
21	Spectral Size	65536
22	Digital Resolution	0.48

Supplementary Fig. 58. **^{13}C NMR (125 MHz, $CDCl_3$) of activated pyrrole 4e.**

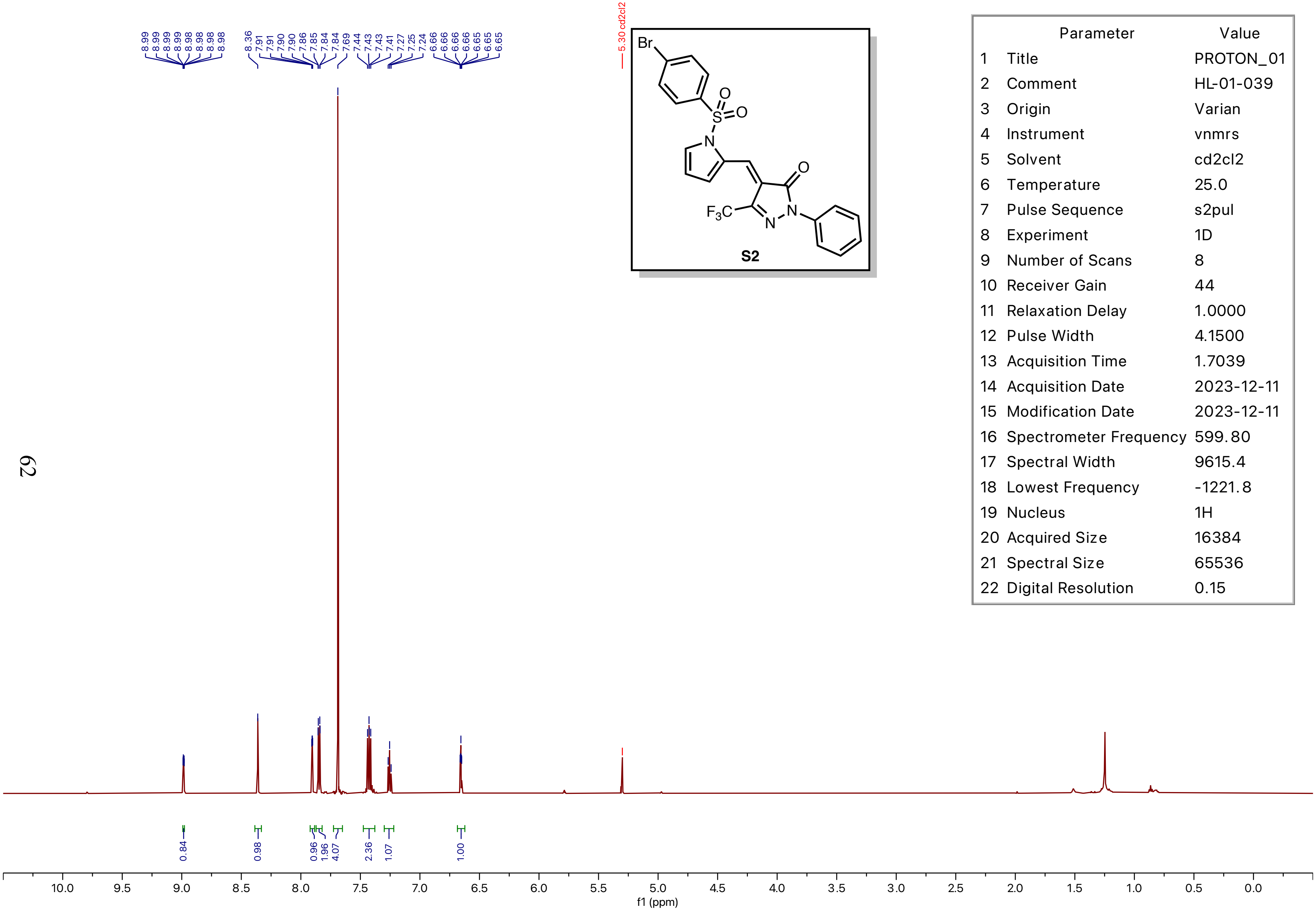

	Parameter	Value
1	Title	PROTON_01
2	Comment	HL-01-039
3	Origin	Varian
4	Instrument	vnmrs
5	Solvent	cd2cl2
6	Temperature	25.0
7	Pulse Sequence	s2pul
8	Experiment	1D
9	Number of Scans	8
10	Receiver Gain	44
11	Relaxation Delay	1.0000
12	Pulse Width	4.1500
13	Acquisition Time	1.7039
14	Acquisition Date	2023-12-11
15	Modification Date	2023-12-11
16	Spectrometer Frequency	599.80
17	Spectral Width	9615.4
18	Lowest Frequency	-1221.8
19	Nucleus	1H
20	Acquired Size	16384
21	Spectral Size	65536
22	Digital Resolution	0.15

Supplementary Fig. 59. **^{1}H NMR (600 MHz, CD_2Cl_2) of activated pyrrole S2.**

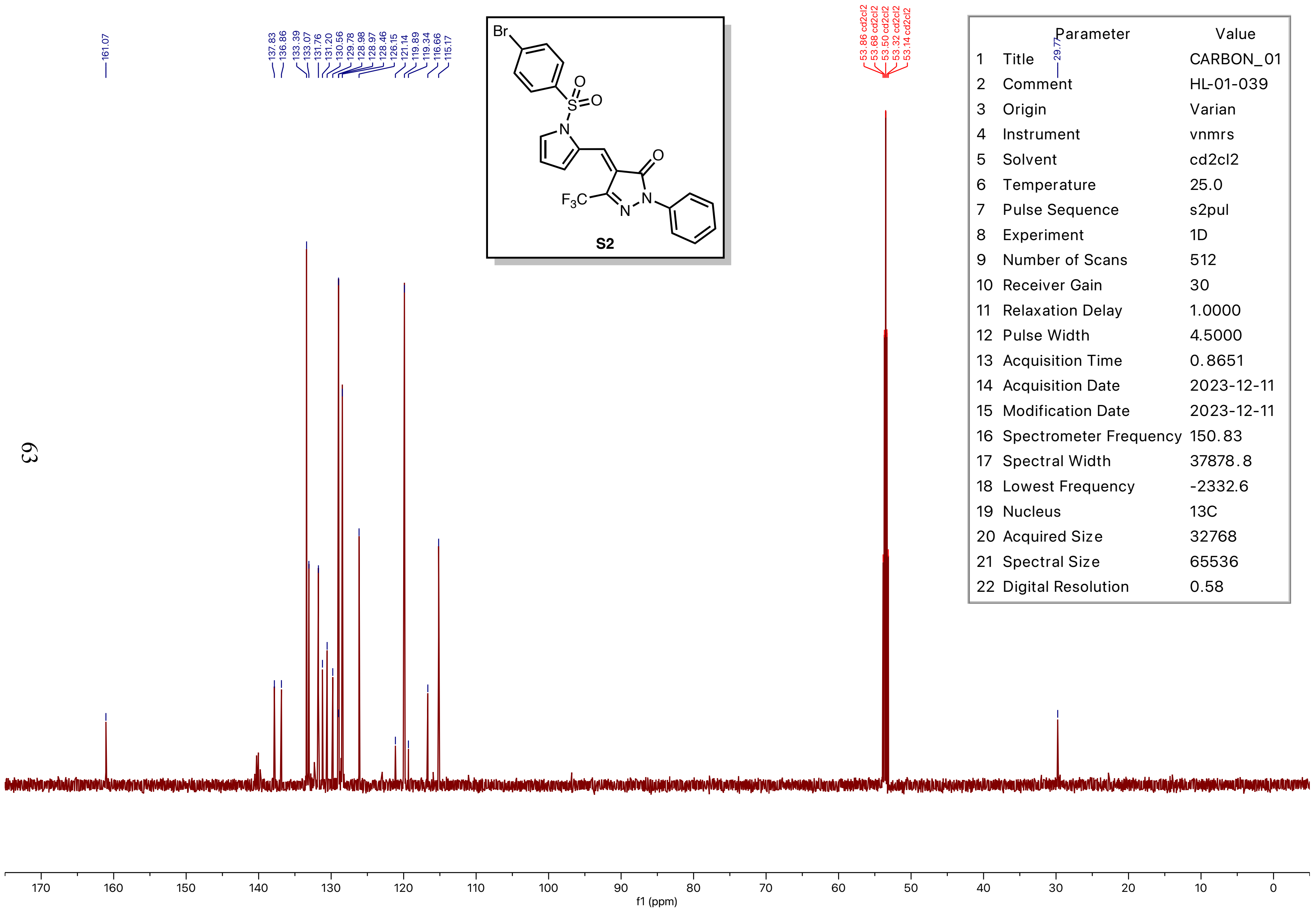

Supplementary Fig. 60. **^{13}C NMR (150 MHz, CD_2Cl_2) of activated pyrrole S2.**

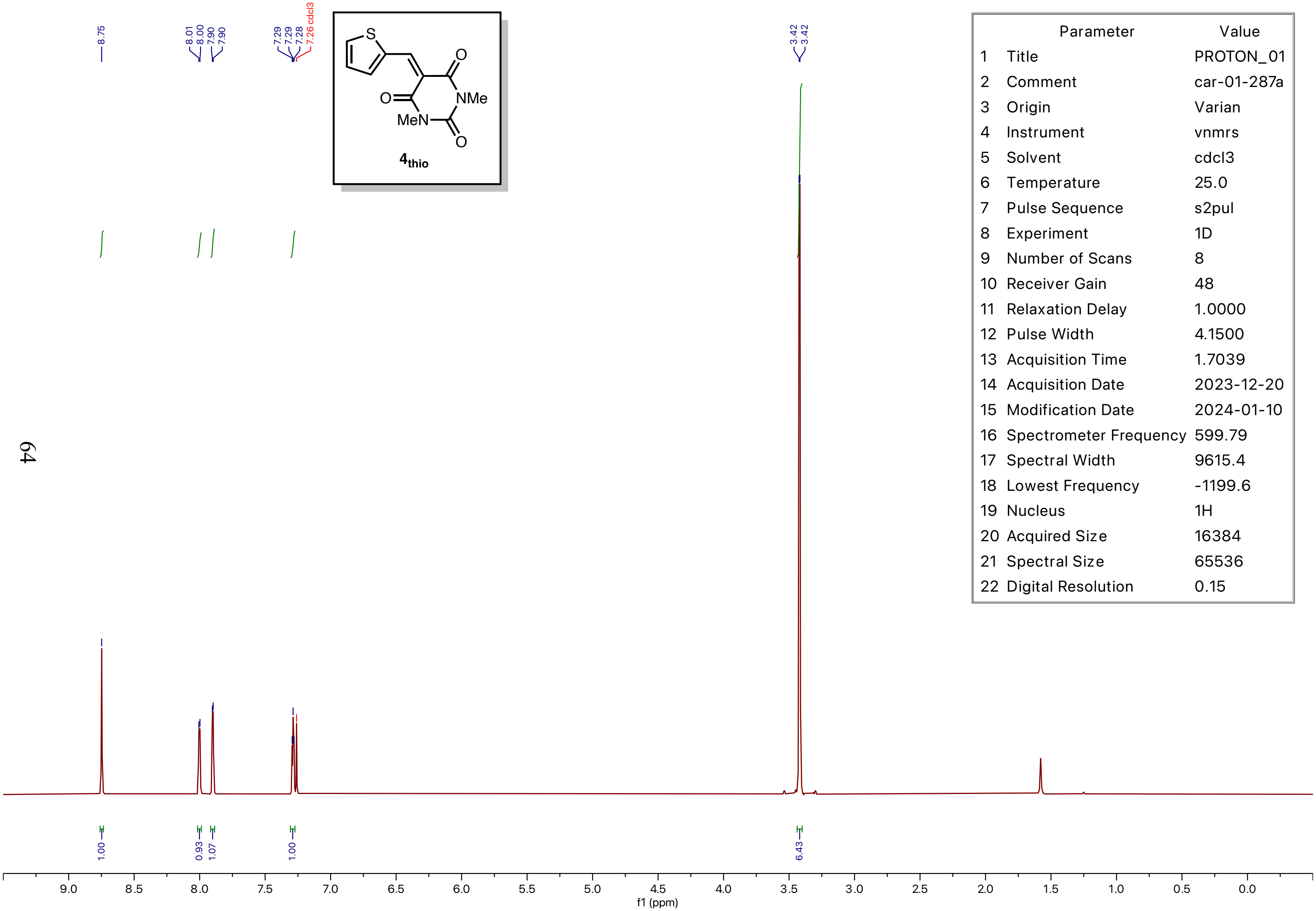

	Parameter	Value
1	Title	PROTON_01
2	Comment	car-01-287a
3	Origin	Varian
4	Instrument	vnmrs
5	Solvent	cdcl3
6	Temperature	25.0
7	Pulse Sequence	s2pul
8	Experiment	1D
9	Number of Scans	8
10	Receiver Gain	48
11	Relaxation Delay	1.0000
12	Pulse Width	4.1500
13	Acquisition Time	1.7039
14	Acquisition Date	2023-12-20
15	Modification Date	2024-01-10
16	Spectrometer Frequency	599.79
17	Spectral Width	9615.4
18	Lowest Frequency	-1199.6
19	Nucleus	1H
20	Acquired Size	16384
21	Spectral Size	65536
22	Digital Resolution	0.15

Supplementary Fig. 61. **^{1}H NMR (600 MHz, $CDCl_3$) of activated thiophene 4_{thio}.**

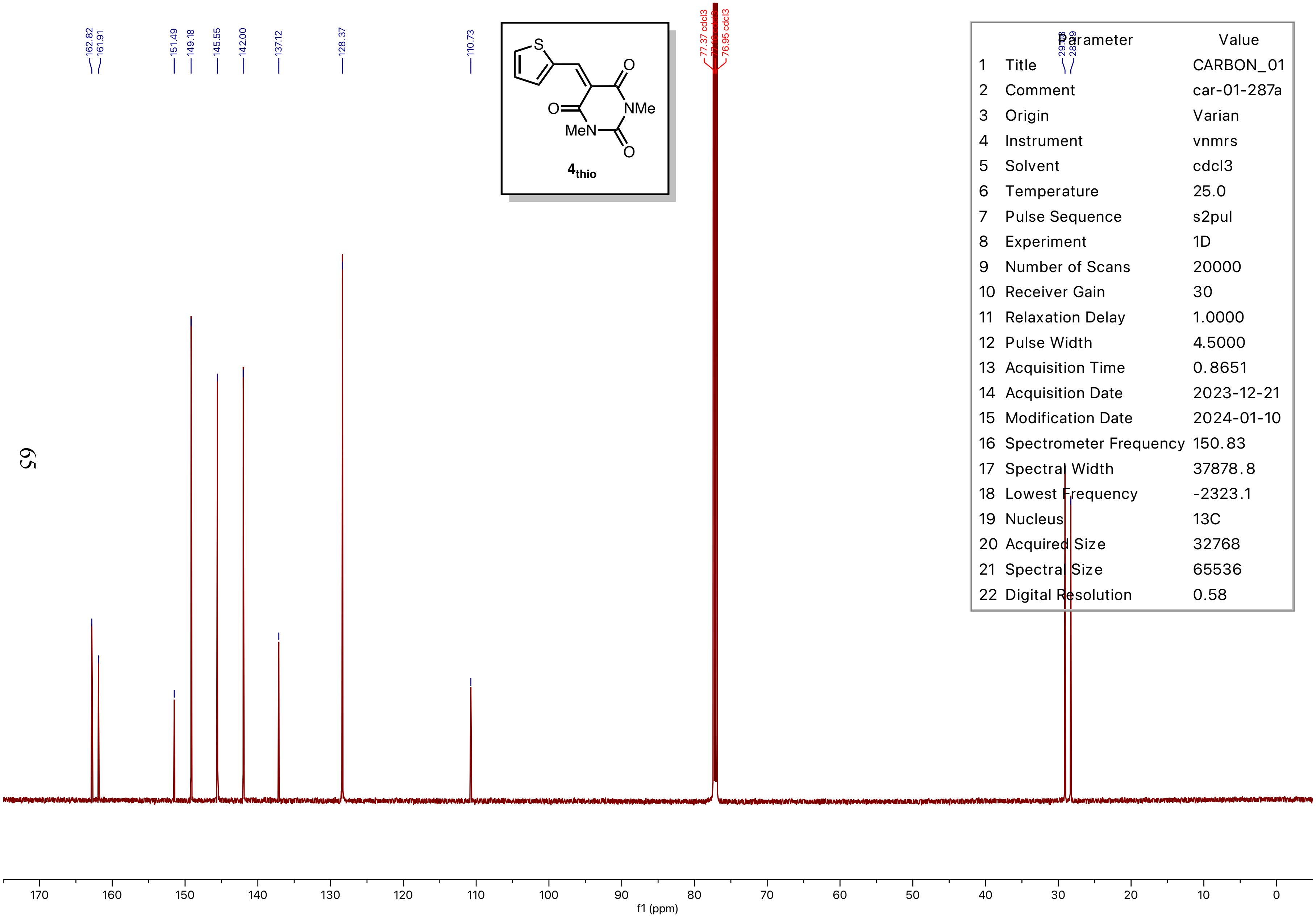

Supplementary Fig. 62. **^{13}C NMR (150 MHz, $CDCl_3$) of activated thiophene 4_{thio}.**

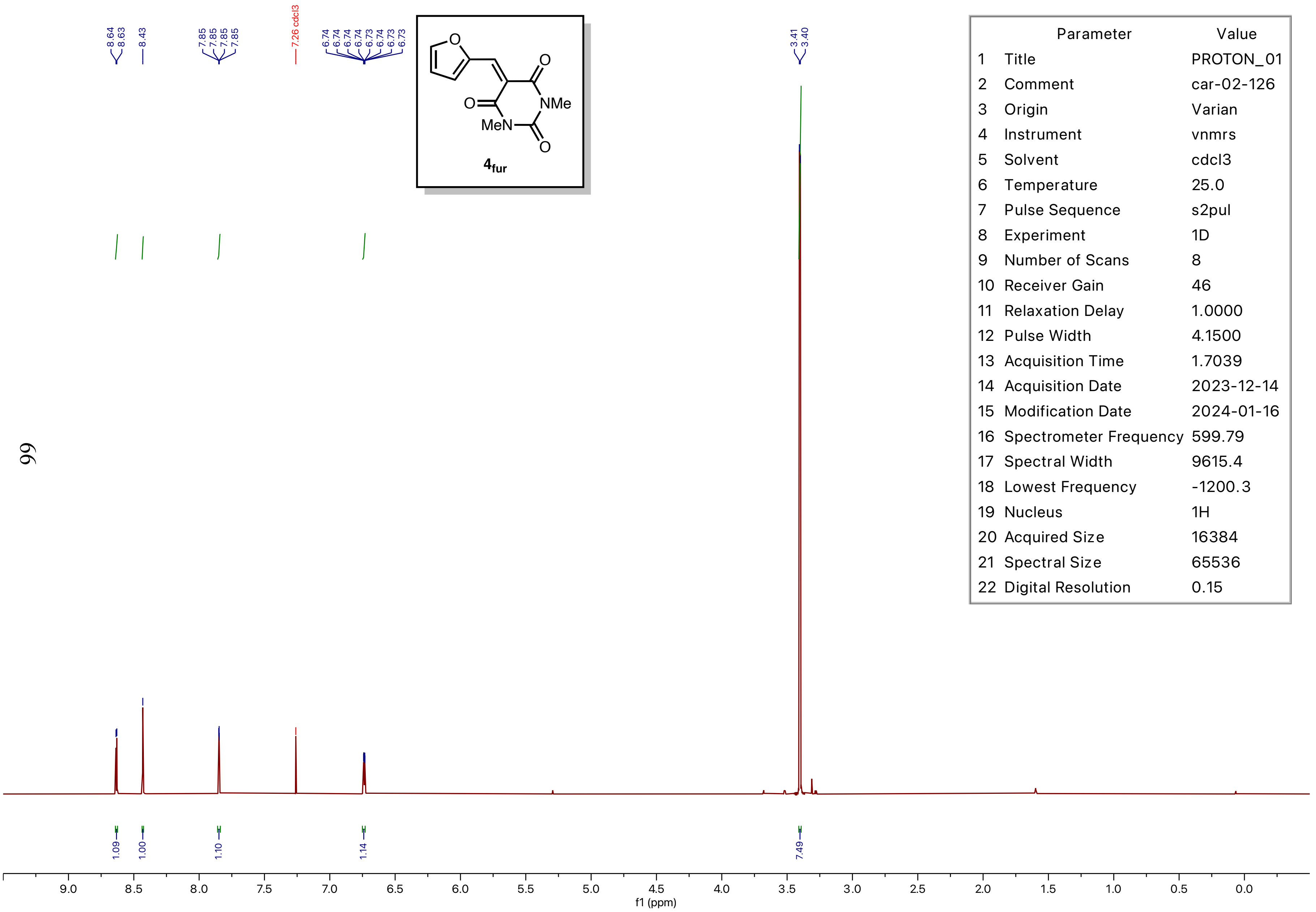

	Parameter	Value
1	Title	PROTON_01
2	Comment	car-02-126
3	Origin	Varian
4	Instrument	vnmrs
5	Solvent	cdcl3
6	Temperature	25.0
7	Pulse Sequence	s2pul
8	Experiment	1D
9	Number of Scans	8
10	Receiver Gain	46
11	Relaxation Delay	1.0000
12	Pulse Width	4.1500
13	Acquisition Time	1.7039
14	Acquisition Date	2023-12-14
15	Modification Date	2024-01-16
16	Spectrometer Frequency	599.79
17	Spectral Width	9615.4
18	Lowest Frequency	-1200.3
19	Nucleus	1H
20	Acquired Size	16384
21	Spectral Size	65536
22	Digital Resolution	0.15

Supplementary Fig. 63. **^{1}H NMR (600 MHz, $CDCl_3$) of activated furan 4_{fur}.**

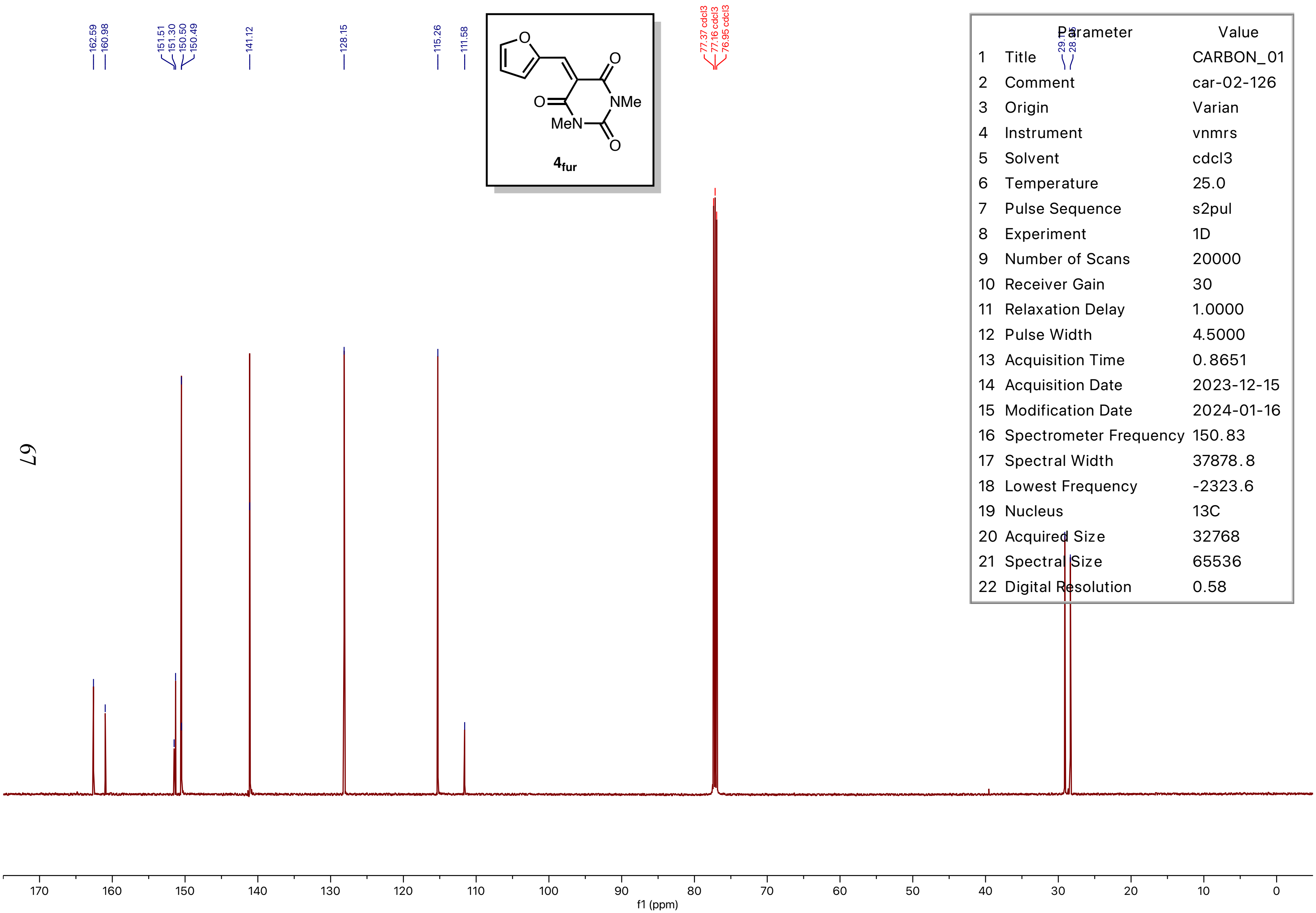

Supplementary Fig. 64. **^{13}C NMR (150 MHz, $CDCl_3$) of activated furan 4_{fur}.**

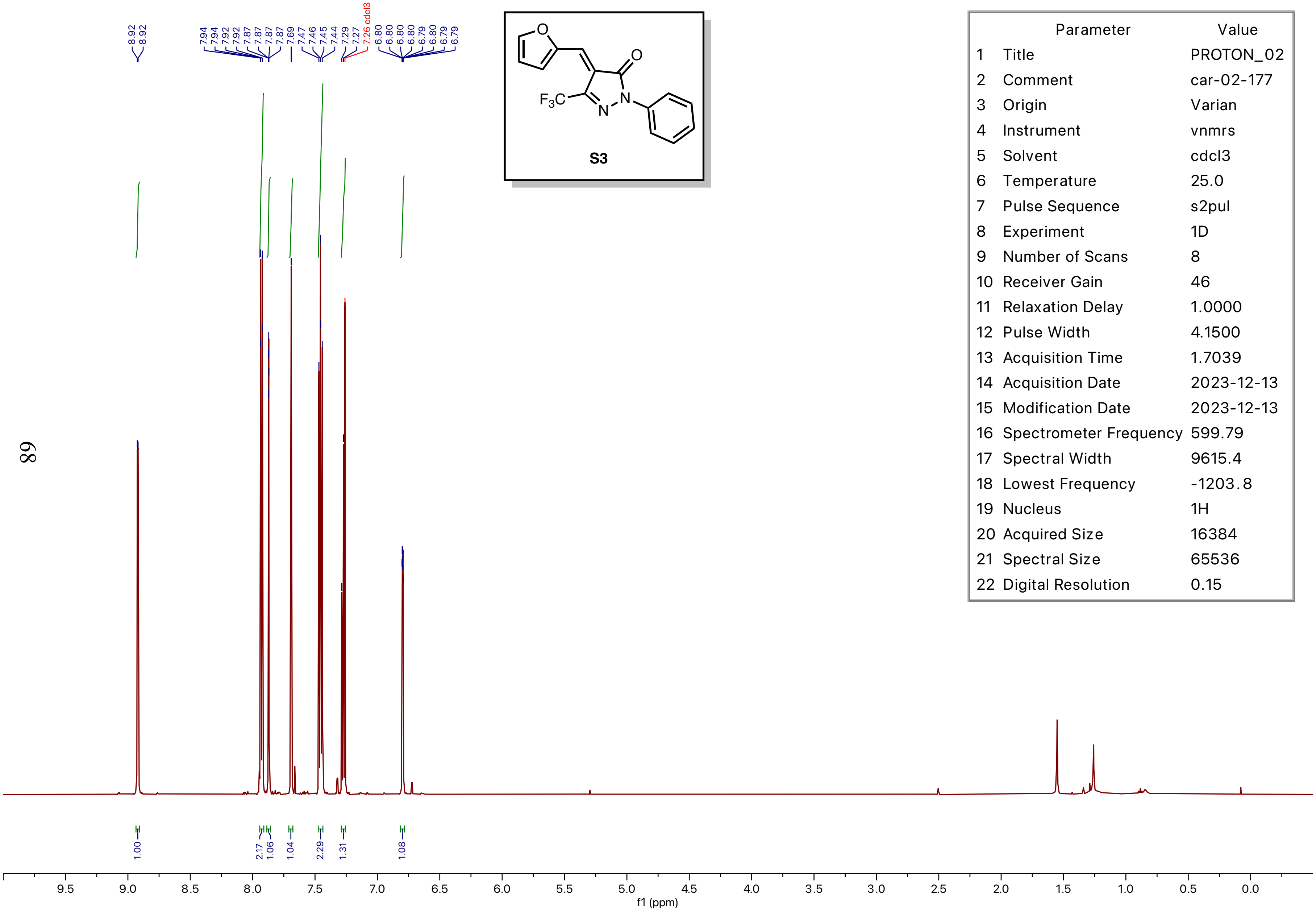

	Parameter	Value
1	Title	PROTON_02
2	Comment	car-02-177
3	Origin	Varian
4	Instrument	vnmrs
5	Solvent	cdcl3
6	Temperature	25.0
7	Pulse Sequence	s2pul
8	Experiment	1D
9	Number of Scans	8
10	Receiver Gain	46
11	Relaxation Delay	1.0000
12	Pulse Width	4.1500
13	Acquisition Time	1.7039
14	Acquisition Date	2023-12-13
15	Modification Date	2023-12-13
16	Spectrometer Frequency	599.79
17	Spectral Width	9615.4
18	Lowest Frequency	-1203.8
19	Nucleus	1H
20	Acquired Size	16384
21	Spectral Size	65536
22	Digital Resolution	0.15

Supplementary Fig. 65. **^{1}H NMR (600 MHz, $CDCl_3$) of activated furan S3.**

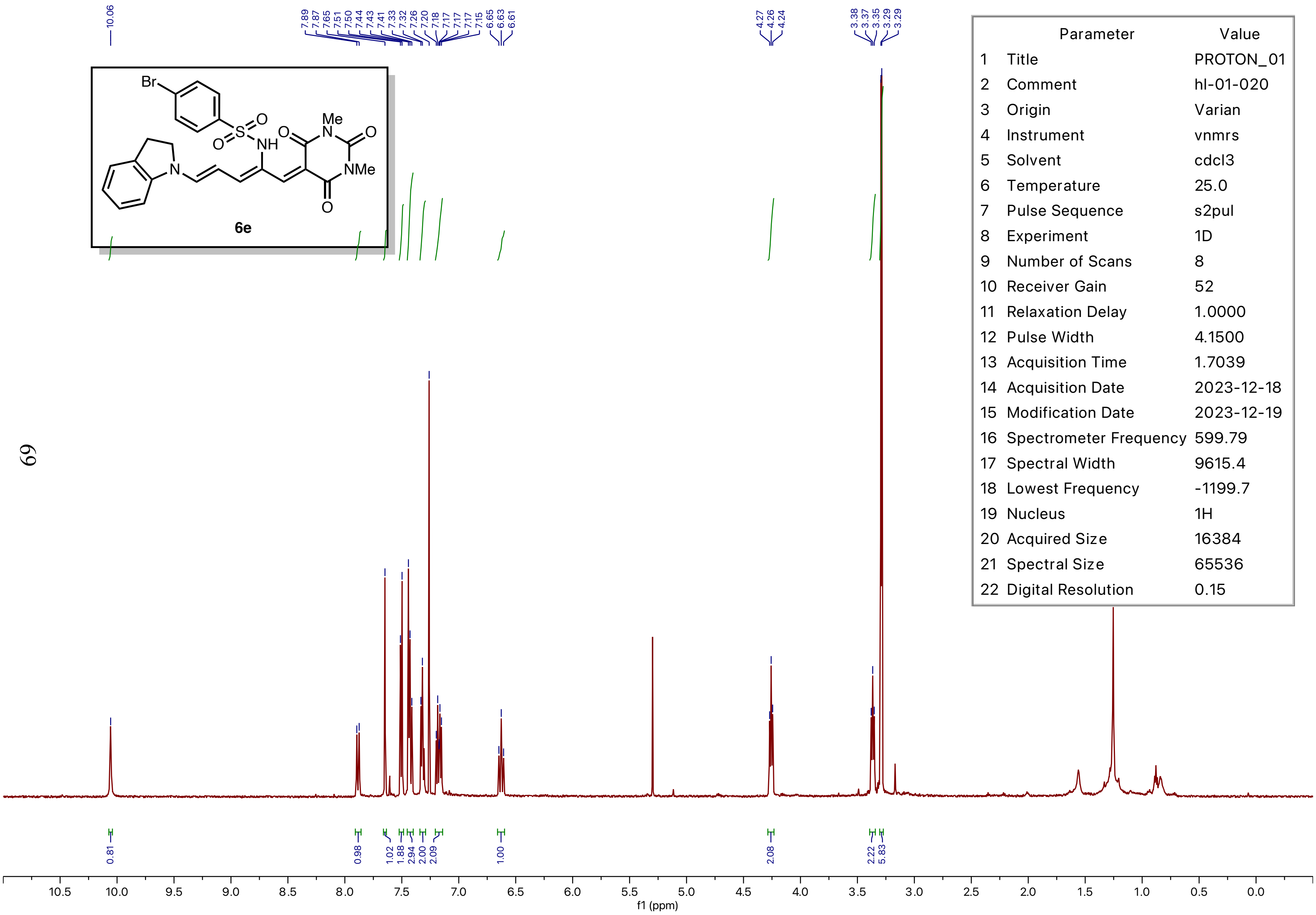

Supplementary Fig. 66. ^{1}**H NMR (600 MHz, CDCl$_3$) of amino DASA 6e.**

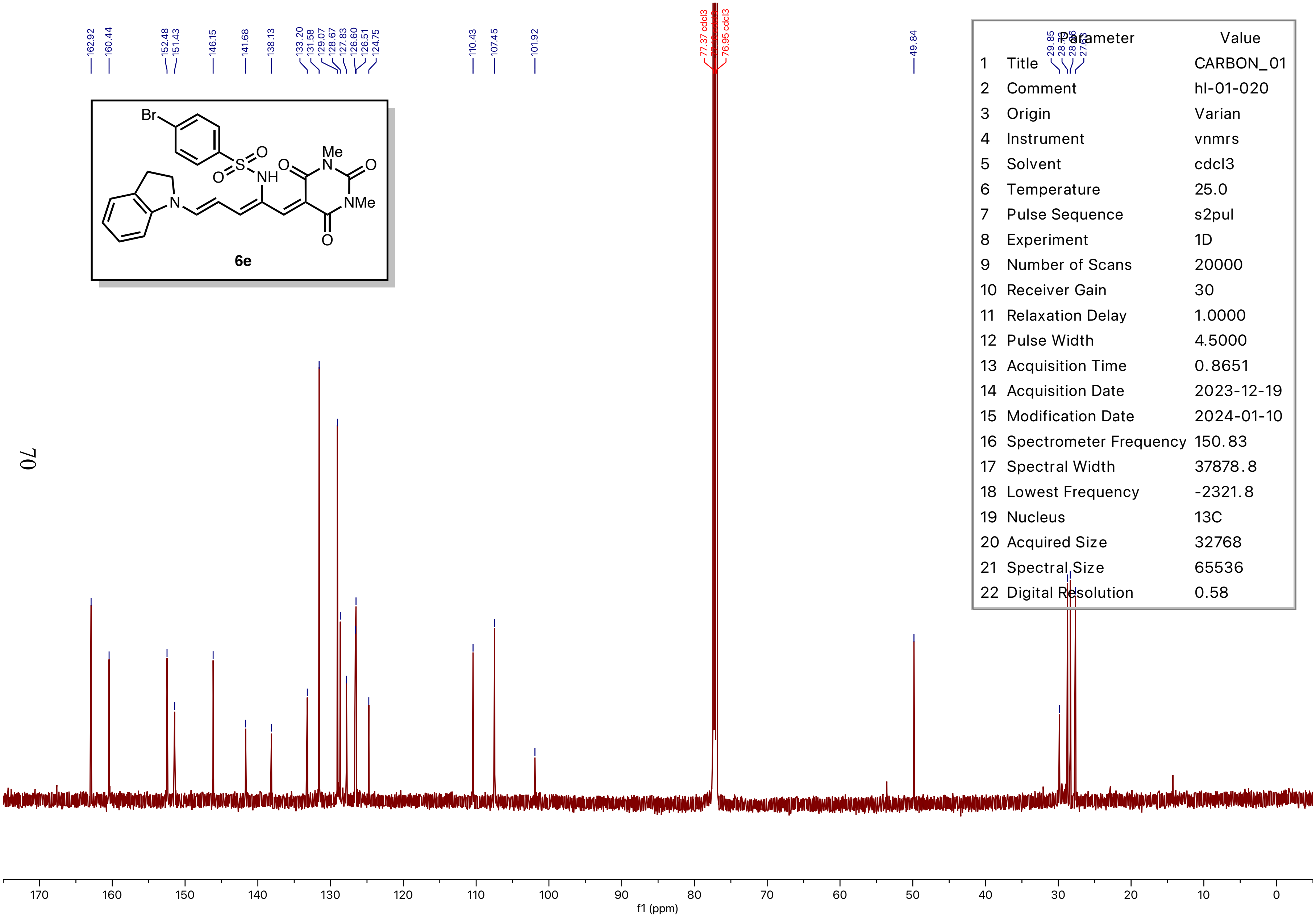

Supplementary Fig. 67. **^{13}C NMR (150 MHz, $CDCl_3$) of amino DASA 6e.**

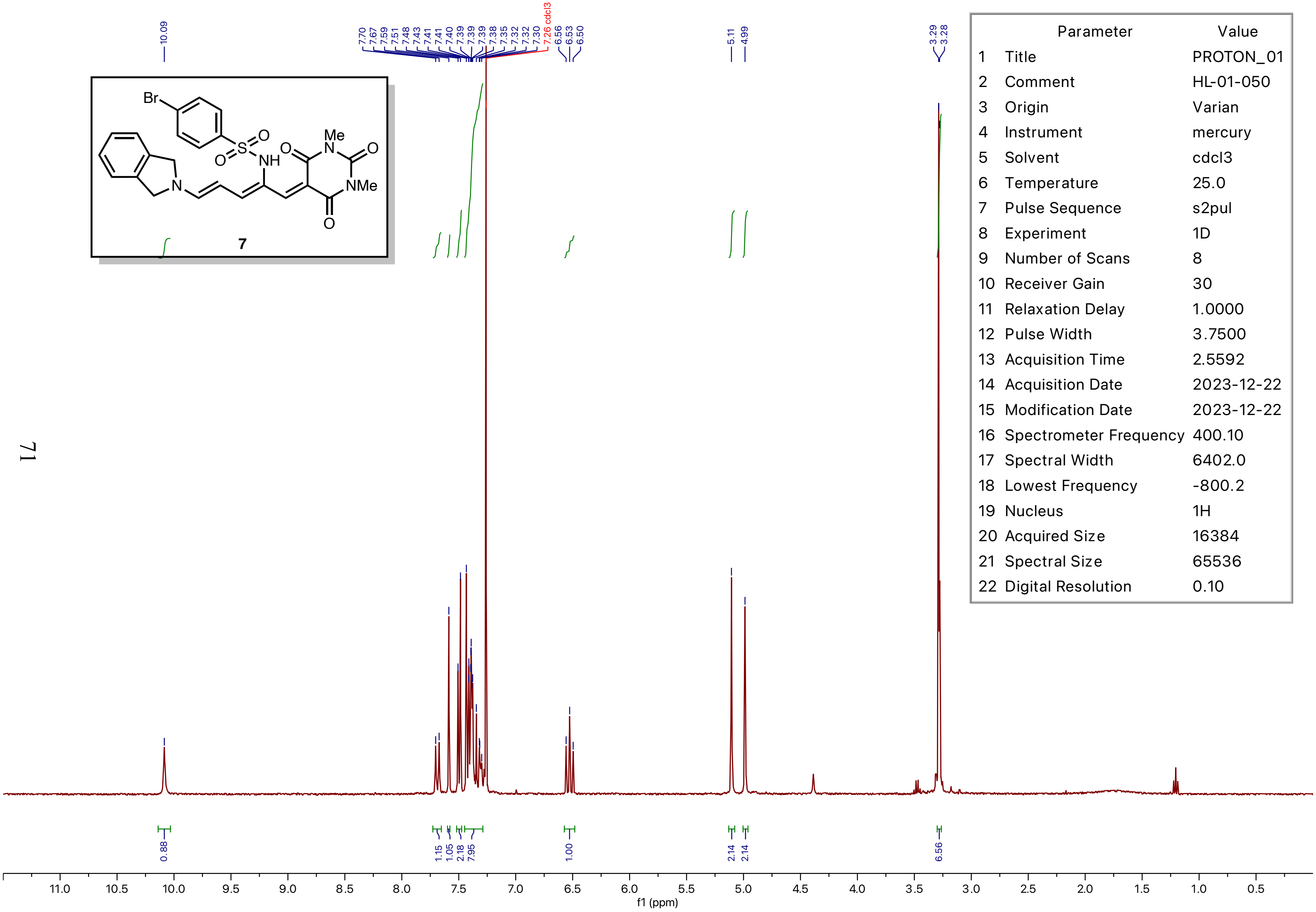

	Parameter	Value
1	Title	PROTON_01
2	Comment	HL-01-050
3	Origin	Varian
4	Instrument	mercury
5	Solvent	cdcl3
6	Temperature	25.0
7	Pulse Sequence	s2pul
8	Experiment	1D
9	Number of Scans	8
10	Receiver Gain	30
11	Relaxation Delay	1.0000
12	Pulse Width	3.7500
13	Acquisition Time	2.5592
14	Acquisition Date	2023-12-22
15	Modification Date	2023-12-22
16	Spectrometer Frequency	400.10
17	Spectral Width	6402.0
18	Lowest Frequency	-800.2
19	Nucleus	1H
20	Acquired Size	16384
21	Spectral Size	65536
22	Digital Resolution	0.10

Supplementary Fig. 68. **^{1}H NMR (400 MHz, $CDCl_3$) of amino DASA 7.**

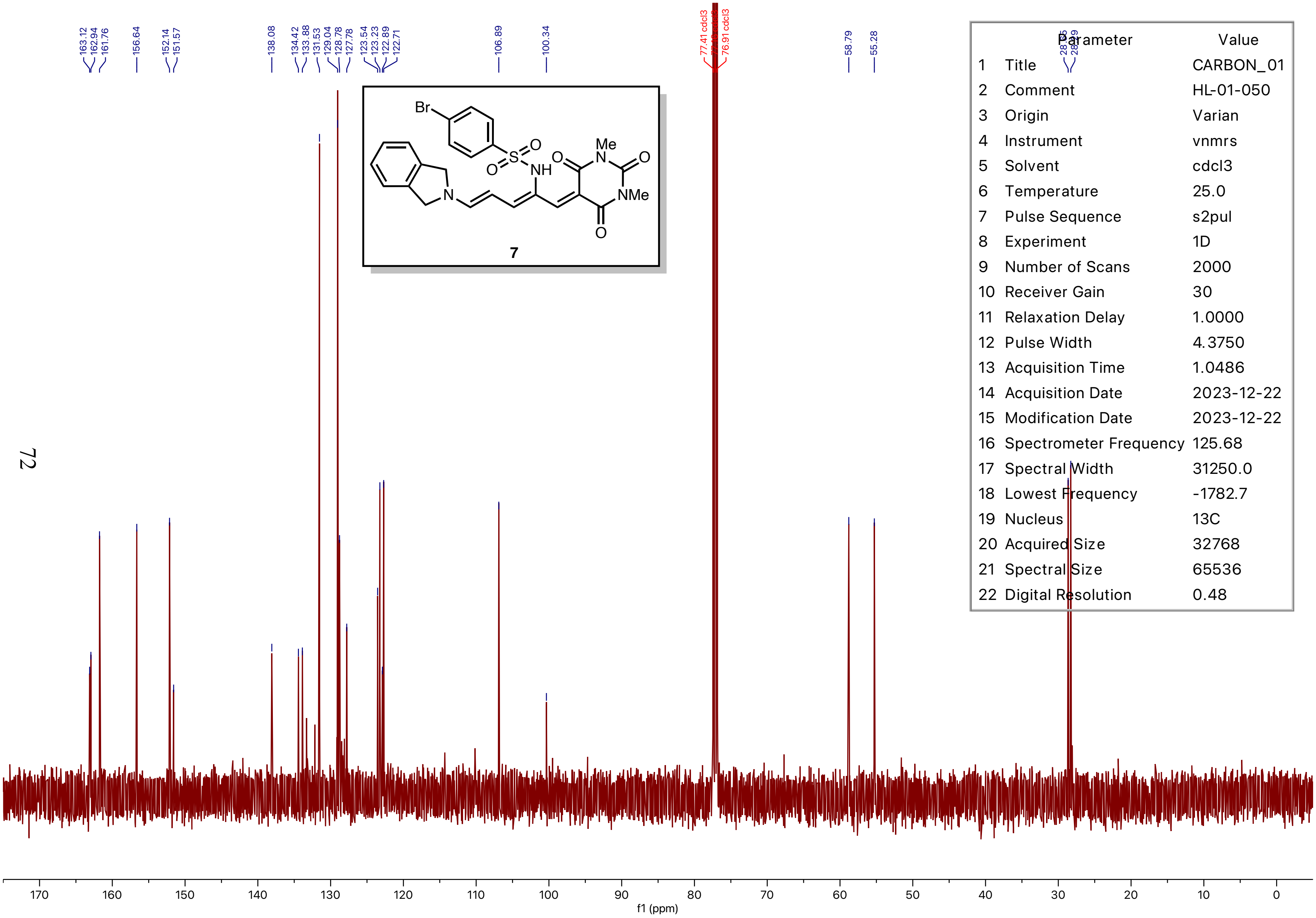

Supplementary Fig. 69. **^{13}C NMR (125 MHz, $CDCl_3$) of amino DASA 7.**

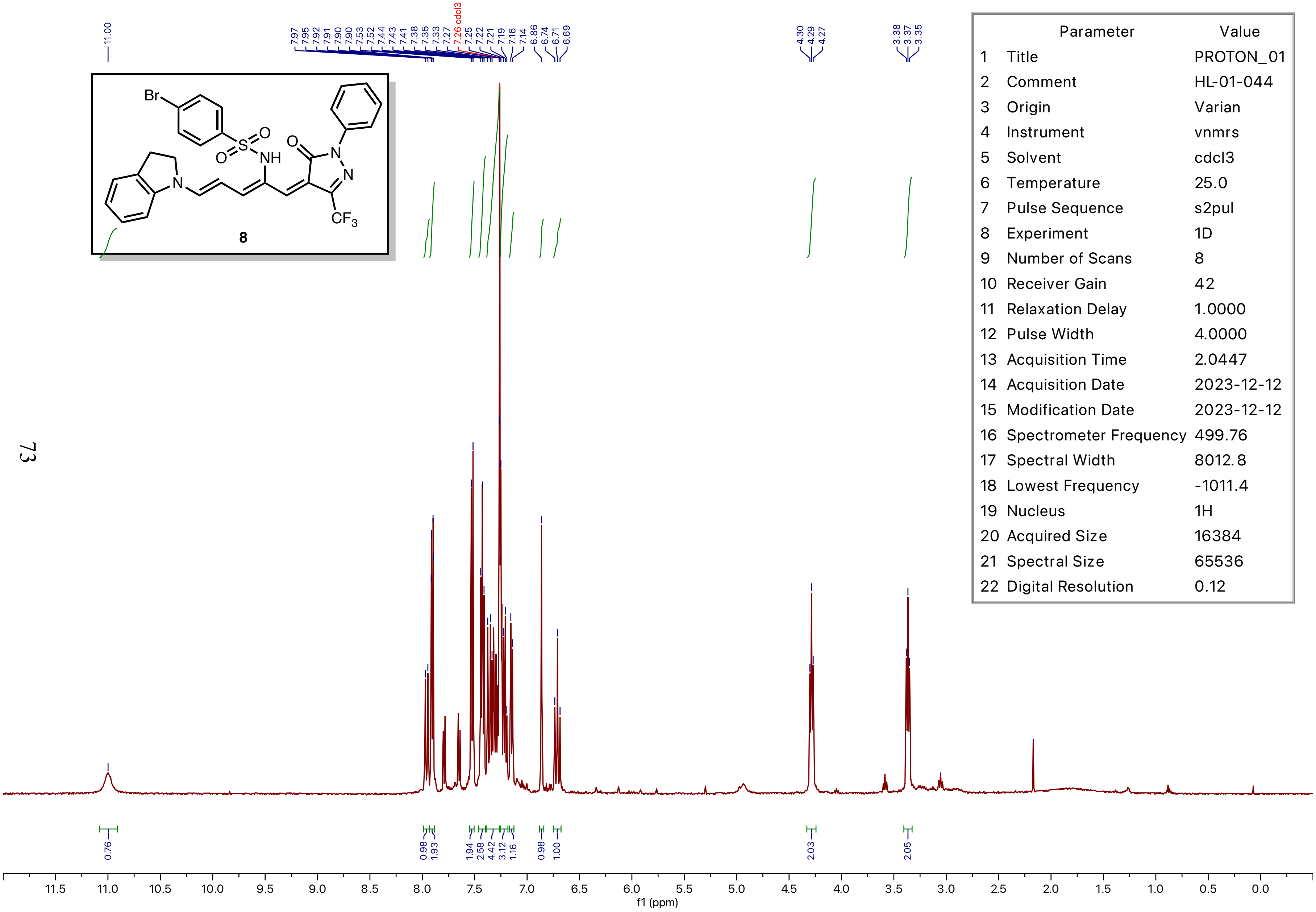

Supplementary Fig. 70. **^{1}H NMR (500 MHz, $CDCl_3$) of amino DASA 8.**

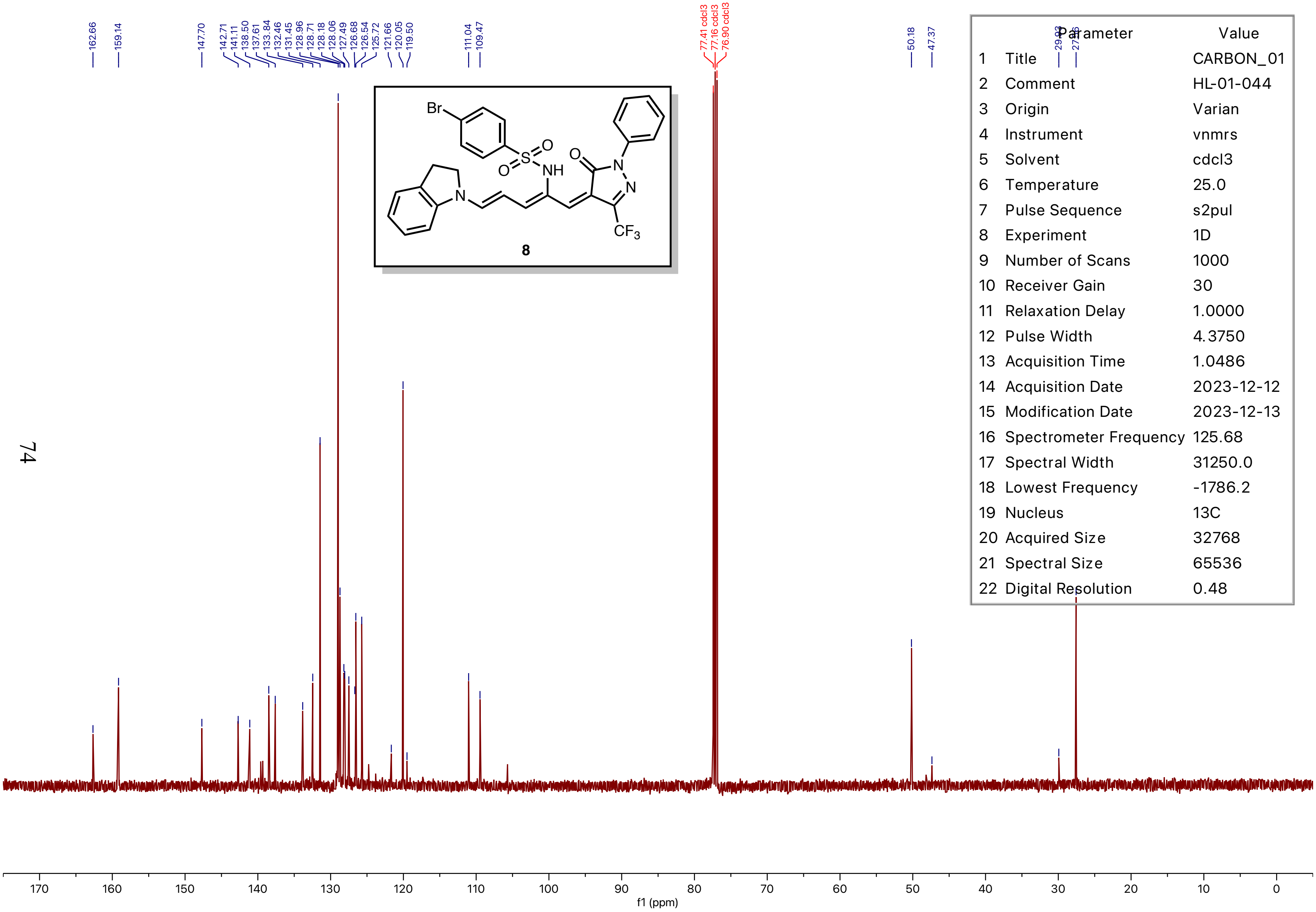

	Parameter	Value
1	Title	CARBON_01
2	Comment	HL-01-044
3	Origin	Varian
4	Instrument	vnmrs
5	Solvent	cdcl3
6	Temperature	25.0
7	Pulse Sequence	s2pul
8	Experiment	1D
9	Number of Scans	1000
10	Receiver Gain	30
11	Relaxation Delay	1.0000
12	Pulse Width	4.3750
13	Acquisition Time	1.0486
14	Acquisition Date	2023-12-12
15	Modification Date	2023-12-13
16	Spectrometer Frequency	125.68
17	Spectral Width	31250.0
18	Lowest Frequency	-1786.2
19	Nucleus	13C
20	Acquired Size	32768
21	Spectral Size	65536
22	Digital Resolution	0.48

Supplementary Fig. 71. **^{13}C NMR (125 MHz, $CDCl_3$) of amino DASA 8.**

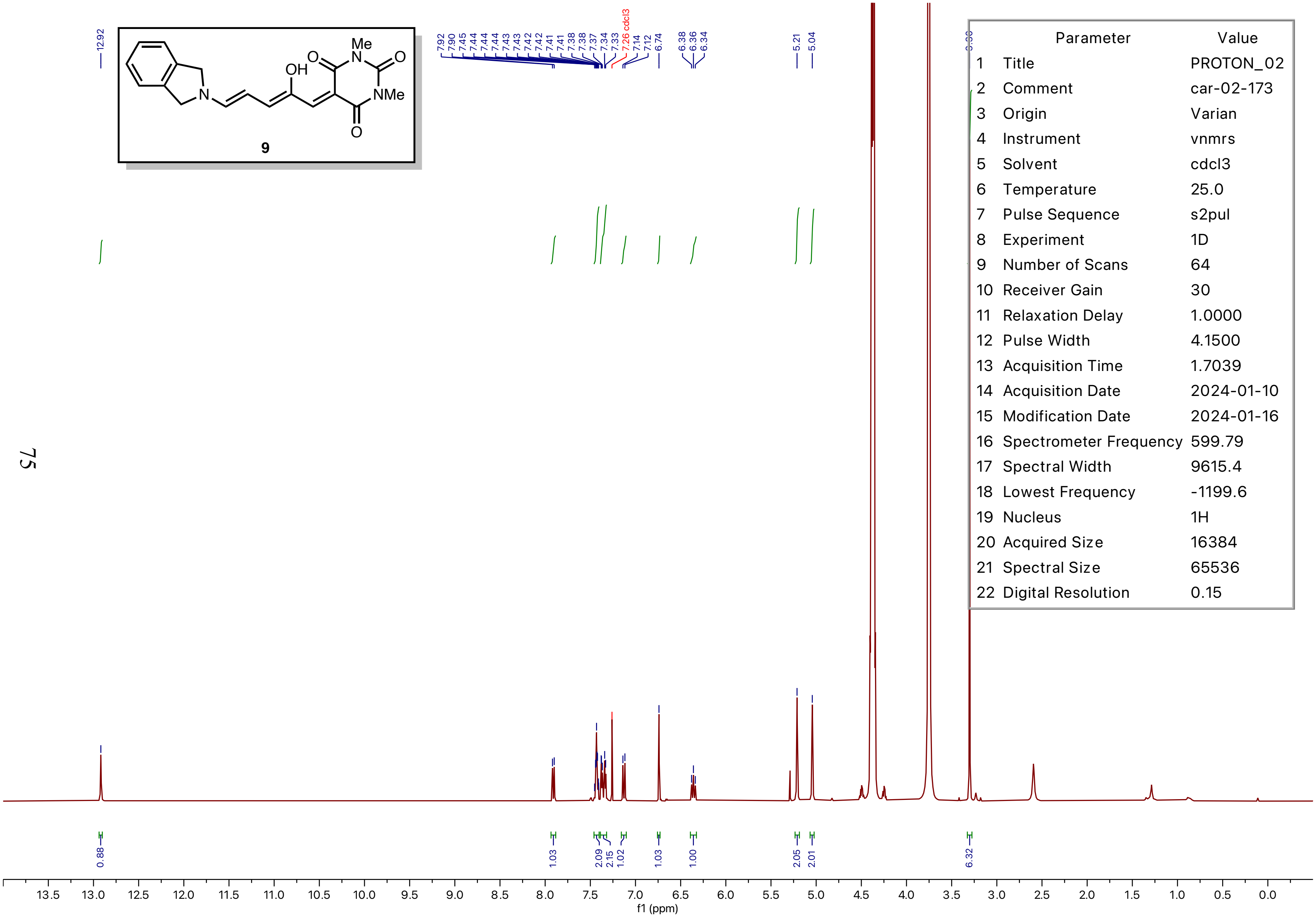

Supplementary Fig. 72. **^{1}H NMR (600 MHz, $CDCl_3$) of hydroxy DASA 9.**

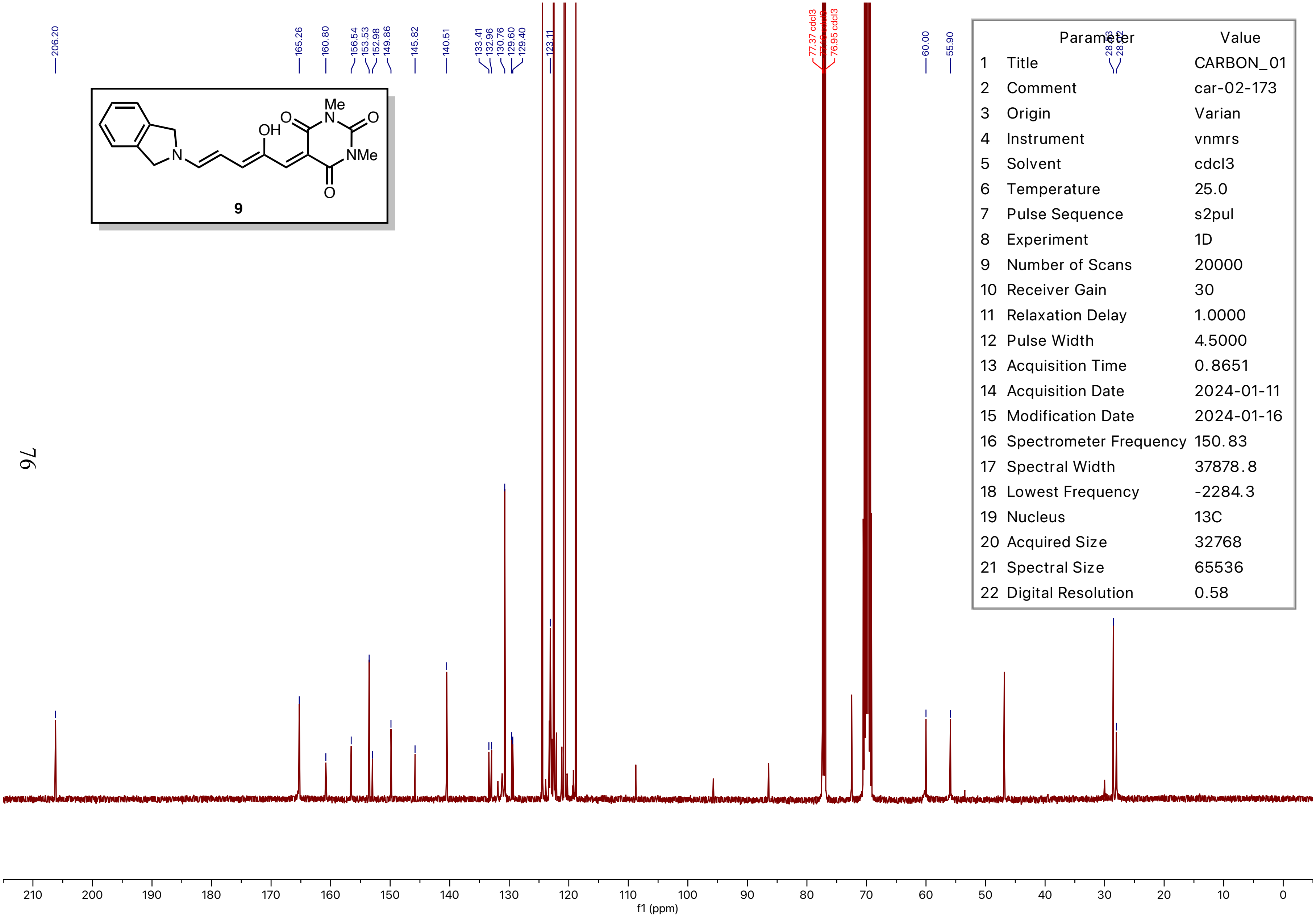

	Parameter	Value
1	Title	CARBON_01
2	Comment	car-02-173
3	Origin	Varian
4	Instrument	vnmrs
5	Solvent	cdcl3
6	Temperature	25.0
7	Pulse Sequence	s2pul
8	Experiment	1D
9	Number of Scans	20000
10	Receiver Gain	30
11	Relaxation Delay	1.0000
12	Pulse Width	4.5000
13	Acquisition Time	0.8651
14	Acquisition Date	2024-01-11
15	Modification Date	2024-01-16
16	Spectrometer Frequency	150.83
17	Spectral Width	37878.8
18	Lowest Frequency	-2284.3
19	Nucleus	13C
20	Acquired Size	32768
21	Spectral Size	65536
22	Digital Resolution	0.58

Supplementary Fig. 73. **^{13}C NMR (150 MHz, $CDCl_3$) of hydroxy DASA 9.**

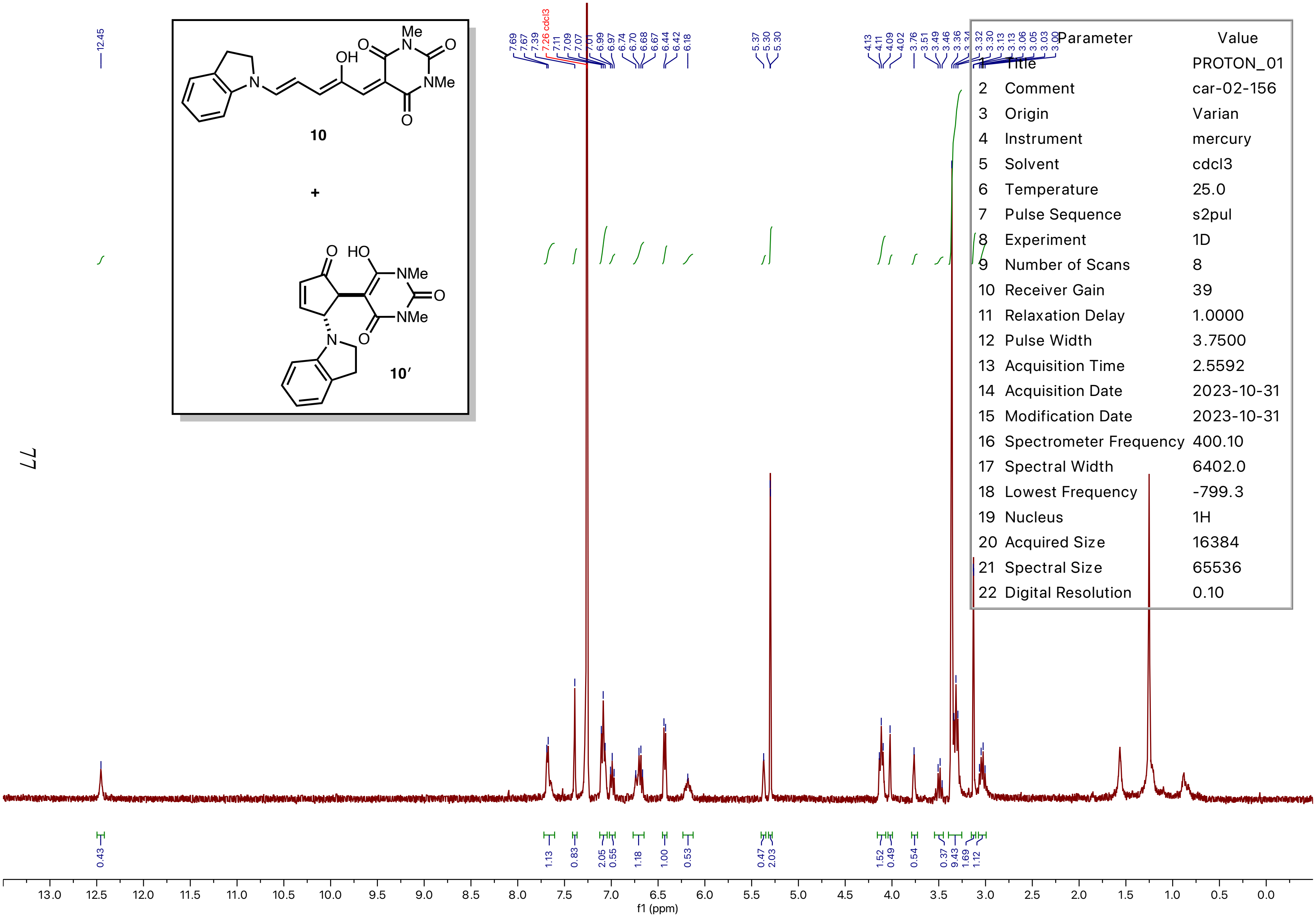

Supplementary Fig. 74. **^{1}H NMR (400 MHz, $CDCl_3$) of hydroxy DASA 10.**

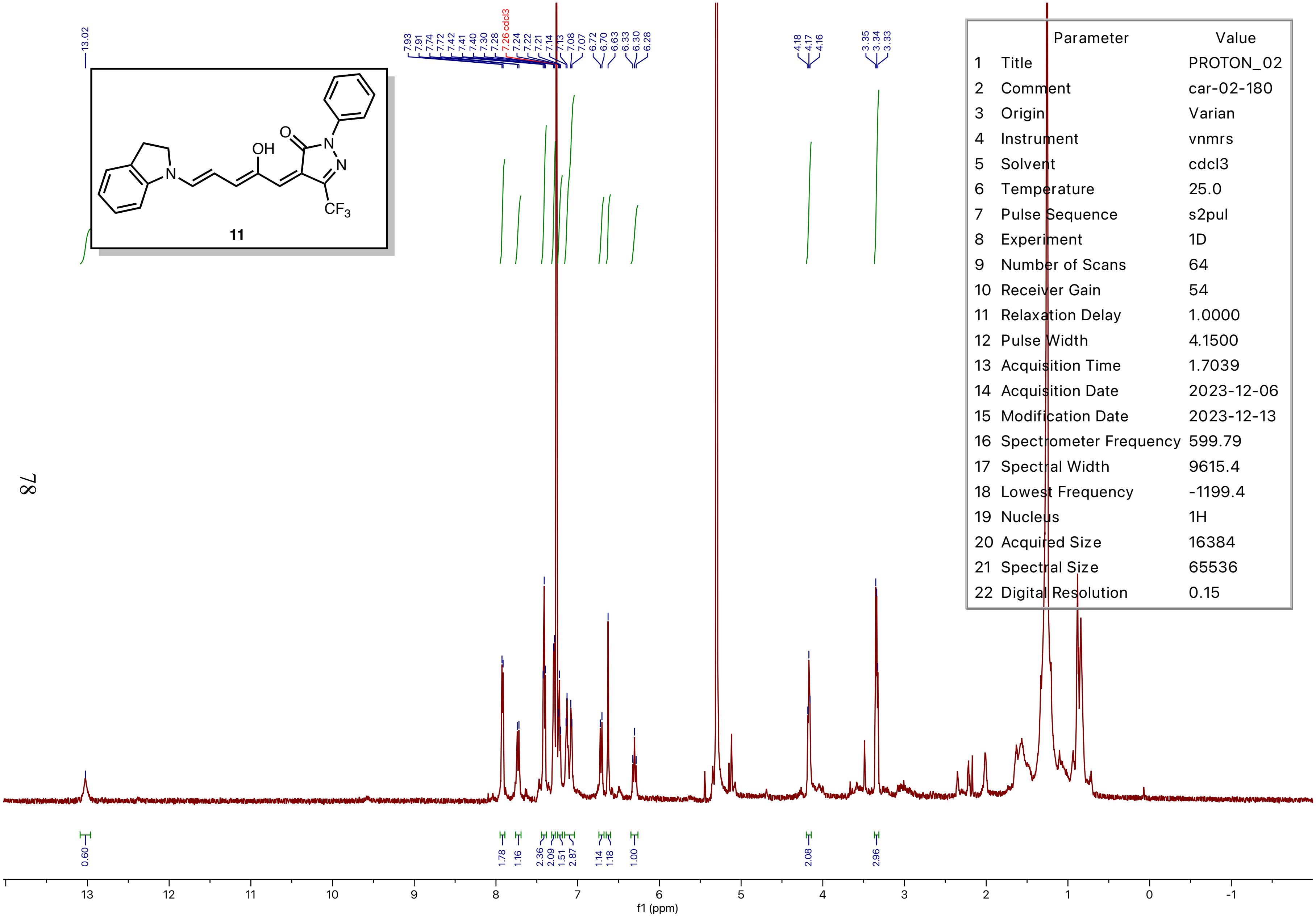

Supplementary Fig. 75. **^{1}H NMR (600 MHz, $CDCl_3$) of hydroxy DASA 11.**

2. Supplementary References

1. Stricker, F., Seshadri, S. & Read de Alaniz, J. Donor-Acceptor Stenhouse Adducts in Molecular Photoswitches: Chemistry, Properties, and Applications. *Wiley-VCH GmbH* **1**, 303–324 (2022).
2. Yu, D., Thai, V. T., Palmer, L. I., Veits, G. K., Cook, J. E., Read de Alaniz, J. & Hein, J. E. Importance of Off-Cycle Species in the Acid-Catalyzed Aza-Piancatelli Rearrangement. *J. Org. Chem*. **78**, 12784–127879 (2013).
3. Gomes, R. F. A., Coelho, J. A. S. & Alfonso, C. A. M. Synthesis and Applications of Stenhouse Salts and Derivatives. *Chem. Eur. J.* **24**, 9170–9186 (2018).
4. Clerc, M., Stricker, F., Ulrich, S., Sroda, M., Bruns, N., Boesel, L. F. & Read de Alaniz, J. Promoting the Furan Ring-Opening Reaction to Access New Donor–Acceptor Stenhouse Adducts with Hexafluoroisopropanol. *Angew. Chem. Int. Ed.* **60**, 10219–10227 (2021).
5. Reinus, B. J. & Kerwin, S. M. *N*-Alkynyl Pyrrole Based Total Synthesis of Shensongine A. *Synthesis* **51**, 4085–4105 (2019).
6. Abell, A. D. & Litten, J. C. Synthesis and Amino Acid Chain Extension of 1-Acylated Hydroxymethylpyrroles. *Aust. J. Chem.* **46**, 1473–1483 (1993).
7. Nemykin, V. N., Schrage, B. R. & Ziegler, C, J. Structure and Electronics in 1H-pyrrol-2-ylmethylene Compounds. *Tetrahedron* **76**, 131149 (2020).
8. Ogiwara, Y., Takahashi, K., Kitazawa, T. & Sakai, N. Indium(III)-Catalyzed Knoevenagel Condensation of Aldehydes and Activated Methylenes Using Acetic Anhydride as a Promoter. *J. Org. Chem.* **80**, 3101–3110 (2015).
9. Noirbent, G., Xu, Y., Bonardi, A.-H., Duval, S., Gigmes, D., Lalevée, J. & Dumur, F. New Donor-Acceptor Stenhouse Adducts as Visible and Near Infrared Light Polymerization Photoinitiators. *Molecules* **25**, 2317 (2020).
10. Hemmer, J. R., Poelma, S. O., Treat, N., Page, Z. A., Dolinski, N. D., Diaz, Y. J., Tomlinson, W., Clark, K. D., Hooper, J. P., Hawker, C. & Read de Alaniz, J. Tunable Visible and Near Infrared Photoswitches. *J. Am. Chem. Soc.* **138**, 13960–13966 (2016).
11. Sroda, M. M. Stricker, F., Peterson, J. A., Bernal, A. & Read de Alaniz, J. Donor–Acceptor Stenhouse Adducts: Exploring the Effects of Ionic Character. *Chem. Eur. J.* **27**, 4183–4190 (2021).
12. PhysChem Suite, Software V11.02, Advanced Chemistry Development, Inc (ACD/Labs), Toronto, ON, Canada, www.acdlabs.com
13. Feldmeier, C. Bartling, H., Riedle, E. & Gschwind, R. M. LED based NMR illumination device for mechanistic studies on photochemical reactions – Versatile and simple, yet surprisingly powerful. *J. Magn. Reson.* **232**, 39–44 (2013).
